# Network analysis of transcriptomic diversity amongst resident tissue macrophages and dendritic cells in the mouse mononuclear phagocyte system

**DOI:** 10.1371/journal.pbio.3000859

**Published:** 2020-10-08

**Authors:** Kim M. Summers, Stephen J. Bush, David A. Hume

**Affiliations:** 1 Mater Research Institute-University of Queensland, Translational Research Institute, Brisbane, Queensland, Australia; 2 Nuffield Department of Clinical Medicine, John Radcliffe Hospital, University of Oxford, Oxford, United Kingdom; National Jewish Medical and Research Center/Howard Hughes Medical Institute, UNITED STATES

## Abstract

The mononuclear phagocyte system (MPS) is a family of cells including progenitors, circulating blood monocytes, resident tissue macrophages, and dendritic cells (DCs) present in every tissue in the body. To test the relationships between markers and transcriptomic diversity in the MPS, we collected from National Center for Biotechnology Information Gene Expression Omnibus (NCBI-GEO) a total of 466 quality RNA sequencing (RNA-seq) data sets generated from mouse MPS cells isolated from bone marrow, blood, and multiple tissues. The primary data were randomly downsized to a depth of 10 million reads and requantified. The resulting data set was clustered using the network analysis tool *BioLayout*. A sample-to-sample matrix revealed that MPS populations could be separated based upon tissue of origin. Cells identified as classical DC subsets, cDC1s and cDC2s, and lacking *Fcgr1* (encoding the protein CD64) were contained within the MPS cluster, no more distinct than other MPS cells. A gene-to-gene correlation matrix identified large generic coexpression clusters associated with MPS maturation and innate immune function. Smaller coexpression gene clusters, including the transcription factors that drive them, showed higher expression within defined isolated cells, including monocytes, macrophages, and DCs isolated from specific tissues. They include a cluster containing *Lyve1* that implies a function in endothelial cell (EC) homeostasis, a cluster of transcripts enriched in intestinal macrophages, and a generic lymphoid tissue cDC cluster associated with *Ccr7*. However, transcripts encoding *Adgre1*, *Itgax*, *Itgam*, *Clec9a*, *Cd163*, *Mertk*, *Mrc1*, *Retnla*, and *H2-a/e* (encoding class II major histocompatibility complex [MHC] proteins) and many other proposed macrophage subset and DC lineage markers each had idiosyncratic expression profiles. Coexpression of immediate early genes (for example, *Egr1*, *Fos*, *Dusp1*) and inflammatory cytokines and chemokines (tumour necrosis factor [*Tnf*], *Il1b*, *Ccl3/4*) indicated that all tissue disaggregation and separation protocols activate MPS cells. Tissue-specific expression clusters indicated that all cell isolation procedures also co-purify other unrelated cell types that may interact with MPS cells in vivo. Comparative analysis of RNA-seq and single-cell RNA-seq (scRNA-seq) data from the same lung cell populations indicated that MPS heterogeneity implied by global cluster analysis may be even greater at a single-cell level. This analysis highlights the power of large data sets to identify the diversity of MPS cellular phenotypes and the limited predictive value of surface markers to define lineages, functions, or subpopulations.

## Abstract 2

The mononuclear phagocyte system is a family of cells present in every tissue in the body. A comprehensive network analysis of public transcriptomic data reveals the close relationships between tissue-resident cells of the mononuclear phagocyte system (macrophages and dendritic cells) and questions the utility of surface markers to define lineages, functions, or subpopulations.

## Introduction

The mononuclear phagocyte system (MPS) [1] is a family of cells including progenitors, circulating blood monocytes, resident tissue macrophages, and dendritic cells (DCs) that are present in every tissue in the body [2–5]. Within each tissue, resident macrophages occupy niches or territories with a remarkably regular distribution (reviewed in [5, 6]). The proliferation, differentiation, and survival of most resident macrophage populations depends upon signals from the macrophage-colony–stimulating factor receptor (CSF1R) initiated by one of 2 ligands, CSF1 or IL34 [7, 8]. Based upon detection of macrophage-restricted mRNA, including *Csf1r*, the relative abundance of resident macrophages in most organs in mice was shown to reach a maximum in the first week of postnatal life and remains stable thereafter during postnatal growth [9]. Lineage-trace studies in the C57BL/6 strain suggest that many macrophage populations established in the mouse embryo are maintained in adults mainly by self-renewal, whereas others are replaced progressively to differing extents by blood monocytes derived from bone marrow progenitors throughout life [10–12]. Most, if not all, tissue macrophage populations can be generated and maintained in the absence of blood monocytes because of the intrinsic homeostatic regulation by circulating CSF1 [13]. The precise details of ontogeny, turnover, and homeostasis of resident macrophages may not be conserved across mouse strains or species [5]. However, regardless of their steady-state turnover, all tissue-resident macrophages, including the microglia of the brain, can also be rapidly replaced by blood monocytes following experimental depletion ([3–6, 13] and references therein).

Within individual tissues, resident macrophages acquire specific adaptations and gene expression profiles [2, 4, 5, 14–16]. These adaptations contribute to survival as well as function and involve inducible expression of transcription factors and their downstream target genes. At least some of these transcription factors act by regulating *Csf1r* expression. Deletion of a conserved enhancer in the mouse *Csf1r* gene leads to selective loss of some tissue macrophage populations, whereas others express *Csf1r* normally and are unaffected [17]. In the mouse embryo, where abundant macrophage populations are engaged with phagocytosis of apoptotic cells [18], the macrophage transcriptome does not differ greatly between organs. Tissue-specific macrophage adaptation occurs mainly in the postnatal period, as the organs themselves exit the proliferative phase and start to acquire adult function [9, 16].

Classical DCs (cDCs) are commonly defined functionally on the basis of a proposed unique ability to present antigen to naïve T cells, a concept that requires a clear distinction between DCs and macrophages [19]. The situation is confused by the widespread use of the term DC to describe any antigen-presenting cell (APC), including cells that are clearly derived from blood monocytes [20]. An attempt at consensus proposed an MPS nomenclature classification based upon ontogeny and secondarily upon location, function, and phenotype [21]. The proposal separates monocyte-derived APCs from cDC subsets: cDC1s, dependent on the transcription factor BATF3, and cDC2s, dependent upon IRF4. Some support for this separation came from analysis of an *Ms4a3* reporter transgene, which labelled cells derived from committed granulocyte-macrophage (GM) progenitors and distinguished monocyte-derived cells from tissue DCs [11]. Secondary classification is based upon cell surface markers that are presumed to be linked in some way to ontogeny. The proposed development pathway of these DC subsets from a common myeloid progenitor via a common DC progenitor (CDP) has been reviewed recently [22]. However, it remains unclear as to whether cDCs should be considered part of the MPS and the extent to which they can be separated from other MPS cells based upon surface markers [13].

Even within individual tissues, resident macrophages are extremely heterogeneous [23, 24]. Since the advent of monoclonal antibodies and later development of transgenic reporter genes [25], numerous markers have been identified that segregate the MPS into subpopulations. More recently, mouse tissue macrophage heterogeneity has been analysed using multiparameter flow cytometry and single-cell RNA sequencing (scRNA-seq) [26]. Amongst the recent suggestions, LYVE1 was proposed as a marker of macrophages associated with the vasculature [27], CD64 (*Fcgr1* gene) and MERTK as markers that distinguish macrophages from cDCs [28, 29], and CD206 (*Mrc1* gene) as a marker of so-called M2 macrophage polarisation [30, 31]. Several surface markers have also been identified that are encoded by genes expressed only in macrophages in specific tissues (for example, *Clec4f*, *Tmem119*, *Siglecf*) [16, 32]. Other markers define macrophages in specific locations within a tissue, for example, CD169 (encoded by *Siglec1*) in the marginal zone of spleen and haematopoietic islands in bone marrow [33]. In the case of blood monocytes, the subpopulations are clearly a differentiation series in which short-lived LY6C^hi^ ‘classical’ monocytes give rise in a CSF1R-dependent manner [34] to long-lived LY6C^lo^ nonclassical monocytes via an intermediate state [12, 34, 35]. This is likely also the case in tissues such as the liver [32] and intestine [36, 37].

Mechanistically, the association between marker expression and cellular function depends upon coordinated transcriptional regulation. One way to identify coregulated sets of transcripts is to cluster large transcriptomic data sets. This approach was used to create transcriptional atlases in multiple species and identify lineage-specific transcription factors and their target genes [38–42]. It enabled the extraction of a generic tumour-associated macrophage signature from multiple large cancer data sets [43]. Previous meta-analysis of large microarray data sets [38, 39, 42], as well as a reanalysis of data from the ImmGen Consortium [44], indicated a clear separation in the mouse of MPS cells from all other leukocyte lineages but did not support the basic premise that markers can separate macrophages from DCs or define lineages within the MPS.

Over the past 5 years, RNA-seq has supplanted microarrays as an approach to expression profiling. The recent cascade of interest in tissue-specific macrophage adaptation has produced RNA-seq data for MPS cells isolated from most major organs of C57BL/6 mice. There has been no previous effort to integrate this data deluge into a cohesive view of MPS transcriptional diversity and to identify sets of transcripts that are stringently co-ordinately regulated. To enable comparative analysis of data sets from multiple laboratories, we devised an automated informatics pipeline employing random sampling of RNA-seq data to a common depth and quantification using the pseudoaligner Kallisto. Robust transcriptional atlases for the chicken [45] and pig [46] were generated using data sets from numerous divergent sources. The analysis of these merged data sets, as well as large multitissue data sets from sheep, human, and water buffalo [47–49], provided strong support for the principle of guilt by association, namely that genes that contribute to a specific biological function tend to be coregulated. Each of these analyses identified transcripts that were enriched specifically in MPS cells relative to other haematopoietic cells. Using the same basic pipeline as in the chicken and pig projects, we identified a total of 466 RNA-seq libraries generated from isolated macrophage and cDC populations from 24 different studies that sample mouse MPS transcriptional diversity (Table 1). Here, we apply network clustering to this large data set to identify shared and divergent transcriptional adaptation of tissue-resident MPS cells and revisit the relationships between macrophages and DCs.

**Table 1:** GEO and BioProject accession numbers for samples used in the analysis. SRA and NCBI accessions and sample descriptions are available in S1 Data.

Accession	BioProject	Reference	Description (Markers Used in FACS Purification)
GSE125691	PRJNA517169	[27]	Interstitial subsets from lung, skin, fat, heart + monocytes, and alveolar macrophages (LYVE1, SIGLECF).
GSE84586	PRJNA330530	[51]	Resident macrophages from heart, kidney, and liver (F4/80, CD11B).
GSE94135	PRJNA369038	[52]	Three interstitial subsets from lung (MERTK, CD64, CD11B, CD11C, CD206, MHCII) + alveolar macrophages.
GSE95859	PRJNA378611	[53]	Brown adipose macrophages (CX3CR1-EGFP).
GSE114434	PRJNA471340	[37]	Monocytes and small intestinal macrophage subsets (CD4, TIM4, CD64).
GSE116094	PRJNA478258	[54]	Kidney-resident and monocyte-derived subpopulations, effect of ischaemia (F4/80, CD64, CD11B, CD11C, MHCII).
GSE122766	PRJNA506249	[55]	Brain microglia, bone marrow-derived brain macrophages (CD45, CD11B, CX3CR1).
GSE123021	PRJNA507265	[56]	Brain microglia, cortex, cerebellum, hippocampus, striatum (TMEM119).
GSE127980	PRJNA525977	[57]	Erythroblastic island macrophages from marrow (EPOR-EGFP, F4/80, VCAM1, SIGLEC1).
GSE135018	PRJNA557178	[58]	Alveolar macrophages and peritoneal macrophages, effect of *Bhlhe40/41* mutation (SIGLECF, CD11B, CD11C, F4/80).
GSE128662	PRJNA528430	[32]	Monocyte to KC differentiation series. Effects of *Nr1h3* and *Smad4* mutations (F4/80, CD11B, LY6C, CLEC4F).
GSE128781	PRJNA529096	[59]	Nonparenchymal brain macrophages, microglia, and peritoneal macrophages (MHCII, CD64, CD11B).
E-MTAB-6977	PRJEB27719	[36]	Macrophage subsets from intestinal lamina propria, serosa, and muscularis (CD64, CX3CR1 lineage trace).
GSE112002	PRJNA438927	[60]	Pancreatic islet and peri-islet macrophage populations. Effect of high-fat diet (F4/80, CD11B, CD11C).
GSE103847	PRJNA407286	[61]	White adipose and sympathetic neuron-associated macrophages, spleen, microglia (CD45, CX3CR1-EGFP, F4/80).
GSE68789	PRJNA283850	[62]	Mucosal and skin LCs and DCs (CD103, CD11B, EPCAM, CD207).
GSE128518	PRJNA527979	[63]	White adipose macrophages, effect of *Trem2* mutation (CD11B, F4/80).
GSE107130	PRJNA419127	[64]	Brain microglia developmental time course: male and female. Role of microbiome (CD45, CD11B, F4/80, CD64).
GSE83222	PRJNA325288	[65]	Spleen, intestine, bone marrow macrophages. Effect of engulfment of apoptotic cells (F4/80, CD11B).
GSE95702	PRJNA378162	[66]	Monocyte subsets and bone marrow progenitors. Effect of *Cebpb* mutation (CD115, CD135, LY6C, CD11B, CD11C).
GSE130201	PRJNA534273	[67]	DCs, LN, and spleen. cDC1s/cDC2s (CD11C, CD64, MHCII, CD103, TBX21).
GSE120012	PRJNA491337	[68]	Cardiac vessel macrophages (MHCII, CCR2, CD64, CD11B).
GSE140919	PRJNA519465	[69]	Monocyte engraftment of colon/ileum (CX3CR1-EGFP, CD115, LY6C, CD64).
GE131751	PRJNA 544681	[70]	Kidney-resident and monocyte-derived macrophage and DCs (F4/80, CD64, CD11B, CD11C, MHCII, CLEC9A lineage trace).

**Abbreviations:** cDC, classical DC; DC, dendritic cell; EGFP, enhanced green fluorescent protein; FACS, fluorescence activated cell sorting; GEO, Gene Expression Omnibus; KC, Kupffer cell; LC, Langherhans cell; LN, lymph node; NCBI, National Center for Biotechnology Information; SRA, Sequence Read Archive.

## Materials and methods

The RNA-seq data sets from within the BioProjects shown in Table 1 were downloaded from the European Nucleotide Archive (ENA). S1 Data contains all the Sequence Read Archive (SRA) and National Center for Biotechnology Information (NCBI) accessions and sample descriptions. Individual BioProjects differ in methods of mRNA isolation, library preparation and sequencing methods, length, depth, and strandedness, but previous analysis in other species [45, 46] indicated that they can still produce comparable expression level estimates. Prior to expression quantification and for the purpose of minimising variation between samples, all libraries were randomly downsampled to 10 million reads, 5 times each, as described previously [45, 46]. The expression levels were then requantified using Kallisto v0.44.0 [50], and the expression level was taken as the median transcripts per million (TPM) across the 5 downsampled replicates. Kallisto quantifies expression at the transcript level as TPM by building an index of k-mers from a set of reference transcripts and then ‘pseudo-aligning’ reads to it, matching k-mers in the reads to k-mers in the index. We built a custom index (k = 21) containing the combined set of 154,627 unique protein-coding transcripts from Ensembl and NCBI RefSeq, representing 24,149 protein-coding genes (*Mus musculus* annotation GRCm38.p6). Because expression is quantified relative to this index, Kallisto is robust to the presence of spurious k-mers in the reads so that ranked TPM estimates are largely unaffected by fastq preprocessing. For paired-end samples, Kallisto estimates the fragment length from the reads. For single-end samples, fragment length cannot be empirically derived from read mapping and is assumed to follow a truncated Gaussian distribution with user-specified mean and standard deviation. For the single-end libraries, we considered the mean fragment length to be 1.2 × the median read length and the standard deviation to be 0.1 × the mean fragment length. Varying these parameters did not substantially alter the expression profile of each sample.

The selected BioProjects include subsets of resident tissue macrophages defined using surface markers or reporter genes as indicated in Table 1 and separated by FACS, as well as temporal profiles of adaptation from monocytes to tissue macrophages. Several studies involve the analysis of the impact of mutations in specific transcription factors and surface receptors. The focus is on tissue-resident cells. Data sets related to inflammatory macrophages or macrophages stimulated in vitro have been excluded.

The purpose of this analysis was to identify clusters of transcripts that are robustly correlated regardless of the tissue of origin. The outcomes of such an analysis may reveal tissue-specific modules but may equally include modules that are shared by several tissues or specific niches within tissues. For this purpose, the size of the data set and the diversity of transcriptomic space sampled is a major strength.

In an RNA-seq library, the distribution of TPM estimates should comply, to a reasonable approximation, with Zipf’s law, which states that the probability of an observation is inversely proportional to its rank [71, 72]. We confirmed that each of the 466 libraries obeyed the predicted power-law relationship.

Prior to network analysis, transcripts that were not detected at an arbitrary threshold of 10 TPM in at least 1 sample were removed to further minimise stochastic sampling noise intrinsic in RNA-seq data. Given the nature of the samples, this also helps to reduce the low-level representation of transcripts derived from contaminating cells of nonmyeloid origin. Of the 18,175 genes that met this minimum threshold, 11,578 were detected in at least 90% of the RNA-seq data sets, and 6,901 had a median expression >10 TPM across the 466 samples. The TPM estimates for the 18,175 genes quantified in all 466 samples included are provided in S1 Data.

Network analysis was performed using the program BioLayout (http://biolayout.org). Pairwise Pearson correlations (*r*) were calculated between all samples to produce a sample-to-sample correlation matrix and inversely between all pairs of genes to produce a gene-to-gene correlation matrix. Gene coexpression networks (GCNs) were generated from the matrix, in which nodes represent genes and edges represent correlations between nodes above a defined correlation threshold. Note that BioLayout supports 3-dimensional visualisation of network graphs, and each of the graphs generated in this study can be regenerated using the data provided in Supporting Tables and the freely available software. For each gene-to-gene analysis, the value of *r* was chosen to generate an optimal network that retains the maximum number of transcripts (nodes) with the minimum number of edges [45]. Networks based on Spearman correlation coefficients (nonparametric) were also calculated to assess the impact of distribution shape on the analysis.

## Results and discussion

### Overview

The massive data set in S1 Data presents expression estimates for 18,175 transcripts in 466 RNA-seq data sets from MPS cells in a wide diversity of differentiated states. This provides the framework for a meta-analysis and review of the current state of knowledge of MPS cell differentiation. We first consider the expression profiles of individual transcripts, a network analysis of the relationships between MPS cell populations, and identification of stringently coregulated clusters of transcripts. This analysis leads to detailed consideration of the content of individual clusters enriched in tissue-specific MPS populations and the recognition of likely artefacts associated with isolation of MPS cells. Separate analysis of expression of transcripts encoding transcription factors addresses the control network that regulates differential gene expression in the MPS, whilst analysis of solute carriers and metabolic pathways contributes to the emerging field of immunometabolism. scRNA-seq is a rapidly emerging approach to identification of MPS subpopulations. Based upon comparative analysis, we critically review the validity of conclusions about MPS heterogeneity based upon scRNA-seq and the relationship with coregulated clusters identified by network analysis. In the light of our analysis, we question the validity of cell surface markers in analysis of MPS cell diversity, especially as they relate to the separate identity of DC and macrophage polarisation states.

### Expression profiles of individual transcripts

**Fig 1:**
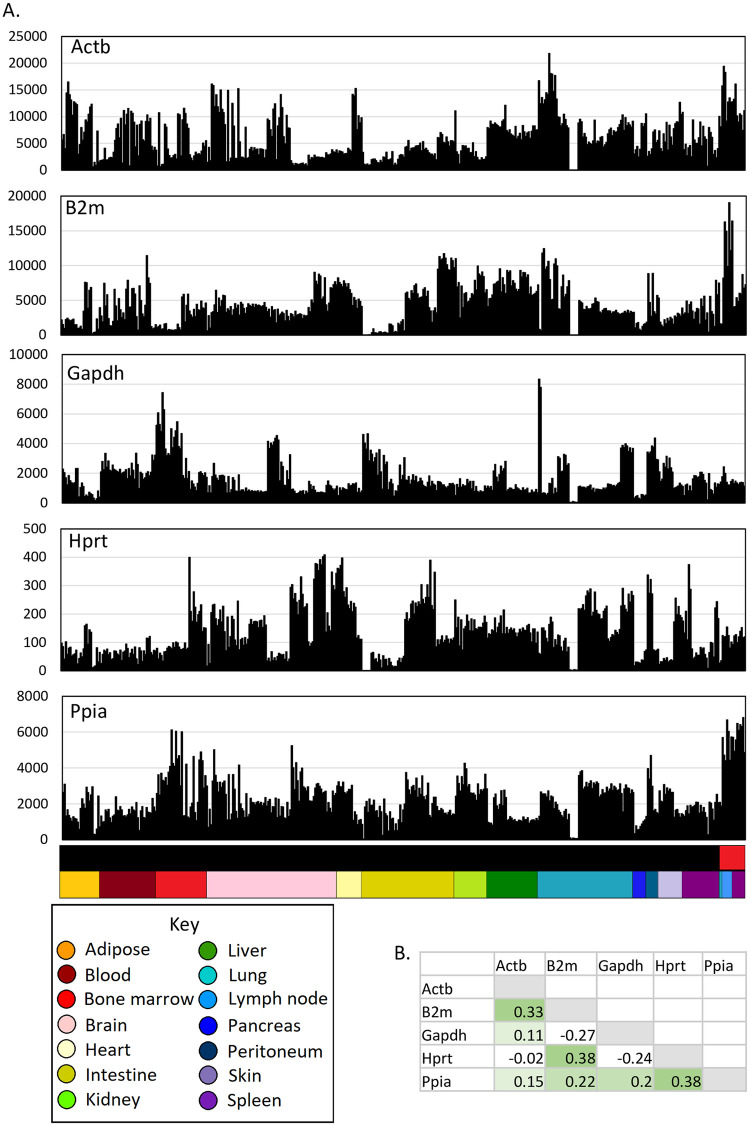
Expression of housekeeping genes across MPS cell populations. The data underlying this Figure can be found in S1 Data. (A) Expression patterns across cells from different tissues. Each column represents a sample. Upper bar along the *X* axis shows the cell type (black—monocytes and macrophages; red—DCs). Lower bar shows the tissue, coloured as shown in the key. *Y* axis shows expression level in TPM, calculated using Kallisto. (B) Correlations (Pearson correlation coefficient) between expression patterns of different housekeeping genes. DC, dendritic cell; MPS, mononuclear phagocyte system; TPM, transcripts per million.

To survey the heterogeneity of the MPS cells (monocytes, macrophages, and DCs), we first considered the expression profiles of selected individual transcripts, including candidate housekeeping genes and surface markers commonly used in studies of this lineage. The choice of appropriate reference genes for quantitative reverse transcriptase polymerase chain reaction (qRT-PCR) quantification of RNA levels is a significant issue in many studies, including macrophage differentiation. For example, Stephens and colleagues [73] proposed the use of a weighted geometric average of the most stably expressed genes for studies of differentiating macrophages and osteoclasts. Fig 1A shows the expression profiles of candidate housekeeping genes (*Hprt*, *Actb*, *B2m*, *Gapdh*, *Ppia*) commonly used in qRT-PCR as reference genes. We envisaged that these transcripts would be relatively consistent between data sets and would be correlated with each other. However, each of these transcripts varied by >10-fold among the MPS populations, and in pairwise analysis, they were only weakly correlated (Pearson correlations in Fig 1B and Spearman correlations in S1 Fig). S1 Data also includes the mean, standard deviation, and coefficient of variance (CoV) of each transcript across the entire data set. Only 200 transcripts had a CoV < 0.5. This analysis indicates that pathways normally considered as housekeeping (intermediary metabolism, protein synthesis, endoplasmic reticulum (ER) and Golgi membrane trafficking and secretion, endocytosis, cytoskeleton, etc.) are independently regulated in MPS cells. There are few, if any, identifiable housekeeping genes.

**Fig 2:**
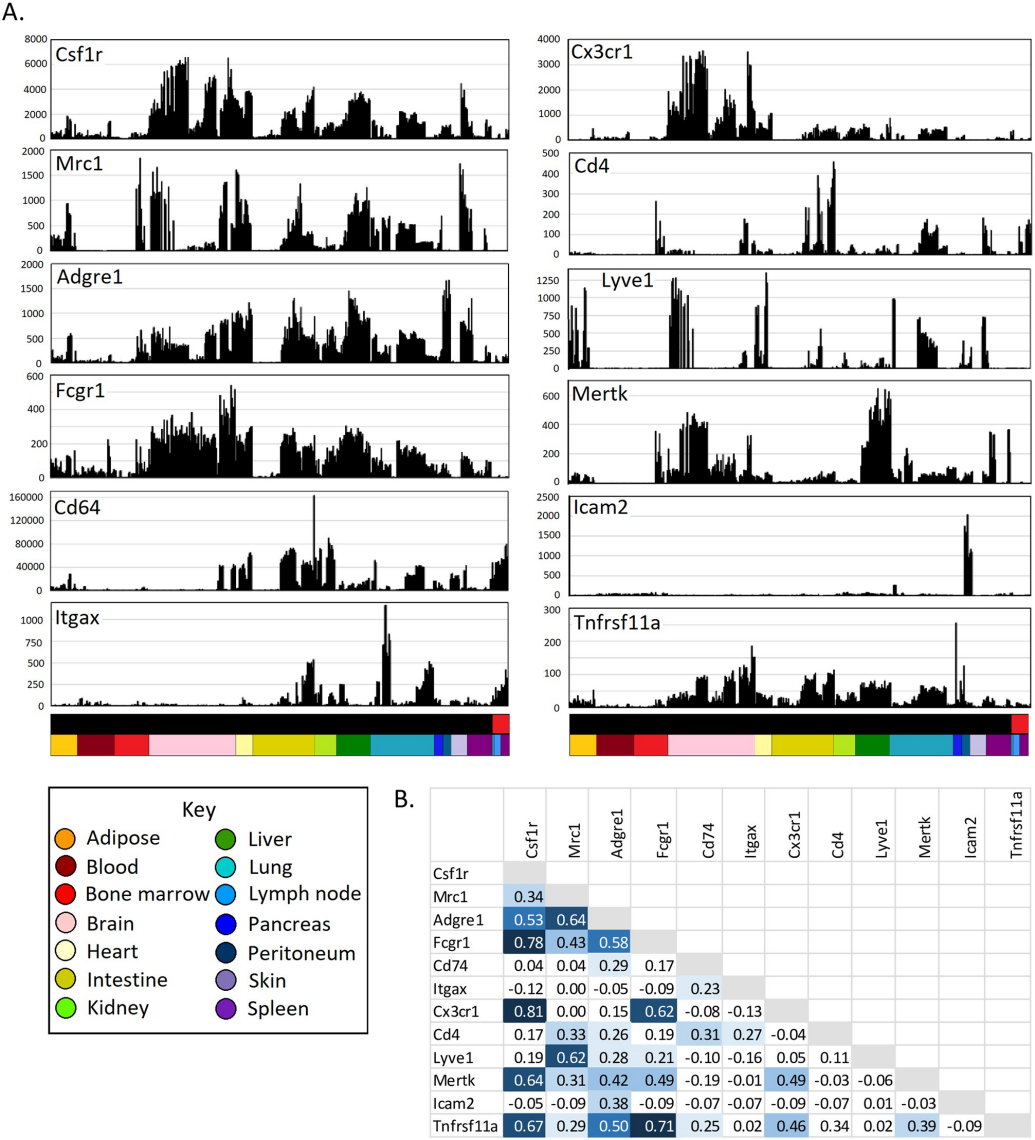
Expression of cell surface marker genes across MPS populations. The data underlying this Figure can be found in S1 Data. (A) Expression patterns across cells from different tissues. Each column represents a sample. Upper bar along the *X* axis shows the cell type (black—monocytes and macrophages; red—DCs). Lower bar shows the tissue, coloured as shown in the key. *Y* axis shows expression level in TPM, calculated using Kallisto. (B) Correlations (Pearson correlation coefficient) between expression patterns of different MPS genes. DC, dendritic cell; MPS, mononuclear phagocyte system; TPM, transcripts per million.

Fig 2A shows the expression pattern of transcripts encoding surface markers used to separate some of the subpopulations herein: *Adgre1* (encoding F4/80), *Cd4*, *Cd74* (Class II MHC), *Csf1r* (CD115), *Cx3cr1*, *Fcgr1* (CD64), *Icam2*, *Itgax* (CD11C), *Lyve1*, *Mertk*, *Mrc1* (CD206), and *Tnfrsf11a* (RANK). Fig 2B shows a summary of the Pearson correlations between them; Spearman correlations are shown in S1 Fig. Consistent with studies using *Csf1r* reporter transgenes [74, 75], *Csf1r* mRNA was universally expressed in MPS cells, albeit with significant variation in level, being highest in microglia and lowest in cDC1s. *Csf1r* was correlated (Pearson *r* > 0.5, Spearman *r* > 0.65) with *Adgre1*, *Fcgr1*, *Cx3cr1*, *Mertk*, and *Tnfrsf11a*, but these transcripts were less correlated with each other. *Mrc1* was reported to be correlated with expression of *Lyve1* and inversely with Class II MHC transcripts [27, 76]. Across the entire spectrum of macrophage transcriptomes, *Mrc1* was correlated with *Lyve1* but was more widely expressed (Fig 2A). However, there was no evidence of an inverse correlation between *Mrc1* and *Cd74* or other Class II MHC-associated transcripts.

### Network analysis of relationships of MPS populations and expressed transcripts

**Fig 3:**
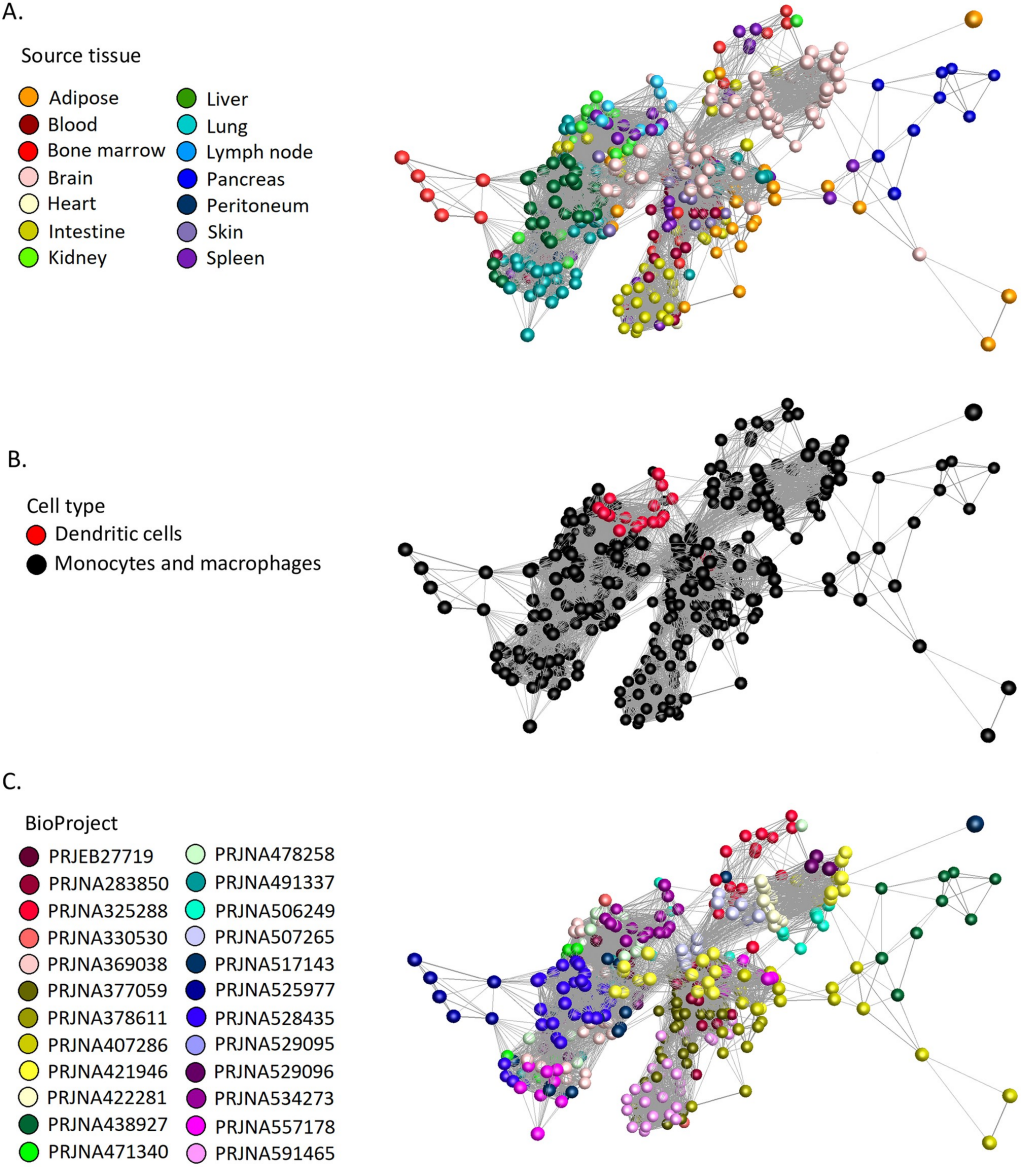
Sample-to-sample network analysis of gene expression in MPS cell populations. Each sphere (node) represents a sample, and lines between them (edges) show Pearson correlations between them of ≥0.68 (the maximum value that included all 466 samples). (A) Samples coloured by tissue of origin. (B) Samples coloured by cell type. (C) Samples coloured by BioProject. MPS, mononuclear phagocyte system.

To determine whether any transcripts encoding surface markers were correlated with cellular phenotype, we used the graph-based network analysis tool BioLayout. Fig 3 presents a sample-to-sample correlation matrix generated using the Fruchterman–Rheingold algorithm in BioLayout showing the clear segregation of the MPS populations based on the tissues from which they were isolated (Fig 3A).

Initial iterations of the sample-to-sample analysis identified 3 technical issues that served to validate our approach and data quality. We initially included data from the large ImmGen UL1 project (GSE127267l; GSE124829; see [77]), but this project uses an ultra-low–input RNA-seq pipeline based upon 1,000 sorted cells and the scRNA-seq platform Smartseq2. The initial sample-to-sample analysis revealed a large batch effect for these samples relative to all other samples, and we therefore excluded these data. Secondly, we mistakenly included populations of stellate cells, endothelial cells (ECs), and hepatocytes profiled within a study of the influence of the hepatic niche on Kupffer cell (KC) development [78]. The replicate samples of these non-MPS cells formed 3 entirely separate clusters each distinct from the main MPS cluster, and these were also excluded. Thirdly, the initial analysis indicated that macrophages isolated from the choroid plexus clustered with resident peritoneal macrophages. Consultation with the authors of the study [59] confirmed that this was due to a 3-way error in upload to the SRA, and the samples labelled choroid plexus macrophages were indeed resident peritoneal macrophages; those labelled peritoneal macrophages were actually microglia, and those labelled microglia were choroid plexus macrophages. The discovery of these technical issues highlights the power and validity of the network approach as implemented in BioLayout.

Consistent with previous analysis of microarray data sets, in which all MPS cells, including blood monocytes and DCs, clustered together and were clearly distinct from other haematopoietic and nonhaematopoietic lineages [39, 41, 42, 44], the isolated spleen, lung, and lymph node (LN) DC subpopulations did not form a separate element in the network (red nodes in Fig 3B). Based upon their overall transcriptomic profile, the DCs were no more divergent from other MPS populations than the isolated monocytes and macrophages purified from different tissues were from each other. The apparent relationship to BioProject (Fig 3C) occurs mainly because most studies were focussed on a particular tissue or cell type. There may also be minor impacts from differing methods of extracting and processing RNA and low-depth and single-end libraries compared with high-depth/paired-end libraries, but nonetheless, when different groups had profiled the same cell populations, the profiles were clustered together.

**Fig 4:**
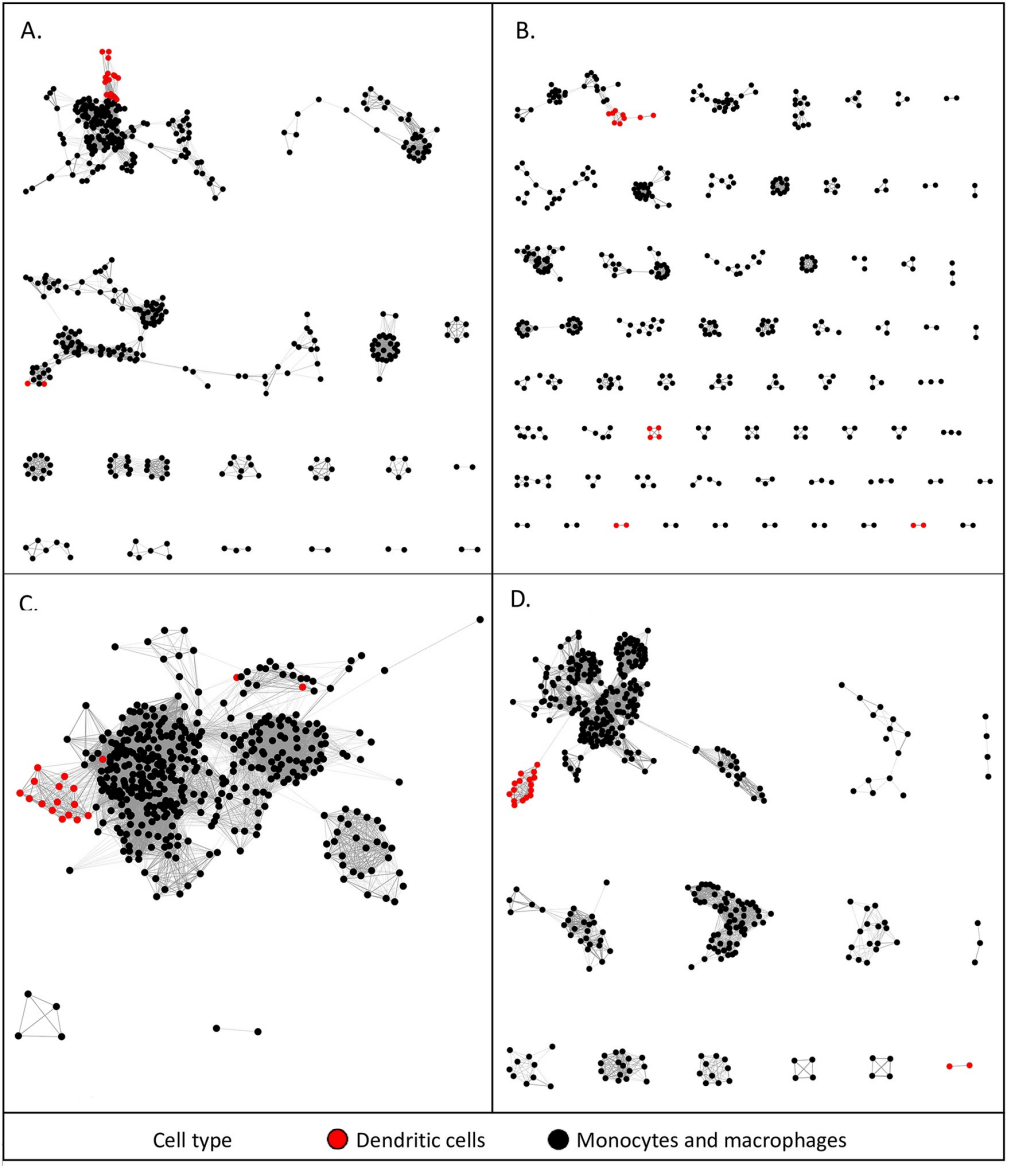
Sample-to-sample 2D network analysis of gene expression in monocyte, macrophage, and DC populations. Each sphere (node) represents a sample, and lines between them (edges) show Pearson correlations between them. (A) Network laid out at Pearson correlation coefficient of ≥0.85. The network includes 458 samples. (B) Network laid out at Pearson correlation coefficient of ≥0.95. The network includes 418 samples. (C) Network laid out at Spearman correlation coefficient of ≥0.85. The network includes 443 samples. (D) Network laid out at Spearman correlation coefficient of ≥0.9. The network includes 427 samples. The networks with nodes coloured by tissue and BioProject are shown in S2–S5 Figs. DC, dendritic cell.

To further test the robustness of the associations between different MPS populations, we repeated the analysis with increasing *r* values of the Pearson correlation and based upon a Spearman correlation. Fig 4 presents the networks with nodes coloured by cell type at different Pearson and Spearman correlation coefficients. The networks with nodes coloured by tissue type and BioProject are presented in S2–S5 Figs. These networks clearly show that the DCs are more similar to monocytes and macrophages from the same tissue than they are to DCs from another tissue. Creating the network of samples at a Pearson *r* value of 0.85 clearly separated the microglia from the main network as the most divergent MPS cells (S2 Fig). Interestingly, the lung DCs were also separated from the lymphoid tissue DCs. At an *r* value of 0.95, the different MPS cells formed separate elements in the network (S3 Fig), but the cDC2 samples were still in the same element as macrophages from several nonlymphoid tissue sources. The close relationship of DCs with other MPS cells was equally evident when based upon Spearman correlation at the *r* threshold of 0.85 (S4 Fig) and at higher *r* (S5 Fig).

**Fig 5:**
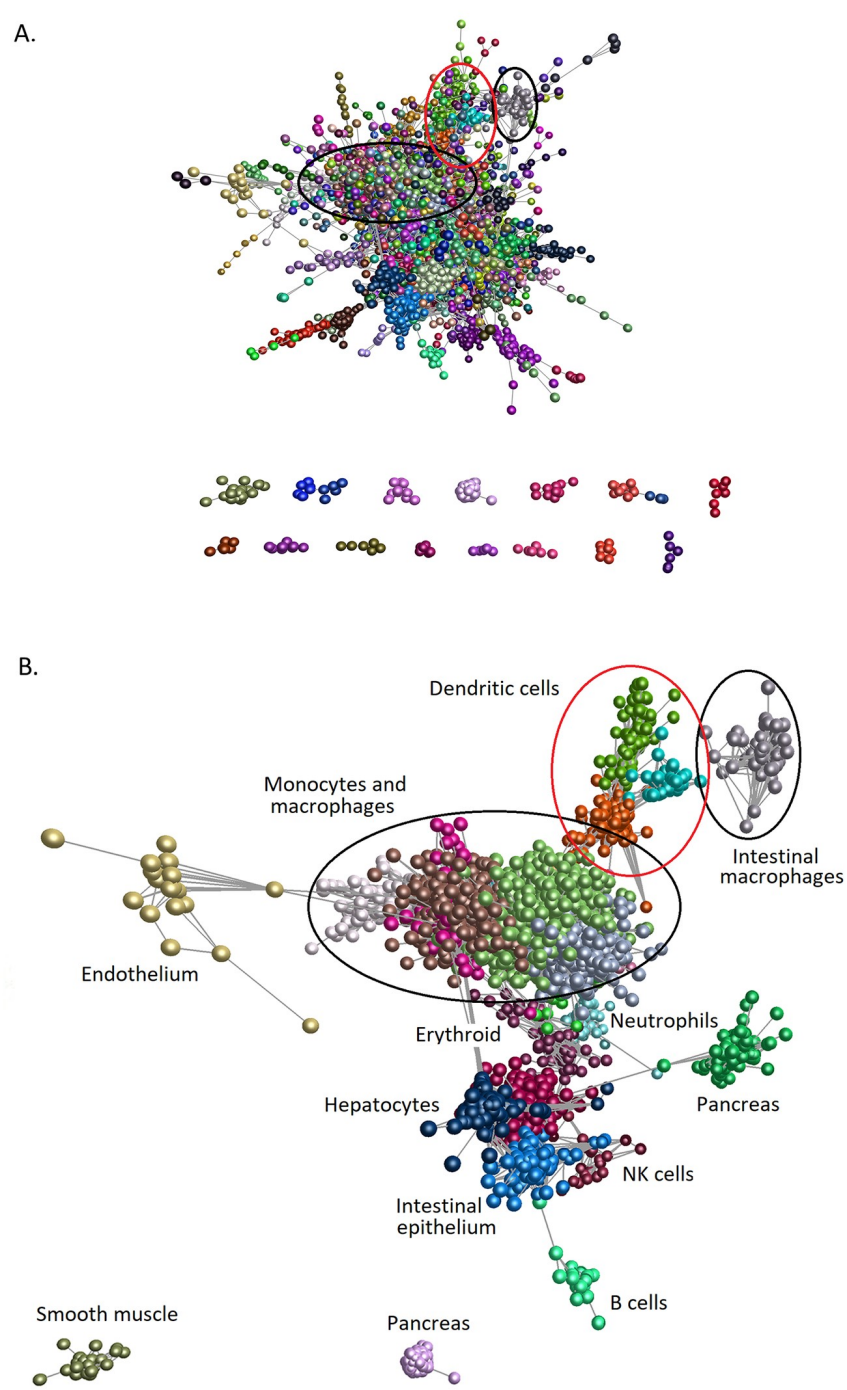
GCN analysis of gene expression in MPS cell populations. Each sphere (node) represents a gene, and lines between them (edges) show Pearson correlations between them of ≥0.75. Nodes were grouped into clusters with related expression patterns using the MCL algorithm with an inflation value of 1.7. Lists of genes and expression profiles of clusters are presented in S2 Data. (A) The network generated by the BioLayout analysis. Elements with ≥5 nodes are shown. Nodes are coloured by MCL cluster. Lists of genes and average expression profiles for all clusters are presented in S2 Data. Monocyte and macrophage genes (black ovals), DC genes (red oval). (B) Network showing only major clusters of monocyte and macrophage genes (black ovals), DC genes (red oval), and other cell types. DC, dendritic cell; GCN, gene coexpression network; MCL, Markov clustering algorithm; MPS, mononuclear phagocyte system; NK cell, natural killer cell.

The GCN for the same data set was developed at an optimal Pearson *r* value of 0.75 chosen based on the graph of network size versus correlation threshold (shown in S6 Fig). Fig 5A shows the whole network, and Fig 5B highlights the tissue-specific clusters and those that contain markers of other cell types, as discussed below. S2 Data summarises the coexpressed gene clusters and the average gene expression profiles of the clusters containing at least 10 nodes (transcripts). The graphs are colour-coded to indicate the tissue origin and cell type as in Fig 1 (samples are listed in the Readme sheet of S2 Data). An additional sheet in S2 Data provides gene ontology (GO) term enrichment of the larger clusters. For ease of visualisation relative to sample information, profiles of surface markers and transcription factors discussed below are provided as an additional sheet in S1 Data. Table 2 provides an overview of the major functional clusters discussed in more detail below. It is beyond the scope of this study to analyse and cite published evidence related to every transcript in detail. In Table 2, individual genes from within the cluster have been included based their candidate role as transcriptional regulators and upon known associations with mononuclear phagocyte biology determined by PubMed search on ‘Genename’ AND ‘macrophage’ or ‘dendritic cell’. On the principal of guilt by association [38–42], there are hundreds of other genes within these clusters that have inferred functions in innate immunity and mononuclear phagocyte biology.

**Table 2:** Description of major functional clusters of coexpressed genes in mouse MPS cell samples.

Cluster Number	Description	Representative Genes
1	MPS	*Acp2*, *Atp6* subunits, *Cd276*, *Cd53*, *Cd68*, *Cd84*, *Clec5a*, *Cln5/8*, *Csf1r*, *Ddx*/*Dhx* family, *Fcgr1*, *Gpr107/108*, *Hk3*, lysosomal enzymes, *Ifngr1/2*, *Il10ra*, *Il13ra1*, *Il6ra*, *Irak1/2*, *Jak1/3*, *Lamp1/2*, *Lgals8/9*, *M6pr*, *P2ry6*, *P2rx7*, *Sirpa*, *Tlr6/7/8*, *Tnfrsf11a*, ***Cebpg***, ***Creb3***, ***Crebzf***, ***Elf1***, ***Etv5***, ***Fli1***, ***Foxj2***, ***Foxn3***, ***Foxo1***, ***Gabpa***, ***Hdac3/10***, ***Hif1a***, ***Hsf1***, ***Klf3***, ***Maf1***, ***Mafg***, ***Mitf***, ***Nfatc1***, ***Nfx1***, ***Nfyc***, ***Nr1h2***, ***Nr2c1***, ***Nr2f6***, ***Nr3c1***, ***Prdm4***, ***Rela***, ***Smad1/2/4***, ***Sp3***, ***Spi1***, ***Srebf1***, ***Stat6***, ***Tcf3***, ***Tfe3***
3	MPS	*Abca1/2*, *Aim2*, *Akt2/3*, *Arrb1*, *Arrb2*, *Atxn7*, *Bak1*, *Cbl*, *Cd180*, *Cdk8/10/12/13/19*, *Csk*, *Ddi2*, *Ddx3/6/17/19a/21/23/39b/46*, *Dhx9/15*, *Grk2*, *H6pd*, *Ly9*, *Megf8*, *Mertk*^a^, *Mpeg1*, *Naip2/5/6*, *Nirp1b*, *Ptprj*, *Socs4/7*, *Syk*, *Taok1/2*, *Traf7*, *Tram2*, ***Atf1***, ***Bach1***, ***Bcor***, ***Cebpa***, ***Elf2/4***, ***Erf***, ***Foxk1***, ***Foxk2***, ***Foxo3***, ***Foxo4***, ***Fus***, ***Hsf2***, ***Ikzf1***, ***Maf***, ***Maz***, ***Mef2d***, ***Ncoa3***, ***Ncoa6***, ***Nfat5***, ***Nfatc3***, ***Nfya***, ***Pbx2***, ***Prdm2***, ***Smad5***
4	Microglia and brain macrophages	*Abi3*, *Acvr1*, *Adrb2*, *Bcl9*, *Bmp1/2k*, *Card6*, *Ccr5*, *Cd34*, *Csf3r*, *Cx3cr1*, *Cxxc5*, *Ddx31/43*, *Entpd1*, *Fcrls*, *Fgf13*, *Gabbr1*, *Gpr155*, *Gpr165*, *Gpr34*, *Hexb*, *Itgb3/b5*, *Lpcat1/2/3*, *Mrc2*, *Nckap5l*, *Olfml3*, *P2ry12/13*, *Paqr7*, *Plexna4*, *Nanos1*, *Siglech*, *Slc1a3/4*, *Slco2b1*, *Slc2a5*, *Sipa1*, *Tgfbr1*, *Tmem119*, *Tmem173*, *Trem2*, *Vav1*, *Vsir*, ***Bhlhb9***, ***Ebf3***, ***Elk3***, ***Ets1***, ***Hivep3***, ***Lefty1***, ***Mef2c***, ***Prox2***, ***Sall1/2/3***, ***Sox4***
7	Mitochondria and ribosome	*Atp5e/g2/h/j2/l*, *Cox5b/6a1/6b1*, *Mrpl* family, *Nduf* family, *Rpl* and *Rps* families
9	Cell cycle	*Aurka*, *Aurkb*, *Birc5*, *Bub1*, *Ccna2/b1/b2/e2*, *Cdk1*, *Cenpe*, *Haus* family, *Kif* family, *Mcm* family, *Plk1*, ***Foxm1***, ***Mybl2***
10	Lung macrophages	*Anxa2*, *Atxn10*, *Car4*, *Cd2*, *Cd200r4*, *Cd9*, *Chil3*, *Ctsk*, *Cx3Cl1*, *Cxcr1*, *F7*, *Fabp1*, *Ffar4*, *Flt1*, *Flvcr2*, *Gal*, *Htr2c*, *Igflr1*, *Il1rn*, *Lpl*, *Ly75*, *Nceh1*, *P2rx5*, *Plscr1*, *Serpine1*, *Siglecf*, *Slc6a4*, *Tmem138*, ***Nlrx1***, ***Pparg***, ***Tcf7l2***
12	Liver KCs, peritoneal and splenic red pulp macrophages	*Acp5*, *Adgre4*, *Apoc1*, *C6*, *Cd5l*, *Cdh5*, *Clec1b*, *Clec4f*, *Fabp7*, *Fcgr4*, *Il18bp*, *Itga9*, *Kcna2*, *Lrp5*, *Ly9*, *Pecam1*, *Pira1*/2, *Ptger1*, *Ptprj*, *Scarb1*, *Scarf1*, *Sema6d*, *Siglec1*, *Siglece*, *Slc11a1*, *Slc40a1*, *Slc1a2*, *Stab2*, *Tmem65*, *Treml4*, *Trpm2*, *Vsig4*, ***Elk1***, ***Id3***, ***Nr1h3***, ***Rxra***, ***Smad6***, ***Thrb***, ***Zbtb4***
13	CCR7 DCs	*Arc*, *Birc2*, *Cacnb3*, *Cblb*, *Ccl19*, *Ccl22*, *Ccr7*, *Cd1d1*, *Cd200*, *Cd40*, *Cd70*, *Dpp4*, *Fas*, *Icosl*, *Glipr2*, *Gpr68*, *Heatr9*, *H2-Q6/7/8/9*, *Il15*, *Il15ra*, *Itgb8*, *Laptm4b*, *Lrrk1*, *Slamf1*, *Socs2*, *Tank*, *Tmem19*, *Tnfrsf4*, *Tnfrsf9*, *Traf1*, *Tyk2*, *Vsig10*, *Zc3h12c*, *Zmynd15*, ***Foxh1***, ***Id2***, ***Ikzf4***, ***Spib***, ***Stat4***
15	Monocytes	*C3*, *Camkk2*, *Ccr2*, *Cd177*, *Cd244a*, *Celsr3*, *Clec2g*, *Erbb4*, *Fgr*, *Gpr15*, *Gpr35*, *Gpr141*, *Hpse*, *Il17ra*, *Itga4*, *Met*, *Mmp8*, *Ms4a4c*, *Nlrc5*, *Ptgir*, *Ptprc*, *Sell*, *Sgms2*, *Slk*, *Vcan*, ***E2f2***, ***Foxn2***, ***Jarid2***, ***Rara***, ***Rfx2***, ***Stat2***^b^
21	Peritoneal macrophages	*Ackr3*, *Alox15*, *Arg1*, *C4a/b*, *Car6*, *Cyp26a1*, *F5*, *F10*, *Fgfr1*, *Fzd1*, *Icam2*, *Itga6*, *Itgam*, *Jag1*, *Lbp*, *Lrg1*, *Mst1r*, *Naip1*, *Nt5e*, *Padi4*, *Pycard*, *Selp*, *SerpinB2*, *Slpi*, *Tgfb2*, *Thbs1*, *Wnt2*, ***Gata6***, ***Rarb***, ***Smad3***, ***Sox7***, ***Tox2***
22	LYVE1-positive macrophages	*Adam9*, *C3ar1*, *C5ar1*, *Cd36*, *Cfh*, *Clcn5*, *Ctsb*, *Dab2*, *Egfr*, *Epor*, *F13a1*, *Fcgrt*, *Frmd6*, *Gas6*, *Gpr160*, *Igfbp4*, *Lyve1*, *Mrc1*, *Nrp1*, *S1pr1/2*, *Tlr5*, *Tmem9*, *Trf*, *Trpv4*, ***Etv1***, ***Nfatc2***, ***Tcf4***
28	DCs	*Adam11*, *Bcl2a1b/d*, *Ccr6*, *Cd7*, *Clec4a4*, *Ddr1*, *Dtx1*, *Flt3*, *H2-DMb2*, *H2-Eb2*, *H2-Oa/b*, *Kit*, *Lta/b*, *Nlrp10*, *P2ry10*, *Siglecg*, *Sirpb1a*, *Tnfrsf18*, ***Relb***
38	Intestinal macrophages	*Adam19*, *Asb2*, *Cxcl9*, *Cxcr4*, *Dna1l3*, *Fgl2*, *Gpr31b*, *Gpr55*, *Il10*, *Il12rb1*, *Kynu*, *Mmp9/13/14*, *Ocstamp*, *P2rx6*, *Pgf*, *Tlr12*, *Wnt4*, ***Fosb***, ***Hes1***, ***Hic1***
41	Immediate early genes	*Ccrl2*, *Dusp1*, *Mcl1*, *Tnfaip3*, *Trib1*, *Zfp36*, ***Atf3***, ***Egr1***, ***Fos***, ***Ier2/5***, ***Jun***, ***Junb***, ***Jund***, ***Klf2***, ***Klf6***, ***Nfe2l2***, ***Nfkbiz***, ***Tgif1***
43	LCs	*Cd207*, *Dkk1*, *Dpep3*, *Hapin3*, *Il1r2*, *Mfge8*, *P2rx2*, *P2rx5*, *Plek2*, *Sema7a*, *Serpind1*, *Tnfaip2*
49	cDC1s	*Cd8a*, *Clec4b2*, *Clnk*, *Ctla4*, *Gcsam*, *Gpr33*, *Gpr141b*, *Gpr171*, *Ildr1*, *Itgae*, *Il12b*, *P2ry14*, *Procr*, *Plekha5*, *Tlr11*, *Xcr1*, ***Ncoa7***
165	Class II MHC	*Cd74*, *H2-Aa*, *H2Ab1*, *H2-DMa/b1*, *H2-Eb1*

Bold text indicates transcription factors. For descriptions and accession numbers of all genes, please see S1 Data.

**Abbreviations:** cDC, classical DC; DC, dendritic cell; KC, Kupffer cell; LC, Langerhans cell; MHC, major histocompatibility complex; MPS, mononuclear phagocyte system.

### Major macrophage-enriched coregulated clusters

At the chosen *r* threshold of 0.75, the GCN approach using the normalised data from multiple laboratories identified many coregulated clusters of transcripts that are known to be functional in MPS cells based upon prior knowledge inferred from smaller data sets. In the large transcriptional atlas projects across many species and tissues discussed in the introduction, the largest clusters tend to contain housekeeping genes that show relatively little variation amongst tissues. Consistent with the analysis of individual housekeeping genes in Fig 1 and analysis of the variance for the entire data set in S1 Data, there is no such cluster in the MPS data set.

Cluster 1 is a generic MPS cluster that drives the relatively close association between all of the samples, including the different subclasses of DCs, in the sample-to-sample network (Figs 3B and 4) and distinguishes MPS cells from other leukocytes. It includes *Csf1r*, *Fcgr1*, *Cd68*, *Sirpa*, *Tnfrsf11a* and the core myeloid transcription factor gene *Spi1* alongside many other known MPS-enriched transcription factors [79, 80]. One notable inclusion is the glucocorticoid receptor gene, *Nr3c1*, which mediates transcriptional activation of a wide range of anti-inflammatory genes in macrophages [81]. As one might expect from the known endocytic and secretory activity of MPS cells, the cluster is enriched for GO terms related to endosome/lysosome and intracellular transport/secretion, which are major constitutive functions of mononuclear phagocytes [38] (S2 Data). Transcripts in Cluster 3 were also expressed widely in MPS cells, but the cluster has a distinct average expression profile. Cluster 3 includes genes encoding several forkhead transcription factors (*Foxo3*, *Foxo4*, *Foxk1*, and *Foxk2*), the key transcriptional regulators of autophagy [82–84], and *Nfat5*, which controls macrophage apoptosis [85]. This cluster also contains *Mertk*, the perforin-like immune effector gene *Mpeg1*, *Aim2* (which encodes a sensor for cytoplasmic DNA [86]), and transcripts for numerous DEAH- and DEAD- box helicases all implicated in DNA sensing in innate immunity [87]. There are also members of the neuronal apoptosis inhibitory protein (NAIP) family of inflammasome regulators (*Naip2*, *5*, *6*); reviewed in [88]). We infer that this cluster of transcripts reflects an independently regulated capacity for innate immune recognition of internalised pathogens. Other than *Mertk*, there is no other plasma membrane marker associated with this set of candidate innate immune effector genes.

Genes in Cluster 4 were strongly expressed in samples from brain and include microglia-enriched markers that are depleted in brains of *Csf1r*-deficient mice and rats, such as *Cx3cr1*, *Tmem119*, *P2ry12*, and the key transcription factor genes *Sall1*, *Sall2*, and *Sall3* [17, 89]. This cluster drives the separation of microglia as the most distinctive member of the MPS family. Cluster 9 contains the S phase transcription factor gene *Foxm1* and numerous cell-cycle–associated transcripts [90], and the GO term enrichment supports a cell-cycle role. Genes of the cell-cycle cluster were expressed in all isolated MPS populations at various levels, consistent with evidence that they are capable of self-renewal in the steady state [5, 6, 13]. The separation of this cluster indicates that proliferative activity is not tightly linked to any MPS differentiation state or surface marker.

### Identification of a capillary-associated expression cluster

Most macrophages and DCs included in this analysis were purified by FACS based upon their expression of specific markers including those shown in Fig 2 (see Table 1). Chakarov and colleagues [27] identified a population of pericapillary cells in the lung that expressed LYVE1 and extended their analysis to FACS-separated cells from fat, heart, and dermis. Their RNA-seq results are included in our data set. Based upon analysis of differentially expressed genes, the authors identified a set of genes with high expression in sorted LYVE1^hi^ macrophages relative to LYVE1^lo^ macrophages across the 4 tissues, including *Mrc1*, *Timd4*, *Cd5l*, *Fcna*, and *Vsig4* [27, 76]. The GCN reveals that there is, indeed, a set of transcripts (Cluster 22; see S2 Data) that is strongly correlated with *Lyve1* expression across MPS cells from a larger spectrum of tissues. The cluster includes *Mrc1* but excludes *Timd4*, *Cd5l*, *Fcna*, and *Vsig4*, which were associated with distinct tissue-specific clusters (Table 2). The correlation between *Lyve1* and *Mrc1* is actually lower than the network threshold of 0.75 (*r* = 0.62, Fig 2B). The 2 genes were included in Cluster 22 because of shared links to other genes. In fact, *Mrc1* was only marginally enriched in the purified LYVE1^hi^ macrophages from fat, heart, lung, and skin [27]. It was highly expressed in isolated MPS cells from adipose, brain, intestine, kidney, and liver that lack *Lyve1* mRNA (see S1 Data/selected transcripts and Fig 2). We conclude that most LYVE1^hi^ macrophages express *Mrc1*, but the reciprocal relationship does not hold.

The set of coexpressed genes in Cluster 22 suggests a function for LYVE1^hi^ macrophages in control of endothelial biology and vascular permeability. It includes genes for 2 of the sphingosine-1-phosphate (S1P) receptors (*S1pr1* and *S1pr2*) that have been implicated in many aspects of inflammation, lymphangiogenesis, and angiogenesis [91, 92]; the vanilloid receptor (*Trpv4*), which controls capillary barrier function and inflammation [93, 94]; and neuropilin 1 (*Nrp1*), which controls endothelial homeostasis [95]. Cluster 22 also contains the erythropoietin receptor gene (*Epor*), which was shown to synergise with S1P to promote apoptotic cell clearance by macrophages [96], and the epidermal growth factor (EGF) receptor gene (*Egfr*), which has also been shown to regulate macrophage function in a range of inflammatory models [97]. Indeed, the coexpressed genes might support the known functional association of macrophages with lymphatic as well as blood vessels [98]. The *Lyve1*-associated cluster contains genes for 3 candidate transcriptional regulators: *Etv1*, *Nfatc2*, and *Tcf4*. *Etv1* expression in macrophages has been implicated in functional polarisation in vitro and the response to altered mitochondrial membrane potential [99]. *NFATC2* is required for osteoclast differentiation in vitro [100], but its roles in macrophage differentiation/function have not been explored. *Tcf4* encodes a transcription effector of the Wnt/β-catenin pathway, which is implicated in responses to E-cadherin and other effectors in macrophage differentiation [101].

*Mrc1* is commonly referred to as a marker for alternative or M2 macrophage polarisation [31]. Another putative marker of M2 polarisation is the somatic growth factor insulin-like growth factor 1 (*Igf1* gene) [102]. *Igf1* was correlated with *Mrc1* (*r* = 0.67) but did not form part of a coexpression cluster. It was absent from monocytes and DCs but was highly expressed in most resident tissue macrophages (see S1 Data/selected transcripts). *Igf1* is CSF1-inducible and of particular interest because of the profound impact of *Csf1r* mutations in multiple species on postnatal growth and development [8]. Unlike hepatocytes and mesenchymal cells, tissue macrophages did not express transcripts encoding the growth hormone receptor (*Ghr*), *Igf1r*, or the *Igf1* binding protein genes (*Igfals*, *Igfbp1*, *2*, *3*, *5*, *6*). The exception is *Igfbp4*, which was highly expressed in most macrophage populations and did form part of the *Lyve1*/*Mrc1*-associated Cluster 22. Interestingly, *Igfbp4* knockout in mice mimics impacts of *Csf1r* deficiency on somatic growth and adipose formation [103, 104].

The intimate association of macrophages with capillaries was evident from the first localisation of the F4/80 antigen [105]. *Adgre1* expression was also correlated with *Mrc1* (*r* = 0.64; Fig 2B), but it was more widely expressed than either *Mrc1* or *Lyve1* and therefore not within Cluster 22. *Adgre1* was not enriched in any of the purified LYVE1^hi^ macrophage populations relative to LYVE1^lo^ cells from the same tissue [27]. It was high in most isolated tissue macrophages and induced during differentiation of monocytes in situ, as in the liver data set [32] and the intestinal developmental series [36, 37]. F4/80 (now known as ADGRE1) was proposed as a marker of macrophages of embryonic origin [106], but *Adgre1* mRNA was equally high in intestinal macrophages, which turn over rapidly from monocytes [107, 108], and in cDC2s. It was also strongly induced during monocyte differentiation to occupy a vacant KC niche [32]. Whatever the association with ontogeny, the pattern is rodent-specific. *Adgre1* is a rapidly evolving gene, and the expression pattern also varies across species [109].

### Tissue-specific macrophage clusters

Several coexpressed clusters were associated with MPS cells isolated from a single tissue. Aside from the large brain-enriched expression cluster (Cluster 4) that contains many microglia markers, Cluster 10 was lung-enriched and contains the alveolar macrophage marker *Siglecf* and key transcription factor *Pparg* [16]. Cluster 12 was shared amongst liver KCs, peritoneal macrophages, and splenic macrophages and includes the transcription factors *Id3*, *Nr1h3*, and *Smad6* and markers *Cd5l*, *Clec4f*, and *Vsig4* [16, 32, 37]. Within Cluster 12, we noted the strong coexpression (*r* = 0.81) between *Nr1h3* and *Rxra*, the gene encoding its promiscuous heterodimerisation partner, which is also implicated in control of KC lipid and iron metabolism [110] and may have independent function in innate immune regulation [111].

The average expression of Cluster 12 increased progressively in the monocyte-KC differentiation series [32] included in this data set (see profile in S7 Fig). Cluster 12 also reveals the regulated and coordinated expression of the thyroid hormone receptor (*Thrb* gene), likely mediating the many impacts of thyroid hormones in innate immune function [112]. One other potential regulator identified in this cluster is *Zbtb4*, which encodes an epigenetic regulator with a high affinity for methylated CpG. *Zbtb4*^−/−^ mice are viable and fertile but growth retarded compared with littermates [113]. Impacts on myeloid differentiation have not been reported. The transcription factor SPIC is implicated in splenic red pulp macrophage differentiation and iron homeostasis [114, 115]. Although *Spic* mRNA was highest in red pulp macrophages, KCs, and bone marrow macrophages, it was detected in other macrophage and DC populations and therefore has a unique expression profile. Cluster 21 contains transcripts most highly expressed in resident peritoneal macrophages and includes the genes for the transcription factor *Gata6* and the retinoic acid receptor (*Rarb*), which control peritoneal macrophage survival and adaptation [116, 117]. The data confirm the specific high expression of the enigmatic plasminogen activator inhibitor encoded by *Serpinb2* in resident peritoneal macrophages, first described >20 years ago [118] and still seeking a function [119].

Genes in Cluster 15, including the monocyte-specific chemotactic receptor *Ccr2*, were highly expressed in classical monocytes. Genes in Cluster 43 were expressed specifically in Langerhans cells (LCs). They include the marker *Cd207* (langerin) used in the purification of LCs [62] but also expressed at lower levels in many other tissue macrophage populations. This cluster did not include the gene for another LC marker, *Epcam* [62]. It was highly expressed in LCs but also detected in one set of intestinal macrophage samples, most likely a contamination with epithelial cells (Cluster 5, see below). Epidermal LCs have at times been considered as DC-like because of their migratory and APC properties but are now considered to be specialised resident tissue macrophages [120]. Unlike most classical DCs in lymphoid tissue but in common with nonlymphoid DCs, they are clearly CSF1R-dependent and share with several other macrophage populations dependence on the conserved enhancer in the *Csf1r* locus [17]. Cluster 43 did not include a transcriptional regulator specific to LCs. In common with several other macrophage populations, LC differentiation is regulated by transforming growth factor β (TGFβ) signalling, involving transcription factors RUNX3 and ID2 [120]. Both transcription factor genes were highly expressed in LCs but also present in several other tissue macrophage populations.

Intestinal macrophage-enriched gene expression profiles, which have not previously been identified, emerge in Cluster 38. Two large separate data sets of intestinal macrophages were included here [36, 37], both likely reflecting a differentiation series of adaptation from blood monocytes to resident intestinal tissue macrophages [5]. In one case, CD4 and TIM4 were used as markers [37]. Surprisingly, despite the fact that TIM4 was used as a marker to sort intestinal macrophages, the *Timd4* gene was not part of a significant coexpression cluster; it was highly expressed in the sorted intestinal macrophages but idiosyncratically in several other tissue macrophage populations. *Cd4* mRNA expression was shared uniquely with lung, skin, and kidney macrophage subpopulations (see Fig 2A). A third data set tracks the adaptation of transferred blood monocytes to the intestinal niche [69]. Cluster 38 identified *Cxcr4* as a candidate intestinal macrophage marker consistent with their continuous derivation from CXCR4^+^ monocytes. The high expression of *Wnt4* in lamina propria macrophages was recently confirmed by immunohistochemistry (IHC). Conditional deletion of *Wnt4* using *Itgax*-cre led to dysregulation of immunity against an intestinal parasite [121]. WNT4 is a candidate mediator of the key trophic role of lamina propria macrophages in the intestinal stem cell niche [122]. *Fosb*, *Hes1*, and *Hic1* encode identified potential transcriptional regulators of intestinal macrophage differentiation and adaptation. HES1 inhibits inflammatory responses in macrophages and contributes to gut homeostasis [123, 124]. FOSB has not previously been implicated in macrophage adaptation to any niche. Unfortunately, we were not able to include data from a microarray analysis of resident colonic macrophages that identified a set of 108 genes >2-fold higher in the colon relative to other macrophage populations in the ImmGen database [125]. However, Cluster 38 confirmed the gut-macrophage–specific expression of several of these transcripts, including *Dna1l3*, *Fgl2*, *Gpr31b*, *Hes1*, *Mmp13*, *Ocstamp*, *Pgf*, and *Tlr12*.

There were no unique expression profiles enriched in macrophages isolated from any other major tissues including adipose, brain (nonmicroglia), heart, kidney, pancreas, or skin. The abundant resident macrophages of adipose are especially topical in light of the obesity epidemic. The literature on adipose macrophages focusses on ‘M2-like’ markers [126]. Amongst resident macrophage populations, *Apoe* and *Retnla*, both detected in most tissue macrophages and not included in a cluster, were highest in adipose-derived macrophages. RETNLA (also known as RELMα) has been referred to as an adipokine, regulated by food intake and controlling lipid homeostasis [127]. Kumamoto and colleagues [128] claimed that *Retnla* was coexpressed with *Mgl2* (another putative M2 marker) in many mouse tissues, including adipose, and attributed it a role in maintenance of energy balance. The 2 transcripts were not correlated in this larger data set. In fact, *Mgl2* was part of a small cluster (Cluster 83) with *Cd14*. Like *Retnla*, mRNA for the related lectin, MGL1 (*Clec10a* gene)—also considered an M2 macrophage marker [126]—was highest in the adipose-associated macrophages but also expressed in macrophages from other tissues, including dura, heart, lung, and skin (Cluster 101).

### DC coexpression clusters

It has become a central dogma in immunology that DCs are uniquely adapted to present antigen to naïve T cells. This view has driven the search for surface markers to enable isolation of DCs for immunotherapy and receptors that can mediate selective antigen uptake to enhance immunisation. If the central dogma is correct and currently used DC markers have any validity, network analysis should uncover coexpression clusters associated with antigen uptake, processing, and presentation to T cells. This prediction is not supported by the data. Despite evidence that it is expressed by many resident tissue macrophages (reviewed in [25]), CD11C (encoded by *Itgax*) is still widely used as a surface marker in mouse DC purification. Ongoing studies of the impacts of conditional mutations using *Itgax*-cre continue to be interpreted solely in terms of DC specificity (for example, [121, 129, 130]). Consistent with the literature [25], *Itgax* was expressed in multiple macrophage populations other than DCs (Fig 2A) at levels at least as high as in DCs purified using CD11C as a marker and correlated only with *Cd22*, *Cd274* (encoding programmed cell death 1 ligand 1), *Csf2rb*, *Csf2rb2*, solute carrier (*Slc*)*15a3*, *Tmem132a*, and the transcription factor gene *Prdm1* (Cluster145). Class II MHC is also often used as a marker to purify DCs, and expression is obviously a prerequisite for antigen presentation to T cells. The ImmGen Consortium compared DCs from multiple sources with various macrophage populations to identify transcripts that distinguish DC from macrophages [28, 29]. Since the macrophages used for comparison were MHCII^lo^, the DC signature included class II MHC genes. In our meta-analysis, one small cluster (Cluster 165) contained the transcription factor gene *Ciita* and *Cd74*, *H2-Aa*, *H2Ab1*, *H2-DMa/b1*, and *H2-Eb1* encoding its targets. The genes in this cluster were clearly highly expressed in many tissue macrophages (see profile for *Cd74* in Fig 2A) but regulated independently of any other markers and expressed no higher in cells annotated as DCs than in cells annotated as macrophages from intestine, lung, heart, and kidney. Interestingly, again highlighting the issue with a definition of DCs based upon unique APC function, isolated lung MHCII^hi^ interstitial macrophages were as active as cDC2s in antigen-presentation assays in vitro [27]. These results are consistent with the sample-to-sample analyses that showed that DCs and monocytes/macrophages from the same tissue are more similar than DCs are to DCs from other tissues (Figs 3 and 4).

The GCN analysis did identify 3 separate DC-associated coexpression clusters that are consistent with current knowledge of putative DC subsets and adaptation in mice [21, 22, 131]. Cluster 13 includes *Ccr7* and transcription factor genes *Spib* and *Stat4*; Cluster 28 includes *Flt3*, *Kit*, and the transcription factor gene *Relb*; and Cluster 49 includes cDC1 markers *Itgae* (CD103) and *Xcr1*. CCR7 is associated with DC migration [132] and the transcript was abundant in both cDC1s and cDC2s isolated from spleen and LN. By contrast, the expression was much lower in isolated lung DCs and in kidney DCs from a separate data set (see below), similar to levels in isolated macrophages from multiple tissues. Several putative DC markers were excluded from DC-specific clusters. The transcription factor gene *Batf3*, implicated in cDC1 differentiation [133], did not form part of a cluster and was detected in most macrophage populations (consistent with [16]). Similarly, IRF4 has been attributed a specific function in cDC2 differentiation [130]. *Irf4* mRNA was more abundant in cDC2s than in cDC1s but was also expressed in monocytes and monocyte-derived macrophage populations. Transcripts encoding NFIL3 and IRF8, which interact in the regulation of cDC1 differentiation [134], were also highly expressed in cDC2s and in monocytes and many tissue macrophages. Although the transcription repressor gene *Zbtb46*, encoding a putative DC lineage marker [135], was highest in DCs, it was also detectable in most isolated tissue macrophages, notably in kidney and lung. Another putative DC marker gene, *Clec9a* [70], also clustered independently because of expression in isolated intestine, kidney, liver, and lung macrophages.

Interestingly, tissue macrophages may contribute to homeostatic regulation of cDC differentiation. The transcript encoding the FLT3 ligand (*Flt3l*) was expressed constitutively to varying degrees in all of the MPS populations studied. Fujita and colleagues [129] showed that FLT3L is cleaved from the cell surface of expressing cells by ADAM10. Conditional deletion of *Adam10* using *Itgax*-cre led to reduced differentiation of cDC2s. *Adam10* is also expressed by CD11C^+^ macrophages; it forms part of Cluster 3, containing genes that are low in monocytes and expressed by all resident macrophages at higher levels than in DCs.

**Fig 6:**
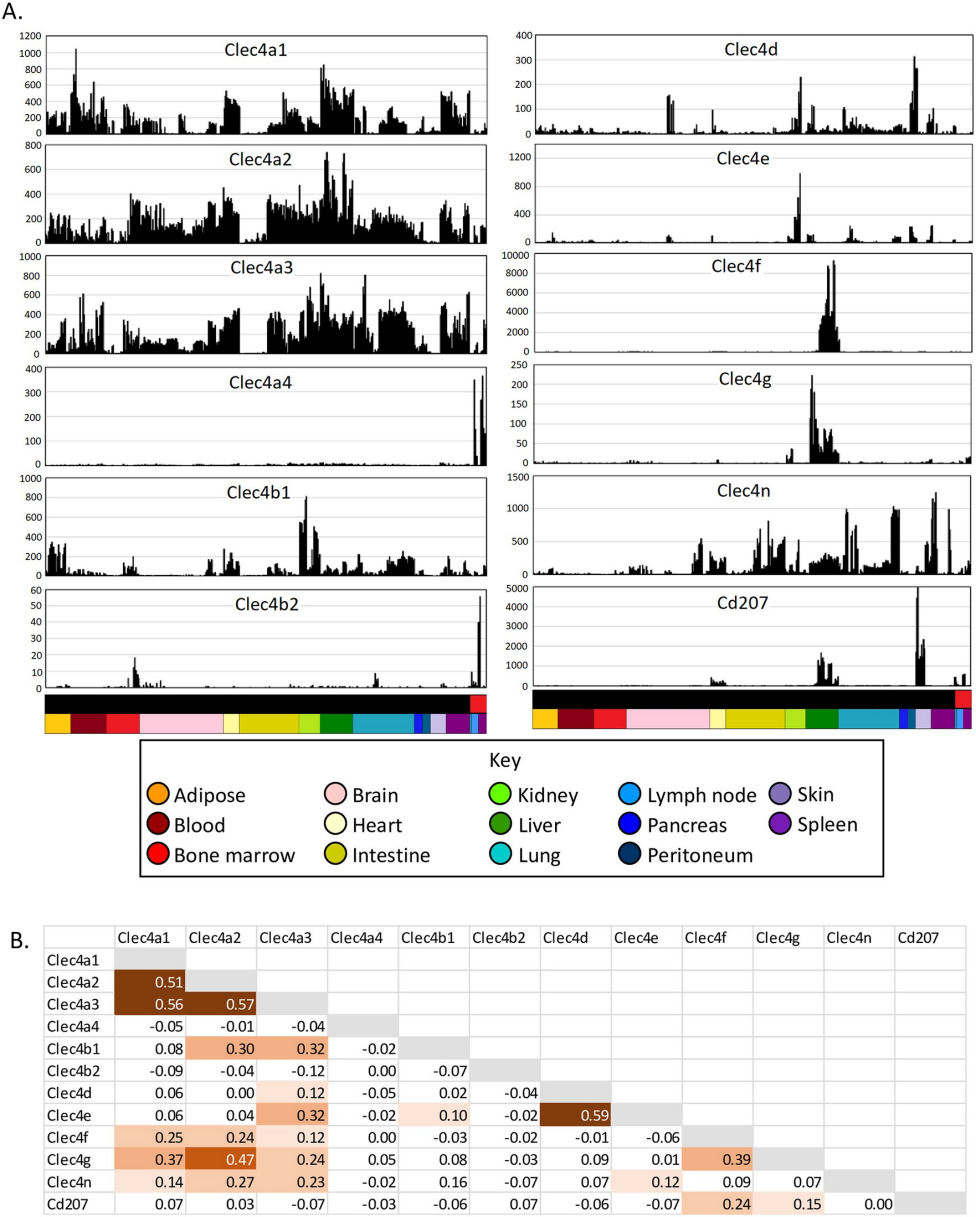
Expression of members of the DCIR (CLEC4) family across MPS cell populations. (A) Expression patterns across cells from different tissues. Each column represents a sample. Upper bar along the *X* axis shows the cell type (black—monocytes and macrophages; red—DCs). Lower bar shows the tissue, coloured as shown in the key. *Y* axis shows expression level in TPM, calculated using Kallisto. (B) Correlations (Pearson correlation coefficient) between expression patterns of different Clec4 genes. DC, dendritic cell; DCIR, DC immunoreceptor; MPS, mononuclear phagocyte system; TPM, transcripts per million.

Aside from CLEC9A, many other lectin-like receptors have been proposed as DC markers and inferred to have a function in antigen uptake. Fig 6 shows the profiles for the 12 members of the so-called DC immunoreceptor (DCIR) family. The original member of this family, *Clec4a2*, the likely ortholog of the single *CLEC4A* gene in humans, encodes a lectin with a broad binding specificity for mannose and fucose [136]. Studies on knockout mice lacking *Clec4a2* continue to be based upon the claim that the lectin is mainly expressed by DCs [137], but the global analysis showed that the mRNA is more highly expressed in most isolated macrophage populations. Two of the DC-associated clusters contained other members of the family, *Clec4a4* and *Clec4b2*. *Clec4a4* has been attributed a specific role in cDC1 function [138], but it was equally expressed in cDC2s and forms part of Cluster 28. Most of the *Clec4* genes in the mouse genome are in a single location on Chromosome 6. They also include macrophage-inducible C-type lectin (Mincle) encoded by *Clec4e*, which mediates innate immune responses to *Candida* [139]. The related *Clec4f* (KC marker) and *Cd207* (langerin) are located together in a separate locus on Chromosome 6. Each of the *Clec4* genes had a unique expression profile in tissue MPS populations. Analysis of the entire data set reveals that ‘DCIR’ is a misnomer for this family. The DC designation has also been misapplied to other surface receptors, including DC-SIGN (*CD209* in humans), DEC205 (*Ly75*), and DC-HIL (*Gpnmb*). In mice, there are multiple *Cd209* paralogs. *Cd209b* was highly expressed in marginal zone macrophage populations in spleen and is *Csf1r*-dependent [89]. These cells have not been successfully isolated by tissue disaggregation. Four members of the CD209 family (*Cd209a*, *d*, *f*, *g*) were coexpressed in a unique pattern (Cluster 100) together with *Cbr2*, *Ccl24*, and *Clec10a*. *Ly75* was detected in both cDC subpopulations but was most highly expressed in lung macrophages (Cluster 10).

CD64 (*Fcgr1* gene) was used as an exclusion criterion to remove or separate macrophages from DCs or to enrich macrophages in all of the data sets included herein based upon the earlier studies of the ImmGen Consortium [28]. This exclusion was clearly successful in that all the purified DC data sets have very low *Fcgr1* (Fig 2A and S1 Data), but the expression of this gene in macrophage populations was also highly variable. As a simple screen for additional markers that distinguish all CD64^+^ ‘macrophages’ from all CD64^−^ ‘DCs’, we averaged expression across all macrophage and DC samples and compared them (see S1 Data). Amongst the transcripts that were robustly expressed and highly enriched in macrophages to at least the same extent as *Fcgr1*, those encoding surface markers were also variably expressed amongst macrophage populations. However, we identified 3 transcription factor genes—*Cebpb*, *Mafb*, and *Klf10*—that were apparently excluded from all of the CD64^−^ cDCs. The role of *Cebpb* in macrophage differentiation is well-recognised [66, 140, 141], and one of the data sets includes progenitors from *Cebpb*^−/−^ mice [66]. There is evidence of a negative feedback relationship with *Irf8* in monocyte-derived DCs [142]. *Cebpb* was detected in most tissue macrophages but uniquely excluded from some populations, notably the heart and intestinal muscularis. MAFB has been proposed previously as a lineage marker separating macrophages from DCs [143, 144]. However, the most parsimonious explanation of the data would be that MAFB regulates expression of *Fcgr1*. The literature on KLF10 is more limited, with evidence that it participates in TGFβ-induced macrophage differentiation [145].

In overview, although our analysis identifies coregulated clusters associated with cells currently defined as DCs, most proposed markers of this population are clearly shared with other MPS cells, and there is no enrichment for any genes that could confer unique APC activity.

### Resident macrophage activation during isolation

Cluster 41 contains numerous immediate early genes (IEGs) encoding transcription factors and feedback regulators (for example, *Fos*, *Egr1*, and *Dusp1*), consistent with evidence from scRNA-seq of disaggregated cells that isolation of cells from tissues produces cell activation [59, 146]. In many samples, IEGs were amongst the most highly expressed transcripts. The majority of isolated MPS populations also had high levels of macrophage-specific lipopolysaccharide (LPS)-inducible genes. Cluster 224 contains *Ccl2*, *Ccl7*, *Ccl12*, *Cxcl1*, and *Il6*; Cluster 329 includes *Il1b* and *Ptgs2* (also known as *Cox2*); and Cluster 485 contains *Tnf* and inducible chemokines *Ccl3* and *Ccl4*. The anti-inflammatory cytokine *Il10*, which is also LPS-inducible, formed part of the intestinal macrophage cluster (Cluster 38). IL10 is essential to intestinal homeostasis [107], but *Il10* mRNA was detected in only 1 of the 3 intestinal macrophage data sets [37] alongside very high expression of IEGs and proinflammatory cytokine genes (for example, *Il1b*, *Tnf*). The apparent expression of *Fosb* in intestinal macrophages discussed above is likely also an artefact because it is undetectable in total intestinal mRNA (see http://biogps.org). Inflammation-associated transcripts were highlighted as evidence of activation in vivo in sensory neuron-associated macrophages [61]. Similarly, Chakarov and colleagues [27] highlighted selective expression of *Il10* in interstitial lung macrophages and differential expression in the LYVE1^hi^/MHCII^lo^ subpopulation. They did not comment upon the reciprocal pattern observed with *Tnf* and *Il1b*, which were both more highly expressed in the LYVE1^lo^ macrophages. Both populations of interstitial lung macrophages (and all the samples from other tissues in this BioProject) expressed very high levels of all of the IEG transcripts in Cluster 41. Whereas macrophage-expressed transcripts such as *Adgre1* are readily detected in total tissue mRNA and are CSF1R-dependent, inflammatory cytokines and IEG transcripts are not [9, 48]. Accordingly, in each of the RNA-seq data sets we have analysed, the expression of IEGs and inducible cytokines is most likely an artefact of tissue disaggregation and/or cell isolation and separation by FACS.

Interestingly, *Acod1*, which was massively induced within 1 hour by LPS in mouse macrophages in vitro (see http://biogps.org), was only detected at low levels in a small subset of samples and was not correlated with IEGs or any other inflammatory activation markers. Induction of this gene has been attributed functions in adaptive immunometabolism and accumulation of tricarboxylic acid (TCA) cycle intermediates in activated macrophages [147]. The lack of detection in the isolated macrophages suggests either that induction is specific to recruited inflammatory macrophages or that inducible expression is purely an in vitro phenomenon. The *Acod1* expression pattern was correlated only with *Il23a* (encoding a subunit of the cytokine IL23) at the stringency used here (*r* ≥ 0.75).

### Contamination of macrophage populations with other cell types

Table 3 and Fig 5B highlight other clusters that were tissue-specific and contained markers and transcription factors associated with organ/tissue-specific differentiation, with corresponding enrichment for GO terms associated with specific tissues (S2 Data). There are 3 ways in which mRNA from purified macrophage/DC populations may be contaminated with mRNA from unrelated cells. The most straightforward is poor separation of macrophages from unrelated contaminating cells by FACS for purely technical reasons. A second source derives from active phagocytosis by macrophages of dying (senescent/apoptotic) cells, in which RNA from the engulfed cell may be detected. Finally, there is a phenomenon that arises from the extensive ramification of macrophages and their intimate interactions with other cells. Gray and colleagues [148] found that cells purified from LNs with the surface marker CD169 were in fact lymphocytes coated with blebs of macrophage membrane and cytoplasm. Similarly, Lynch and colleagues [149] found that all methods to isolate KCs for flow cytometry produced significant contamination, with CD31^+^ endothelium tightly adhered to remnants of KC membrane.

**Table 3:** Major contaminant clusters.

Cluster Number	Description	Representative Genes
2	General neuronal contamination	*Cacna* family, *Cdh* family, *Chrn* family, *Gabrg1/g2*, glutamate receptors, etc.
5 and 17	Intestinal epithelial	Multiple SLCs, *Cdx1/2*, *Hox* family, *Isx*, *Ihh*
8 and 14	Kidney epithelia	*Pax8*, *Cldn4/8*, *Hnf1b*, *Hoxb2/7*
16	Hepatic parenchymal cells	*Alb*, *C8/9*, *Cyp2* family, *Igfbp1*, *Serpina1*, *Nr1l3*
18	Pancreatic islets	*Ins1*, *Gcg*, *Isl1*
26	Skin/keratinocytes	*Krt4/5/6*, *Stfn*, *Pitx1/2*
32	Bone-marrow–specific, neutrophil contamination	*Elane*, *Camp*, *Fcer1a*, *Gpc1*, *M6s4a3*, *Mpo*, *Prg2/3*, *S100a8/S100a9*, *Gata2*, *Gfi1*, *Cebpe*, *Myb*
33	Immature erythroid	*Hemgn*, *Klf1*
36	Neuronal	*Tnfrsf14*, *Pax6*, *Sox8*
45	Pancreatic acinar cells	*Cel*, *Cpa1*, *Ctrb1*, *Pnlip*
65	Smooth muscle (intestine muscularis	*Acta2*, *Cnn1*, *Des*, *Mylk*, *Tpm1*, *Nkx3-2*
67	NK cells	*Cd3g*, *Cd160*, *Gzma/b/c*, *Il2rb*, *Itga2*, *Kirg1*, *Klra4/7/8/9*, *Klrc2/3*, *Ncr1*
76	Endothelial	*Adgrf5*, *Clec4g*, *Ehd3*, *Flt4*, *Kdr*, *Ptprb*, *Robo4*, *Tie1*, *Sox18*, *Gata4*
87	B cells	*Blk*, *Cd19*, *Cd79a*, *Cxcr5*, *Fcer2a*, *Fcmr*, *Itk*, *Lax1*, *Tnfrsf13c*, *Mef2b*

**Abbreviations:** NK cell, natural killer cell; SLC, solute carrier.

Cluster 2 appears to be a generic ‘rubbish’ cluster, containing transcripts detected at relatively low levels only in specific BioProjects and unrelated to tissue of origin. Other clusters were driven by a single RNA-seq result from within 1 BioProject. These clusters most likely represent technical noise as well as contamination.

Consistent with the proposal from Lynch and colleagues [149], 3 endothelial-associated transcripts—*Cdh5*, *Pecam1*, and *Stab2*—were contained with the KC-enriched cluster (Cluster 12) and apparently increased in expression during KC differentiation. However, other abundant endothelial transcripts were absent. Bonnardel and colleagues [78] generated RNA-seq data from purified liver sinusoidal ECs. We examined the profiles of the most highly expressed EC genes in the macrophage data set. Many of them were detectable in isolated KCs but at much lower levels than *Cdh5*, *Pecam1*, and *Stab2*. They contributed to a separate liver-specific endothelial cluster (Cluster 76). So, whilst there is evidence that ECs contaminate KC preparations, reflecting the close apposition in the sinusoids, *Chd5*, *Pecam1*, and *Stab2* are likely also genuine KC-expressed transcripts.

The detection of mature red cell transcripts encoding haemoglobins (*Hba*, *Hbb*), which are quite abundant in many macrophage populations, most likely reflects ongoing erythrophagocytosis. Macrophages isolated from the intestinal lamina propria in 1 of the 2 large data sets from small intestine [36] were heavily contaminated with markers of intestinal epithelium (Clusters 5 and 17). This might be a separation artefact but could also reflect an active role of macrophages in homeostatic turnover of epithelial cells [150]. Cluster 18 and Cluster 45 were restricted to samples from a study of pancreatic islet and peri-islet macrophage populations [60]. The authors noted the expression of insulin (*Ins1*) mRNA in their islet macrophage populations and attributed it to an intimate interaction with β-cells. Contamination or β-cell–macrophage fusion was said to be excluded on the basis that β-cell markers such as *Pdx1* were not detected. However, many other islet-associated transcripts were abundant and formed part of Cluster 18, notably transcription factors *Isl1*, *Foxa2*, *Nkx6*.*1*, and *Nkx2*.*2*as well as other islet-specific transcripts, *Inhba*, *Chga/b*, *Iapp*, *Gipr*, and *Gcg*. Similarly, Cluster 45 was relatively enriched in the peri-islet macrophages and contains transcripts encoding many pancreatic enzymes. Cluster 65 includes *Acta2* and other smooth muscle markers that selectively contaminated macrophages isolated from the intestinal muscularis [36].

The bone marrow contains several populations of macrophages [33], including those associated with haematopoietic islands expressing CD169 (*Siglec1* gene) and VCAM1. One of the data sets included in the present study profiled the transcriptome of macrophages associated with erythroblastic islands, based upon isolation using an *Epor*-EGFP reporter gene [57]. A second bone marrow data set separated macrophages based upon their engagement in phagocytosis of blood-borne material [65]. The putative erythroblastic island macrophages did not actually express increased *Epor* mRNA (although *Epor* was detected in other macrophage populations as reported recently [96] and fell within Cluster 22). However, in the isolated bone marrow macrophages, *Siglec1* was correlated with high levels of both immature neutrophil (Cluster 32) and erythroid-associated (Cluster 33) mRNAs. The separation of these 2 clusters implies that the contamination occurs in distinct macrophage populations, enriched selectively in each preparation and perhaps derived from separate haematopoietic islands [33]. Cluster 32 also contains the myeloid progenitor transcription factor *Myb* and the GM-progenitor marker *Ms4a3*. Given the extensive ramification of marrow macrophages and their intimate interactions with progenitors [33], this contamination likely reflects the same isolation artefact reported in LN [148], namely haemopoietic progenitor cells cloaked in macrophage clothing.

There are separate clusters including B-cell and natural killer (NK)-cell–specific markers. The B-cell cluster, Cluster 87, shows the highest average expression in intestine, bone marrow, lung, and spleen and likely reflects close association between macrophages and B cells in lamina propria and germinal centres [36]. The cluster containing NK cell markers, Cluster 67, had the highest average expression in one of the DC preparations. Those DCs came from a study that proposed a further subdivision of cDC2s based upon expression of transcription factors T-bet (*Tbx21*) and RORγT [67] and separated cDC2s based upon expression of a *Tbx21* reporter allele. *Tbx21* was detected in all purified splenic cDC preparations presented on http://biogps.org but at much lower levels than in NK cells. NK cells also express *Itgax*, used in purification of the cDCs. Accordingly, it seems likely that apparent *Tbx21* expression in DCs is due to NK cell contamination.

### Clustering of transcription factor expression

**Fig 7:**
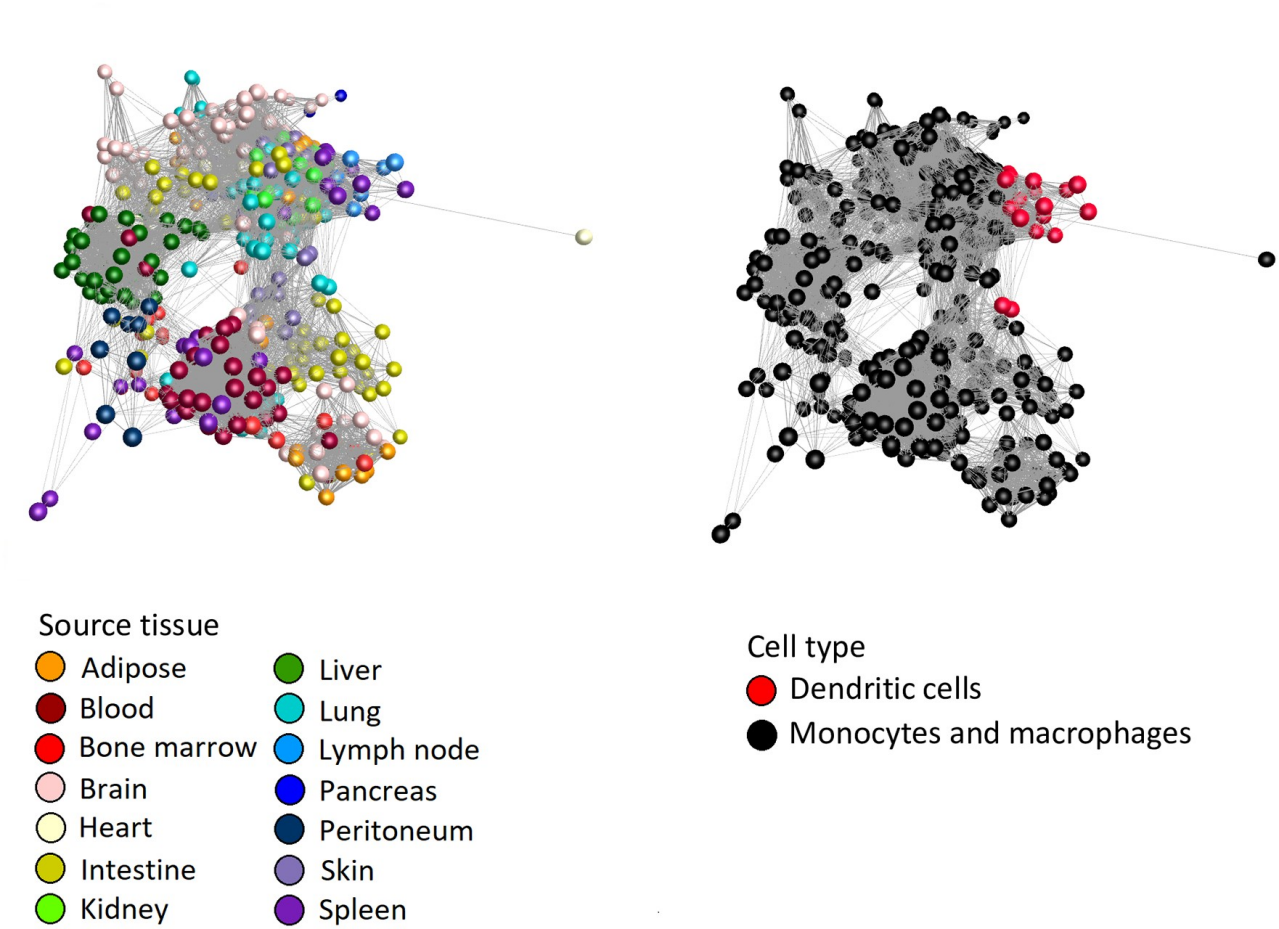
Network analysis of transcription factor gene expression in MPS cell populations. The sample-to-sample network was generated by BioLayout analysis at *r* ≥ 0.66, which included all 466 samples. Nodes representing samples are coloured by source tissue (left) and cell type (right). Lists of genes and expression profiles of clusters at different *r* values are presented in S3 Data. MPS, mononuclear phagocyte system.

Most of the coregulated clusters identified above contain genes encoding transcriptional regulators that are known to be essential for tissue-specific adaptation. These represent only a small subset of the transcription factors detected in MPS cells. The *r* value of 0.75 was selected for the analysis of the whole data set to maximise the number of genes included whilst minimising the number of edges between them (S6 Fig) and aimed at assessing the predictive value of markers including those shown in Fig 2. To test the effect of reducing the stringency, we focussed on annotated transcription factors [151] to reduce the complexity and remove noise. One thousand, one hundred and three transcriptional regulators were detected above the 10 TPM threshold in at least 1 MPS population. The sample-to-sample matrix including all samples formed a single network, including the annotated DCs, as shown in Fig 7. As with the whole data set, increasing the *r* value resulted in separation of various cell populations, but the DCs remained in a group with monocytes/macrophages. We generated GCNs at 3 different Pearson correlation coefficient thresholds, 0.5, 0.6, and 0.7 (S8 Fig). The results are provided in S3 Data. As the cutoff was reduced, more transcription factor transcripts were included in the network. At the highest stringency *r* value (≥0.7), the largest cluster includes *Spi1* alongside many of the transcription factors identified in the largest generic MPS coexpression clusters above (Clusters 1, 3, and 4). We conclude that the basic shared identity of MPS cells involves coordinated expression of around 100–150 transcription factors. Even at the lowest *r* value (≥0.5), transcription factor genes identified as specific to particular tissue-specific MPS populations made few additional connections, indicating that local adaptation is dependent on highly correlated and regulated expression of a small cohort of transcription factors. Nevertheless, associations that become evident at lower *r* values may identify combinatorial interactions in particular cell populations: *Mycl*, associated with DC fitness [152], was weakly correlated with *Irf8* and *Zbtb46*; *Cebpb* with *Nfil3*; and interferon-related transcription factors (*Batf2*, *Irf1/7/9*, *Stat1/2*) were connected at the threshold of 0.5 (S3 Data).

### Expression of solute carriers and metabolism genes in MPS cell populations

The burgeoning field of immunometabolism has focussed on regulation of intermediary metabolism in recruited monocytes and macrophages in various states of activation or polarisation [147]. Amongst emerging concepts is the view that M1 polarisation (classical activation) is associated with aerobic glycolysis and mitochondrial dysfunction, whereas M2 polarisation requires an active tricarboxylic acid cycle [147]. We used the MPS transcriptome to infer likely metabolic adaptations of tissue-resident MPS cells.

Cluster 7 contains mitochondria-associated transcripts and transcripts encoding ribosomal subunits, with variable expression across all samples even from the same tissue, indicating that resident tissue macrophages vary in their dependence upon mitochondrial oxidative phosphorylation irrespective of surface markers or differentiation state.

In many cases, metabolic pathways are regulated at the level of solute transport [147]. There were > 400 members of the SLC family expressed in mononuclear phagocytes above the 10 TPM threshold. Some were more highly expressed in intestine and kidney epithelial cells and clustered with tissue-specific epithelial markers. However, many contributed to macrophage-enriched expression clusters. One such gene, *Slco2b1*, which encodes an organic anion transporter of unknown function, has been proposed as a marker gene to distinguish macrophages from DC subsets, and the promoter was used in an inducible macrophage depletion strategy [27]. The larger data set herein does not support this dichotomy. *Slco2b1* is part of Cluster 4, enriched in microglia and absent from multiple other macrophage populations, in addition to both cDC subsets.

Macrophages depend to varying degrees upon glutamine, glucose, and fatty acids as fuels [153], and glutamine is an important immune regulator [154]. 14 different solute carriers from 4 families have been shown to transport glutamine [155]. Of the genes encoding these carriers, *Slc38a1* was widely expressed in MPS cells and did not fall within a cluster, whereas *Slc7a5*, *Slc7a7*, *Slc7a8*, and *Slc38a7* were part of distinct macrophage-enriched clusters. Consistent with the importance of glutamine as a fuel for resident MPS cells, transcripts encoding enzymes of glutamine metabolism (*Gls*, *Glud1*, *Glul*, *Slc25a11*) were also highly expressed and part of Clusters 1 and 3. By contrast, resident MPS cells apparently have very limited expression of glucose transporters. *Slc2a1* (encoding glucose transporter GLUT1) was low, highly variable, and idiosyncratic amongst tissues. A myeloid-specific conditional knockout of *Slc2a1* confirmed that GLUT1 was the major glucose transporter in macrophages analysed in vitro, but the loss of glucose as a fuel had remarkably little impact on macrophage function [156]. The expression of *Slc2a1* in cells isolated from tissues is difficult to interpret because the transporter is induced by hypoxia [157], which might arise during isolation.

In the absence of *Slc2a1*, macrophages in vitro increased oxidation of fatty acids [156]. The *Slc27a1* gene, encoding the fatty acid transporter FATP1—which also contributes to functional regulation in macrophages [158, 159]—was widely expressed in tissue macrophages and, with carnitine acyl transferase genes (*Crat*, *Crot*), formed part of Cluster 1. *Slc2a5* (found in Cluster 4) encodes a fructose-specific transporter [160] and was expressed primarily in microglia. *Slc2a6* is a lysosome-associated glucose transporter that was recently knocked out in the mouse genome [161]. It also has a novel expression profile, being highest in monocytes and cDC2s.

One of the best known functional solute carriers in macrophages is natural resistance associated membrane protein 1 (NRAMP1; *Slc11a1* gene), which is associated with genetic resistance to intracellular pathogens. SLC11A1/NRAMP1 is expressed in lysosomes and contributes to pathogen resistance by restricting available iron [162]. The role in iron metabolism is reflected by its presence in Cluster 12 alongside *Slc40a1*, encoding ferriportin, the macrophage-enriched iron exporter [163]. One other prominent class of solute carriers highly expressed in macrophages (*Slc30a6*, *Slc30a7*, *Slc30a9*, *Slc39a3*, *Slc39a7*, and *Slc39a9* in Cluster 1 and *Slc39a12* in Cluster 4) is involved in transport of zinc, which is a component of antimicrobial defence [164, 165]. Two further zinc transporters, *Slc39a2* and *Slc39a11*, were enriched in lung macrophages (Cluster 10). This lung-macrophage–enriched cluster also contains *Slc52a3*, encoding a riboflavin transporter, *Slc6a4* (sodium- and chloride-dependent sodium symporter), and 2 members of the Slc9 family of sodium–hydrogen exchange (NHE) transporters (*Slc9a4* and *Slc9a7*), which are more traditionally associated with epithelial function [166].

### Validation of coexpression clustering with an independent kidney data set

The abundant macrophage populations of the kidney were first described in detail using F4/80 as a marker in situ [167]. There has been considerable debate about the relationships between resident macrophages, monocyte-derived macrophages, and cDC subsets in the kidney [168]. The main cluster analysis did not reveal a separate kidney-resident macrophage-enriched profile. The kidney data set in the preceding analysis included F4/80^+^, CD64^+^ macrophages isolated from control and ischaemic kidneys, further subdivided based upon expression of CD11B and CD11C [54]. Salei and colleagues [70] recently produced RNA-seq data for isolated populations of resident macrophages, monocyte-derived cells, cDC1s, and cDC2s from kidney compared with similar populations from spleen. The primary data were not available for download by our automated pipeline through the ENA at the time we pooled and froze our data set (February 2020). We therefore obtained the processed data directly from the authors and carried out network analysis using the 33 samples and 9,795 genes with normalised expression of at least 10 in at least 1 sample. This analysis served to validate the approach to analysis and the core conclusions using an independent data set.

**Fig 8:**
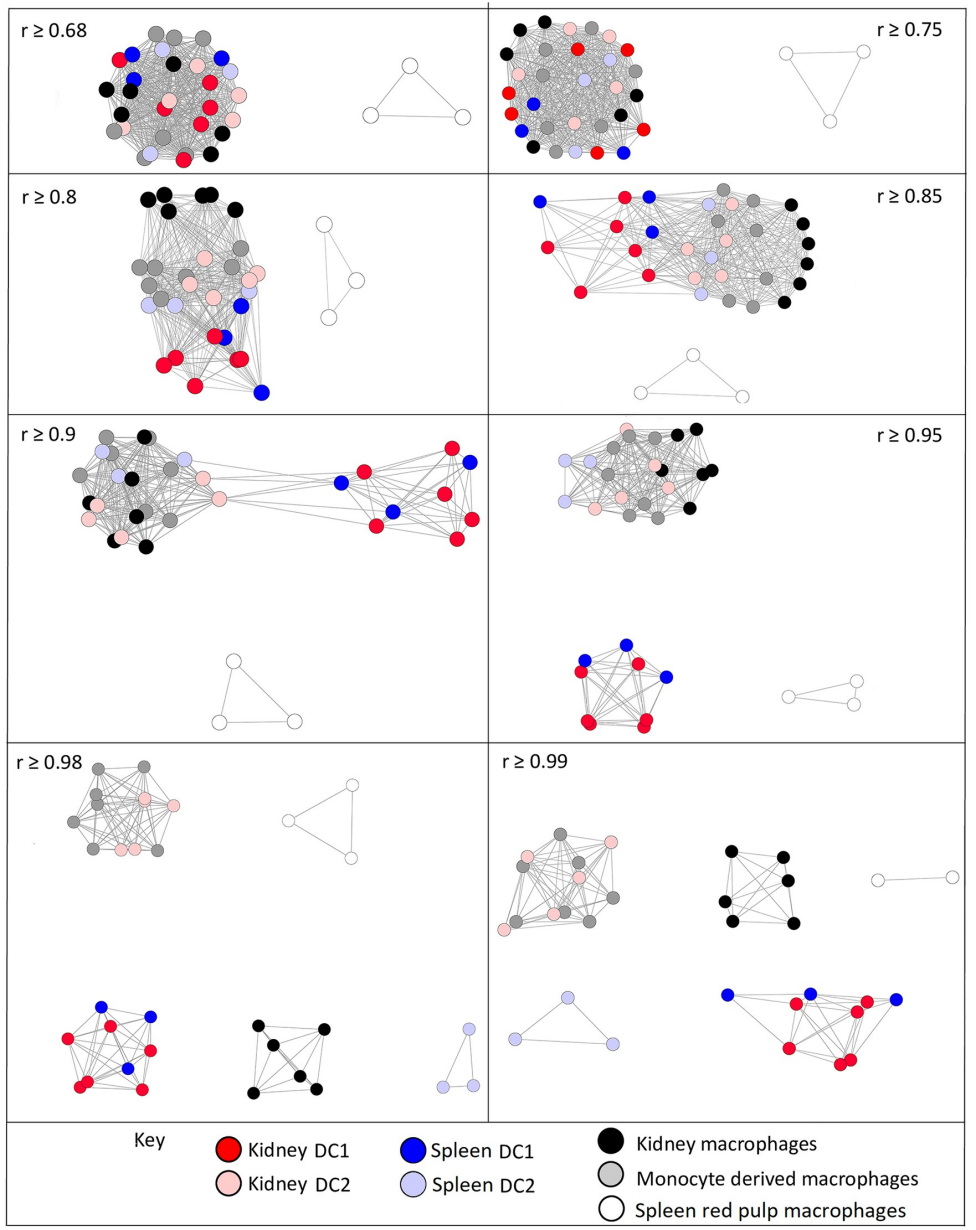
Sample-to-sample 2D network analysis of gene expression in macrophage and DC subpopulations from kidney and spleen. The sample-to-sample network was generated by BioLayout analysis at the indicated Pearson *r* values, which all included all 33 samples up to *r* ≥ 0.98. Above *r* ≥ 0.98, 1 red pulp macrophage sample was lost. Red, kidney DC1; pink, kidney DC2; dark blue, spleen DC1; light blue, spleen DC2; black, kidney macrophages; grey, monocyte-derived macrophages; white, spleen red pulp macrophages. Lists of genes and expression profiles of clusters are presented in S4 Data. DC, dendritic cell.

The macrophages of the kidney are intimately associated with the capillaries [167, 169], but *Lyve1* was not detectable in resident macrophages in this data set or in [54]. Published IHC on mouse kidney reveals that LYVE1 is restricted to lymphatic vessels [170]. Fig 8 illustrates the way in which the sample-to-sample matrix revealed relationships between the cell populations with increasing correlation coefficient threshold. Even at the lowest correlation cutoff used in the main atlas (0.75), the splenic red pulp macrophages separated from all kidney and DC samples. As the cutoff was made more stringent, the cDC1s from both spleen and kidney separated, but the resident kidney macrophages, cDC2s, and monocyte-derived macrophages remained closely connected until *r* ≥ 0.98, when the spleen cDC2s separated from the monocyte-derived macrophages and kidney cDC2s. At *r* ≥ 0.99, the kidney cDC2s and monocyte-derived macrophages were still not separated, indicating that the expression profiles of these cell types are very similar. Salei and colleagues [70] performed a principal components analysis based upon the 500 most variable transcripts and also identified the close relationship between cDC2s and monocyte-derived cells. Our analysis further emphasises their conclusion that the main axis of difference is between spleen macrophages and all other cells. cDC1s from both tissues were more similar to each other than to the other cells, but spleen cDC2s were only separated from kidney cDC2s and monocyte-derived macrophages at the highest stringencies. We also performed a gene-to-gene analysis on these data. The profiles of kidney myeloid cells other than cDC1s were very similar and differed by only a small number of genes. Consistent with this conclusion, the 2 largest clusters in this analysis (see S4 Data) were shared between all of the isolated populations and contain *Spi1* as well as many of the DC-enriched markers identified in the main analysis. However, *Ccr7* and many of the genes associated with it in the main data set (Cluster 13, Table 2; for example, *Spib*, *Stat4*, *Vsig10*, *Cd200*, and *Itgb8*) were expressed at low levels in isolated kidney DCs as in lung DCs. Cluster 3 of the kidney analysis was specific to splenic red pulp macrophages and contains the known transcriptional regulators *Pparg*, *Spic*, and *Nr1h3*. Transcripts in Cluster 4 were enriched in the resident kidney macrophages compared to both splenic macrophages and other kidney myeloid populations. Interestingly, the resident kidney macrophage cluster includes many genes that are also highly expressed in microglia and depleted in the brain in *Csf1r* mutant mice and rats, including *Cx3cr1*, *C1qa/b/c*, *Csf3r*, *Ctss*, *Fcrls*, *Hexb*, *Laptm5*, *Tgfbr1*, *Tmem119*, and *Trem2* [17, 89]. These were also detected in the isolated kidney macrophages in S1 Data. Both microglia and resident F4/80^hi^ kidney macrophages are selectively lost in a mouse line with a mutation in a conserved enhancer of the *Csf1r* locus [17]. RUNX1 regulates the activity of the *Csf1r* enhancer [171] and has also been implicated in the establishment of microglial cells during development [172]. The *Runx1* gene was within this cluster. *Csf1r* mRNA was expressed at high levels in cells defined as cDC2s, as well as monocyte-derived cells and resident macrophages. All cells expressing a *Csf1r*-EGFP reporter in the kidney were depleted by treatment with anti-CSF1R antibody [34]. This suggests that despite their expression of FLT3, renal cDC2s are CSF1R-dependent, a conclusion consistent with previous evidence that cells classified as cDC2s in other nonlymphoid tissues are dependent upon CSF1R rather than FLT3 [173]. Cluster 6 of the kidney analysis, including *Itgam*, was enriched as expected in the selected CD11B^+^ populations from kidney but highly expressed in all of the populations. This cluster includes all of the co-regulated IEGs identified in Cluster 41 in the extended MPS data set above, suggesting that recent monocyte-derived cells may be more susceptible to activation during isolation. In summary, sample-to-sample and gene-to-gene networks on this smaller independent data set are entirely consistent with conclusions from the global MPS analysis that question the basis for the separation of DC from macrophages using surface markers.

### The relationship between single-cell and bulk RNA-seq data

The advent of scRNA-seq has been heralded as a revolution, promising new approaches to classification of myeloid heterogeneity [26, 174, 175]. To determine whether scRNA-seq produces distinct insights about MPS heterogeneity, we wished to assess the relationship between the coexpression networks we have generated from large total RNA-seq data sets and the coexpression modules inferred from scRNA-seq. scRNA-seq is an intrinsically noisy, nonquantitative stochastic sampling of a subset of the most abundant mRNAs in individual cells [176, 177]. Algorithms that support nonlinear dimensional reduction (for example, *t*-distributed stochastic neighbour embedding [*t*-SNE] or Uniform Manifold Approximation and Projection [UMAP]) [178] followed by some form of clustering are then used to join together groups of cells in which the members share detectable expression of an arbitrarily defined set of markers. The number of populations defined depends upon the parameters applied, and different approaches do not always give the same answers [178]. There is an implicit assumption in this approach that defined cell types with approximately identical transcriptomes actually exist and that sampling noise can be overcome by analysis of a sufficiently large number of cells. Based upon scRNA-seq analysis of interstitial lung macrophages, Chakarov and colleagues [27] inferred the existence of a subpopulation that selectively expressed *Lyve1*. They then generated bulk RNA-seq data from separated LYVE1^hi^ and LYVE1^lo^ subpopulations. Their data uniquely support a critical comparison of the 2 approaches to transcriptome analysis and the outcomes of our network analysis. For this purpose, the primary scRNA-seq data were downloaded, reanalysed, and expressed as TPM using the Kallisto pseudoaligner, as described in the ‘Materials and methods’. S5 Data contains these reprocessed scRNA-seq data, alongside the bulk RNA-seq data for the lung macrophage subpopulations from the same study, with the level of expression ranked based upon the bulk RNA-seq data for the purified LYVE1^hi^ interstitial macrophages.

**Fig 9:**
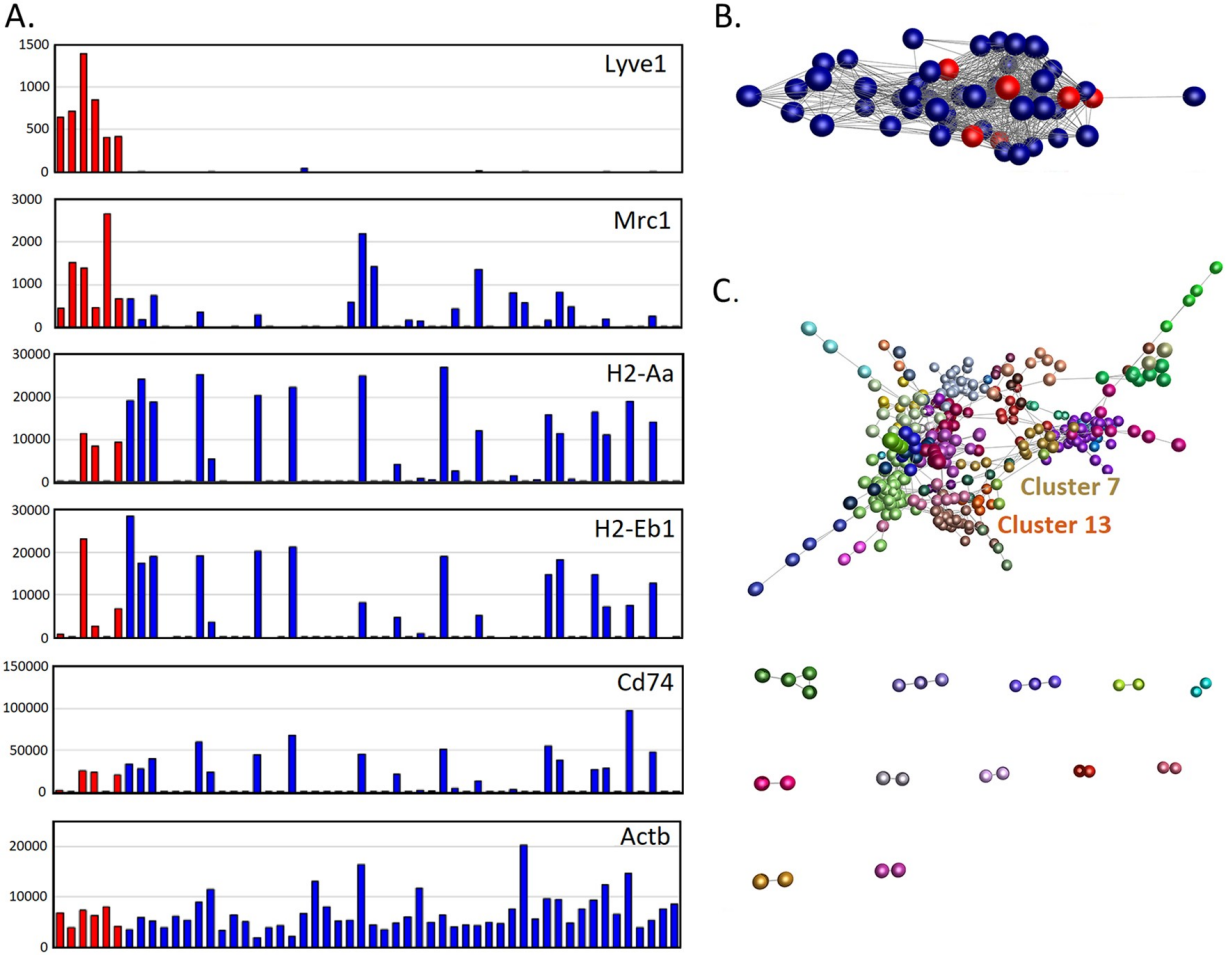
Network analysis of scRNA-seq data. The reprocessed data and the bulk RNA-seq data for the lung macrophage subpopulations from the same study are available in S5 Data. (A) Expression profiles in single cells for selected genes. Each column represents RNA from a single cell. *Y* axis shows expression in TPM, calculated using Kallisto. Only the first 6 cells expressed *Lyve1* (coloured red). (B) The sample-to-sample network was generated by BioLayout analysis at *r* ≥ 0.53, which included all 54 single-cell samples. Nodes represent samples; red nodes show the samples with high expression of *Lyve1*. (C) Gene-to-gene network (*r* ≥ 0.5), clustered at MCL inflation value of 1.7. Cluster lists and expression profiles are available in S6 Data. MCL, Markov clustering algorithm; RNA-seq, RNA sequencing; scRNA-seq, single-cell RNA-seq; TPM, transcripts per million.

Consistent with Zipf’s law, the power-law distribution of transcript abundance [71, 72], the top 200 expressed transcripts in the bulk RNA-seq data (approximately 1% of the total) contribute around 50% of the total detected transcripts in the scRNA-seq data (S5 Data). The abundant transcripts from bulk RNA-seq that were also detected in scRNA-seq samples include many cell-type–specific surface markers, which explains the ability to use scRNA-seq to discover such markers. These abundant transcripts also include IEGs such as *Dusp1*, *Egr1*, *Fos*, *Ier2*, and *Junb*, indicative of the activation that occurs during isolation as discussed above. The inducible cytokines, including *Ccl2*, *Tnf*, *Il1b*, *Il6*, and *Il10*, were each detected in a subset of the single cells, most likely also induced during isolation. Of the 200 most highly expressed transcripts identified in the bulk RNA-seq data, only a very small subset (including *Actb*, *Apoe*, *B2m*, *Ccl6*, *Cd74*, *Ctsb*, *Fth1*, *Ftl1*, and *Lyz2*) had nonzero values in all cells in the scRNA-seq output. The average expression of the top 500 transcripts in the single cells was similar to the bulk RNA-seq, but the detected expression level varied over 4 orders of magnitude amongst individual cells. *Fcgr1* and *Mertk* mRNAs, encoding markers used to purify the interstitial macrophages for scRNA-seq, as well as other commonly used markers (*Cx3cr1*, *Itgax*) were actually detected in only a small subset of the cells and were not correlated with each other. Both this study and a subsequent study [76] state that *Mrc1* and *Lyve1* expression is shared by overlapping populations of lung interstitial macrophages. That conclusion is not supported by the data. Even in the bulk RNA-seq data from lung interstitial macrophages, the expression of *Mrc1* was only marginally enriched in purified LYVE1^hi^ cells relative to LYVE1^lo^ cells (S1 Data). The separation of these 2 markers was evident from the separate study of lung interstitial macrophage populations [52] included in our global MPS analysis and has been discussed above. Consistent with that conclusion, in the scRNA-seq data, the two are not strictly correlated with each other, with *Mrc1* being detected in many more cells than *Lyve1* (Fig 9A) despite similar absolute levels of expression in the total RNA-seq data.

To identify whether any robust correlations actually exist in the scRNA-seq data, the top 500 expressed transcripts in the scRNA-seq samples were used for network analysis. The sample-to-sample network (*r* ≥ 0.53) is shown in Fig 9B, and the gene-to-gene network (*r* ≥ 0.5) in Fig 9C. The cluster list and average expression profiles are provided in S6 Data. One clear-cut finding is the coexpression of genes involved in APC activity (*H2-Aa*, *H2-Ab1*, *H2-Eb1*, *Cd74*, and *Ctss*; Cluster 13 of the scRNA-seq analysis, indicated in Fig 9C), which were effectively present or absent in individual cells. Chakarov and colleagues [27] defined 2 subpopulations as LYVE1^hi^/MHCII^lo^ and LYVE1^lo^/MHCII^hi^, but only 6 of the scRNA-seq samples expressed *Lyve1*, and only half of those also expressed detectable MHC II genes (Fig 9A). This is consistent with the lack of any inverse correlation between *Lyve1* and *Cd74* in Fig 2B. Even at this low *r* value, known highly expressed markers segregated from each other. *Lyve1* forms a cluster with *Mgl2*, *Cd209*, and *Cd302* (Cluster 7 in the scRNA-seq analysis; Fig 9C). *Adgre1* is in a coexpression cluster that includes *Lyz2* and *Msr1* (Cluster 4 of the scRNA-seq analysis), *Csf1r* is coexpressed with *Mrc1* and *Cd163* (Cluster 2), and *Lgals3* with *Retnla* and *Fcrls* (Cluster 1). The coregulation of MHC-related genes and genes located in the same chromosomal region (for example, *C1qa*, *C1qb*, *C1qc*; Cluster 25 of the scRNA-seq analysis), as well as the relatively uniform detection of genes such as *Actb* (Fig 9A), suggests that a significant proportion of the all-or-nothing differences in expression between cells in the scRNA-seq data is real. That conclusion may be considered a reflection of the limitations of the technology, but it is actually supported by other evidence. Tan and Krasnow [179] defined subpopulations of interstitial lung macrophages based upon expression of F4/80, Mac-2 (*Lgals3*), and Class II MHC and tracked the changes in their relative abundance during development. Interestingly, they did not detect LYVE1 on adult lung interstitial macrophages by IHC. Consistent with their data, in the scRNA-seq data examined here, most lung interstitial macrophages expressed high levels of either *Adgre1* or *Lgals3*, but some expressed both or neither.

Across our data set, the selection of MPS populations based upon surface markers was also strongly enriched for the mRNA encoding those markers (for example, CD11C^+^ cells express *Itgax*). The reciprocal relationship need not be the case. Protein expression at a single-cell level clearly does not vary to the same extent as mRNA because proteins have different rates of translation, turnover, and decay [180]. Markers such as F4/80 and CD11C and transgenes based upon macrophage-enriched promoters such as those of *Csf1r* and *Cx3cr1* do appear to label the large majority of MPS cells in most tissues. The disconnect between scRNA-seq and cell surface markers may partly reflect the nature of transcription. At the single-cell level, transcription occurs in pulses interspersed by periods of inactivity and mRNA decay, which can manifest as random monoallelic transcript expression [181]. If gene expression is genuinely probabilistic at the level of individual loci [180], the assumption of transcriptomic homogeneity in definable cell types upon which scRNA-seq analysis is based is clearly invalid. The number of macrophage subpopulations that can be defined in any scRNA-seq data set becomes a matter of choice and model. As an extreme example, one recent scRNA-seq study identified 25 distinct myeloid cell differentiation ‘states’ in a mouse lung cancer model [182].

### A critical view of the validity of markers

The RNA-seq data included as representative of cDC subsets [67] were from cells purified using CD64 as a marker to exclude macrophages. Despite this choice, an unbiased assessment of the sample-to-sample networks in Figs 3B and 4 (based on all genes) and Fig 7 (based on transcription factor genes) would class all of these DCs as part of the same family as monocytes and macrophages. The use of CD64 as a definitive marker distinguishing macrophages from DCs was criticised when it was proposed [44], and it remains untenable. It is actually a curious choice as a marker to define a cell as a macrophage because the encoded protein FCGR1 (CD64) has been implicated functionally in APC activity [183]. Interestingly, a recent study of inflammation in the lung now posits the existence of CD64^++^ cDC2s and suggests that CD26 (encoded by *Dpp4*) is a more definitive marker [184]. In the separate kidney data set [119], monocyte-derived macrophages were separated from cDC2s based upon CD64 as a marker. Indeed, the cDC2s lacked *Fcgr1* expression, but there was no enrichment for *Dpp4*. As noted above, despite the use of CD64 as a marker, the transcriptomes of the 2 populations (cDC2s and monocyte-derived macrophages) were almost indistinguishable. Like other MPS cells, DC expression profiles may also be influenced by tissue-specific signals. Both the larger data set and the kidney data [70] suggest that there is tissue-specific adaptation of ‘cDC2s’ that may remain more ‘macrophage-like’, which may be a reflection of their dependence upon CSF1R. From our analysis, the proposed separation of DCs from all other members of the MPS based upon APC function, surface markers, transcription factors, or ontogeny [21] remains problematic [19].

The concept of M1/M2 polarisation derives from analysis of classical and alternative activation of recruited monocytes by Th1 (interferon gamma [IFNγ]) and Th2 (IL4/IL13) cytokines [31]. Previous meta-analysis indicated that proposed M2 markers defined by others [31] correlate poorly with each other in isolated inflammatory macrophages and are not conserved across species [30]. The M1/M2 concept was also challenged in a recent comparative analysis of in vitro and in vivo data on macrophage gene expression [185], which concluded that ‘valid *in vivo* M1/M2 surface markers remain to be discovered’. We would suggest that they do not exist; each proposed marker also has its own unique transcriptional regulation and tissue-specific function in resident macrophages. Aside from proposed M2 markers already mentioned that each have idiosyncratic expression (*Mrc1*, *Retnla*, *Igf1*, *Mgl2*), *Chil3* (also known as Ym1) was highly expressed in lung macrophages (Cluster 10 of the whole data set analysis; S2 Data), *Arg1* and *Alox15* were restricted to peritoneal macrophages (Cluster 21), and *Cd163* was part of a small cluster of 4 transcripts (Cluster 312). Detection of M2 markers on resident macrophages cannot imply that they share any functions with alternatively activated recruited monocytes. Nevertheless, IL4/IL13/STAT6 signalling could contribute to resident MPS cell differentiation. The IL13 receptor (*Il13ra*) is part of the generic MPS Cluster 1, and *Il4ra* is also highly and widely expressed. IL4 administration to mice can drive resident tissue macrophage proliferation beyond levels controlled homeostatically by CSF1 [186].

### How do transcriptional networks contribute to understanding macrophage heterogeneity in situ?

One concern with analysis of the cells isolated by tissue digestion and evaluated here is whether recovered cells are representative of the tissue populations. Several macrophage populations have resisted isolation, notably those of the marginal zone of spleen and subcapsular sinus of LNs and the abundant macrophage populations detected in skeletal muscle [5], whilst others are clearly fragmented during isolation as discussed above. Given the remarkable ramification of resident MPS cells in situ, it seems unlikely that they are quantitatively recovered intact in any isolation protocol. Subpopulations of isolated cells defined by markers can sometimes be linked to precise location within the tissue. One recent example is the clear separation of distinct myeloid populations in the liver. A unique subcapsular MPS cell population distinct from KC was initially classified as DC-like based upon expression of CD11C and apparent lack of macrophage markers [187], but these cells were subsequently characterised as F4/80^+^, CSF1R-dependent macrophages uniquely expressing CD207 [188]. Another is the apparent location of LYVE1^hi^ macrophages with capillaries in the lung [27]. On the other hand, it is unclear where the putative long-lived CD4^+^, TIM4^+^ population in the gut [37] is located. In broad overview, macrophages in every organ, detected with *Csf1r* reporter transgenes that are expressed in all myeloid cells including DCs, have a remarkably regular and uniform spatial distribution. The concept of a macrophage territory [5] or a niche [189, 190] has been proposed. Despite this apparent homogeneity in location and morphology, multicolour immunolocalisation of macrophage surface markers indicates that they are almost infinitely heterogeneous (reviewed in [24]). Some of this variation may be purely stochastic [180]. However, most of the data sets analysed here suggest that monocytes and macrophages in each organ are a differentiation series. We have taken the view that macrophages in tissues have a defined half-life such that some cells survive by chance and continue to change their gene expression [5]. Each macrophage that occupies a new territory, following either infiltration as a monocyte or self-renewal by cell division, starts a life history that involves changes in gene expression and surface markers with time. In that view, many MPS subpopulations may be no more than arbitrary windows within a temporal profile of adaptation.

### Conclusion

The transcriptional network analysis confirms that using our unique approach to downsizing and a common quantification pathway, the RNA-seq data from different laboratories can be merged to provide novel insights. The network analysis indicates the power of large data sets to detect sets of coregulated transcripts that define metastable states of MPS adaptation and function. The merged data set we have created provides a resource for the study of MPS biology that extends and complements resources such as ImmGen (http://www.immgen.org). It can be readily expanded to include any new RNA-seq data for comparative analysis, and clusters can be regenerated using BioLayout or the further development of this network approach, Graphia, which are freely available (http://biolayout.org; https://graphia.app). For example, 2 recent studies have profiled isolated macrophage subpopulations from peripheral neurons [191, 192].

Clusters of transcripts that are robustly correlated give clear indications of shared functions and transcriptional regulation. However, our analysis also revealed 2 important artefacts in the study of isolated tissue macrophages: the clear evidence of inflammatory activation during isolation and the extensive contamination of isolated preparations with transcripts derived from other cell types. One recent innovation that may obviate these issues is the use of a so-called MacTrap transgene to isolated macrophage-specific polysome-associate mRNA from tissues without isolating the cells [193].

A discussion review of MPS heterogeneity in 2010 [194] suggested that in order for the field of immunology to advance and communicate, ‘all cells have to be called something’. This Linnaean view continues to drive efforts to classify MPS cells into subsets based upon markers. The analysis we have presented shows that surface markers are poorly associated with each other and have very limited predictive value. Aside from Class II MHC, there are no markers that can be correlated with predicted APC activity. Resident tissue MPS cells, including cells that are currently defined as DCs, belong to a closely related family of cells in which the transcriptomic similarities are much greater than the differences. The cumulative function of the population of MPS cells acting together within each tissue is likely to be more important to homeostasis and immunity than the individual heterogeneity.

## Supporting information

S1 Fig Spearman correlation coefficients. (A) Spearman correlation coefficients for expression patterns of different housekeeping genes compared with the Pearson correlation coefficients from Fig 1. (B) Spearman correlation coefficients for expression patterns of different MPS genes compared with the Pearson correlation coefficients from Fig 2. MPS, mononuclear phagocyte system.
(PDF)

S2 Fig Sample-to-sample 2D network analysis of gene expression in monocyte, macrophage, and DC populations. Each sphere (node) represents a sample, and lines between them (edges) show Pearson correlations between them of ≥0.85. The network includes 458 samples. (A) Samples coloured by tissue of origin. (B) Samples coloured by cell type. (C) Samples coloured by BioProject. DC, dendritic cell.
(PDF)

S3 Fig Sample-to-sample 2D network analysis of gene expression in monocyte, macrophage, and DC populations. Each sphere (node) represents a sample, and lines between them (edges) show Pearson correlations between them of ≥0.95. The network includes 418 samples. (A) Samples coloured by tissue of origin. (B) Samples coloured by cell type. (C) Samples coloured by BioProject. DC, dendritic cell.
(PDF)

S4 Fig Sample-to-sample 2D network analysis of gene expression in monocyte, macrophage, and DC populations. Each sphere (node) represents a sample, and lines between them (edges) show Spearman correlations between them of ≥0.85. The network includes 443 samples. (A) Samples coloured by tissue of origin. (B) Samples coloured by cell type. (C) Samples coloured by BioProject. DC, dendritic cell.
(PDF)

S5 Fig Sample-to-sample 2D network analysis of gene expression in monocyte, macrophage, and DC populations. Each sphere (node) represents a sample, and lines between them (edges) show Spearman correlations between them of ≥0.9. The network includes 427 samples. (A) Samples coloured by tissue of origin. (B) Samples coloured by cell type. (C) Samples coloured by BioProject. DC, dendritic cell.
(PDF)

S6 Fig Graph size compared with correlation threshold for the analysis of the mouse macrophage data set. The chosen correlation threshold of 0.75 resulted in inclusion of 12,775 nodes, making 1,113,125 edges (correlations of ≥0.75) between them.
(PDF)

S7 Fig Average expression of genes in Cluster 12 during differentiation of monocytes to KCs. Data from BioProject PRJNA528435. *Clec4f*-cre Rosa26iDTX mice were treated with DTX to remove mature KCs. Livers were harvested at indicated time points after DTX treatment. Control animals were treated with PBS and harvested at 72 hours. The experiment shows the repopulation of the liver with cells derived from monocytes. DTX, diphtheria toxin; KC, Kupffer cell.
(PDF)

S8 Fig Graph size compared with correlation threshold for the analysis of the mouse macrophage transcription factor data set. Red line shows the highest threshold to include all 1,103 nodes (*r* ≥ 0.28). Black broken lines show the 3 correlation thresholds used in the analysis: *r* ≥ 0.5 (1,064 nodes), *r* ≥ 0.6 (949 nodes), and *r* ≥ 0.7 (714 nodes).
(PDF)

S1 Data Excel spreadsheet containing gene expression data for all MPS samples expressed as TPM. Separate sheet highlights genes of interest encoding surface markers and transcription factors. Analysis includes means, standard deviation, CoV, and Mac:DC expression ratios. CoV, coefficient of variance; DC, dendritic cell; Mac, macrophage; MPS, mononuclear phagocyte system.
(XLSX)

S2 Data Cluster lists for the gene-centred network analysis of the complete MPS data set including graphs of average expression profiles. Separate sheet shows the GO term enrichment scores for each cluster. GO, gene ontology; MPS, mononuclear phagocyte system.
(XLSX)

S3 Data Clusters lists for gene-centred network analysis of transcripts encoding transcription factors at 3 *r* values: 0.5, 0.6, and 0.7. (XLSX)

S4 Data Cluster lists for the expression data for kidney and spleen MPS populations from [119]. MPS, mononuclear phagocyte system.
(XLSX)

S5 Data Excel spreadsheet containing expression data for scRNA-seq and total RNA-seq from lung MPS populations from [27]. MPS, mononuclear phagocyte system; RNA-seq, RNA sequencing; scRNA-seq, single-cell RNA-seq.
(XLSX)

S6 Data Cluster lists for the GCN analysis of lung MPS scRNA-seq data from [27] including graphs of average expression profiles. GCN, gene coexpression network; MPS, mononuclear phagocyte system; RNA-seq, RNA sequencing; scRNA-seq, single-cell RNA-seq.
(XLSX)

## Abbreviations

APC: antigen-presenting cell
cDC: classical DC
CDP: common DC progenitor
CoV: coefficient of variance
CSF1R: macrophage-colony–stimulating factor receptor
DC: dendritic cell
DCIR: DC immunoreceptor
EC: endothelial cell
EGF: epidermal growth factor
EGFP: enhanced green fluorescent protein
ENA: European Nucleotide Archive
ER: endoplasmic reticulum
FACS: fluorescence activated cell sorting
FATP1: fatty acid transporter 1
GCN: gene coexpression network
GLUT1: glucose transporter 1
GM: granulocyte macrophage
GO: gene ontology
IEG: immediate early gene
IFNγ: interferon γ
IHC: immunohistochemistry
KC: Kupffer cell
LC: Langerhans cell
LN: lymph node
LPS: lipopolysaccharide
MHC: major histocompatibility complex
Mincle: macrophage-inducible C-type lectin
MPS: mononuclear phagocyte system
NAIP: neuronal apoptosis inhibitory protein
NCBI-GEO: National Center for Biotechnology Information Gene Expression Omnibus
NHE: sodium-hydrogen exchange
NK cell: natural killer cell
NRAMP1: natural resistance associated membrane protein 1
qRT-PCR: quantitative reverse transcriptase polymerase chain reaction
RNA-seq: RNA sequencing
scRNA-seq: single-cell RNA-seq
SLC: solute carrier
SRA: Sequence Read Archive
TCA: tricarboxylic acid
TGFβ: transforming growth factor β
TPM: transcripts per million
*t*-SNE: *t*-distributed stochastic neighbour
UMAP: Uniform Manifold Approximation and Projection

## References

[1] van Furth R, Cohn ZA, Hirsch JG, Humphrey JH, Spector WG, Langevoort HL. The mononuclear phagocyte system: a new classification of macrophages, monocytes, and their precursor cells. Bull World Health Organ. 1972;46(6):845–52. 4538544 PMC2480884

[2] Amit I, Winter DR, Jung S. The role of the local environment and epigenetics in shaping macrophage identity and their effect on tissue homeostasis. Nat Immunol. 2016;17(1):18–25. 10.1038/ni.3325 26681458

[3] Hoeffel G, Ginhoux F. Fetal monocytes and the origins of tissue-resident macrophages. Cell Immunol. 2018;330:5–15. 10.1016/j.cellimm.2018.01.001 29475558

[4] Hoeksema MA, Glass CK. Nature and nurture of tissue-specific macrophage phenotypes. Atherosclerosis. 2019;281:159–67. 10.1016/j.atherosclerosis.2018.10.005 30343819 PMC6399046

[5] Hume DA, Irvine KM, Pridans C. The Mononuclear Phagocyte System: The Relationship between Monocytes and Macrophages. Trends Immunol. 2018;40, 98–112 10.1016/j.it.2018.11.007 30579704

[6] Guilliams M, Thierry GR, Bonnardel J, Bajenoff M. Establishment and Maintenance of the Macrophage Niche. Immunity. 2020;52(3):434–51. 10.1016/j.immuni.2020.02.015 32187515

[7] Chitu V, Stanley ER. Regulation of Embryonic and Postnatal Development by the CSF-1 Receptor. Curr Top Dev Biol. 2017;123:229–75. 10.1016/bs.ctdb.2016.10.004 28236968 PMC5479137

[8] Hume DA, Caruso M, Ferrari-Cestari M, Summers KM, Pridans C, Irvine KM. Phenotypic impacts of CSF1R deficiencies in humans and model organisms. J Leukoc Biol. 2020;107(2):205–19. 10.1002/JLB.MR0519-143R 31330095

[9] Summers KM, Hume DA. Identification of the macrophage-specific promoter signature in FANTOM5 mouse embryo developmental time course data. J Leukoc Biol. 2017;102(4):1081–92. 10.1189/jlb.1A0417-150RR 28751473

[10] Ginhoux F, Guilliams M. Tissue-Resident Macrophage Ontogeny and Homeostasis. Immunity. 2016;44(3):439–49. 10.1016/j.immuni.2016.02.024 26982352

[11] Liu Z, Gu Y, Chakarov S, Bleriot C, Kwok I, Chen X, et al Fate Mapping via Ms4a3-Expression History Traces Monocyte-Derived Cells. Cell. 2019;178(6):1509–25 e19. 10.1016/j.cell.2019.08.009 31491389

[12] Yona S, Kim KW, Wolf Y, Mildner A, Varol D, Breker M, et al Fate mapping reveals origins and dynamics of monocytes and tissue macrophages under homeostasis. Immunity. 2013;38(1):79–91. 10.1016/j.immuni.2012.12.001 23273845 PMC3908543

[13] Jenkins SJ, Hume DA. Homeostasis in the mononuclear phagocyte system. Trends Immunol. 2014;35(8):358–67. 10.1016/j.it.2014.06.006 25047416

[14] Bonnardel J, Guilliams M. Developmental control of macrophage function. Curr Opin Immunol. 2018;50:64–74. 10.1016/j.coi.2017.12.001 29247852

[15] Gomez Perdiguero E, Klapproth K, Schulz C, Busch K, Azzoni E, Crozet L, et al Tissue-resident macrophages originate from yolk-sac-derived erythro-myeloid progenitors. Nature. 2015;518(7540):547–51. 10.1038/nature13989 25470051 PMC5997177

[16] Mass E, Ballesteros I, Farlik M, Halbritter F, Gunther P, Crozet L, et al Specification of tissue-resident macrophages during organogenesis. Science. 2016;353(6304): aaf4238 10.1126/science.aaf4238 27492475 PMC5066309

[17] Rojo R, Raper A, Ozdemir DD, Lefevre L, Grabert K, Wollscheid-Lengeling E, et al Deletion of a Csf1r enhancer selectively impacts CSF1R expression and development of tissue macrophage populations. Nat Commun. 2019;10(1):3215 10.1038/s41467-019-11053-8 31324781 PMC6642117

[18] Henson PM, Hume DA. Apoptotic cell removal in development and tissue homeostasis. Trends Immunol. 2006;27(5):244–50. 10.1016/j.it.2006.03.005 16584921

[19] Hume DA. Macrophages as APC and the dendritic cell myth. J Immunol. 2008;181(9):5829–35. 10.4049/jimmunol.181.9.5829 18941170

[20] Jakubzick CV, Randolph GJ, Henson PM. Monocyte differentiation and antigen-presenting functions. Nat Rev Immunol. 2017;17(6):349–62. 10.1038/nri.2017.28 28436425

[21] Guilliams M, Ginhoux F, Jakubzick C, Naik SH, Onai N, Schraml BU, et al Dendritic cells, monocytes and macrophages: a unified nomenclature based on ontogeny. Nat Rev Immunol. 2014;14(8):571–8. 10.1038/nri3712 25033907 PMC4638219

[22] Sichien D, Lambrecht BN, Guilliams M, Scott CL. Development of conventional dendritic cells: from common bone marrow progenitors to multiple subsets in peripheral tissues. Mucosal Immunol. 2017;10(4):831–44. 10.1038/mi.2017.8 28198365

[23] Gordon S, Pluddemann A. Tissue macrophages: heterogeneity and functions. BMC Biol. 2017;15(1):53 10.1186/s12915-017-0392-4 28662662 PMC5492929

[24] Hume DA. Differentiation and heterogeneity in the mononuclear phagocyte system. Mucosal Immunol. 2008;1(6):432–41. 10.1038/mi.2008.36 19079210

[25] Hume DA. Applications of myeloid-specific promoters in transgenic mice support in vivo imaging and functional genomics but do not support the concept of distinct macrophage and dendritic cell lineages or roles in immunity. J Leukoc Biol. 2011;89(4):525–38. 10.1189/jlb.0810472 21169519

[26] Bassler K, Schulte-Schrepping J, Warnat-Herresthal S, Aschenbrenner AC, Schultze JL. The Myeloid Cell Compartment-Cell by Cell. Annu Rev Immunol. 2019;37:269–93. 10.1146/annurev-immunol-042718-041728 30649988

[27] Chakarov S, Lim HY, Tan L, Lim SY, See P, Lum J, et al Two distinct interstitial macrophage populations coexist across tissues in specific subtissular niches. Science. 2019;363(6432): eeau0964 10.1126/science.aau0964 30872492

[28] Gautier EL, Shay T, Miller J, Greter M, Jakubzick C, Ivanov S, et al Gene-expression profiles and transcriptional regulatory pathways that underlie the identity and diversity of mouse tissue macrophages. Nat Immunol. 2012;13(11):1118–28. 10.1038/ni.2419 23023392 PMC3558276

[29] Miller JC, Brown BD, Shay T, Gautier EL, Jojic V, Cohain A, et al Deciphering the transcriptional network of the dendritic cell lineage. Nat Immunol. 2012;13(9):888–99. 10.1038/ni.2370 22797772 PMC3985403

[30] Hume DA. The Many Alternative Faces of Macrophage Activation. Front Immunol. 2015;6:370 10.3389/fimmu.2015.00370 26257737 PMC4510422

[31] Murray PJ, Allen JE, Biswas SK, Fisher EA, Gilroy DW, Goerdt S, et al Macrophage activation and polarization: nomenclature and experimental guidelines. Immunity. 2014;41(1):14–20. 10.1016/j.immuni.2014.06.008 25035950 PMC4123412

[32] Sakai M, Troutman TD, Seidman JS, Ouyang Z, Spann NJ, Abe Y, et al Liver-Derived Signals Sequentially Reprogram Myeloid Enhancers to Initiate and Maintain Kupffer Cell Identity. Immunity. 2019;51(4):655–70 e8. 10.1016/j.immuni.2019.09.002 31587991 PMC6800814

[33] Kaur S, Raggatt LJ, Batoon L, Hume DA, Levesque JP, Pettit AR. Role of bone marrow macrophages in controlling homeostasis and repair in bone and bone marrow niches. Semin Cell Dev Biol. 2017;61:12–21. 10.1016/j.semcdb.2016.08.009 27521519

[34] MacDonald KP, Palmer JS, Cronau S, Seppanen E, Olver S, Raffelt NC, et al An antibody against the colony-stimulating factor 1 receptor depletes the resident subset of monocytes and tissue- and tumor-associated macrophages but does not inhibit inflammation. Blood. 2010;116(19):3955–63. 10.1182/blood-2010-02-266296 20682855

[35] Geissmann F, Manz MG, Jung S, Sieweke MH, Merad M, Ley K. Development of monocytes, macrophages, and dendritic cells. Science. 2010;327(5966):656–61. 10.1126/science.1178331 20133564 PMC2887389

[36] De Schepper S, Verheijden S, Aguilera-Lizarraga J, Viola MF, Boesmans W, Stakenborg N, et al Self-Maintaining Gut Macrophages Are Essential for Intestinal Homeostasis. Cell. 2019;176(3):676 10.1016/j.cell.2019.01.010 30682373

[37] Shaw TN, Houston SA, Wemyss K, Bridgeman HM, Barbera TA, Zangerle-Murray T, et al Tissue-resident macrophages in the intestine are long lived and defined by Tim-4 and CD4 expression. J Exp Med. 2018;215(6):1507–18. 10.1084/jem.20180019 29789388 PMC5987925

[38] Hume DA, Freeman TC. Transcriptomic analysis of mononuclear phagocyte differentiation and activation. Immunol Rev. 2014;262(1):74–84. 10.1111/imr.12211 25319328

[39] Hume DA, Summers KM, Raza S, Baillie JK, Freeman TC. Functional clustering and lineage markers: insights into cellular differentiation and gene function from large-scale microarray studies of purified primary cell populations. Genomics. 2010;95(6):328–38. 10.1016/j.ygeno.2010.03.002 20211243

[40] Joshi A, Pooley C, Freeman TC, Lennartsson A, Babina M, Schmidl C, et al Technical Advance: Transcription factor, promoter, and enhancer utilization in human myeloid cells. J Leukoc Biol. 2015;97(5):985–95. 10.1189/jlb.6TA1014-477RR 25717144 PMC4398258

[41] Mabbott NA, Baillie JK, Brown H, Freeman TC, Hume DA. An expression atlas of human primary cells: inference of gene function from coexpression networks. BMC Genomics. 2013;14:632 10.1186/1471-2164-14-632 24053356 PMC3849585

[42] Mabbott NA, Kenneth Baillie J, Hume DA, Freeman TC. Meta-analysis of lineage-specific gene expression signatures in mouse leukocyte populations. Immunobiology. 2010;215(9–10):724–36. 10.1016/j.imbio.2010.05.012 20580463

[43] Doig TN, Hume DA, Theocharidis T, Goodlad JR, Gregory CD, Freeman TC. Coexpression analysis of large cancer datasets provides insight into the cellular phenotypes of the tumour microenvironment. BMC Genomics. 2013;14:469 10.1186/1471-2164-14-469 23845084 PMC3721986

[44] Hume DA, Mabbott N, Raza S, Freeman TC. Can DCs be distinguished from macrophages by molecular signatures? Nat Immunol. 2013;14(3):187–9. 10.1038/ni.2516 23416664

[45] Bush SJ, Freem L, MacCallum AJ, O’Dell J, Wu C, Afrasiabi C, et al Combination of novel and public RNA-seq datasets to generate an mRNA expression atlas for the domestic chicken. BMC Genomics. 2018;19(1):594 10.1186/s12864-018-4972-7 30086717 PMC6081845

[46] Summers K, Bush S, Wu C, Su A, Muriuki C, Clark E, et al Functional annotation of the transcriptome of the pig, sus scrofa, based upon network analysis of an RNAseq transcriptional atlas. Frontiers Genetics. 2020;10:355 10.3389/fgene.2019.01355 PMC7034361 32117413

[47] Clark EL, Bush SJ, McCulloch MEB, Farquhar IL, Young R, Lefevre L, et al A high resolution atlas of gene expression in the domestic sheep (Ovis aries). PLoS Genet. 2017;13(9):e1006997 10.1371/journal.pgen.1006997 28915238 PMC5626511

[48] Forrest ARR, Kawaji H, Rehli M, Baillie JK, de Hoon MJ, Haberle V, et al A promoter-level mammalian expression atlas. Nature. 2014;507(7493):462–70. 10.1038/nature13182 24670764 PMC4529748

[49] Young R, Lefevre L, Bush SJ, Joshi A, Singh SH, Jadhav SK, et al A Gene Expression Atlas of the Domestic Water Buffalo (Bubalus bubalis). Front Genet. 2019;10:668 10.3389/fgene.2019.00668 31428126 PMC6689995

[50] Bray NL, Pimentel H, Melsted P, Pachter L. Near-optimal probabilistic RNA-seq quantification. Nat Biotechnol. 2016;34(5):525–7. 10.1038/nbt.3519 27043002

[51] Fujiu K, Shibata M, Nakayama Y, Ogata F, Matsumoto S, Noshita K, et al A heart-brain-kidney network controls adaptation to cardiac stress through tissue macrophage activation. Nat Med. 2017;23(5):611–22. 10.1038/nm.4326 28394333

[52] Gibbings SL, Thomas SM, Atif SM, McCubbrey AL, Desch AN, Danhorn T, et al Three Unique Interstitial Macrophages in the Murine Lung at Steady State. Am J Respir Cell Mol Biol. 2017;57(1):66–76. 10.1165/rcmb.2016-0361OC 28257233 PMC5516280

[53] Wolf Y, Boura-Halfon S, Cortese N, Haimon Z, Sar Shalom H, Kuperman Y, et al Brown-adipose-tissue macrophages control tissue innervation and homeostatic energy expenditure. Nat Immunol. 2017;18(6):665–74. 10.1038/ni.3746 28459435 PMC5438596

[54] Puranik AS, Leaf IA, Jensen MA, Hedayat AF, Saad A, Kim KW, et al Kidney-resident macrophages promote a proangiogenic environment in the normal and chronically ischemic mouse kidney. Sci Rep. 2018;8(1):13948 10.1038/s41598-018-31887-4 30224726 PMC6141464

[55] Shemer A, Grozovski J, Tay TL, Tao J, Volaski A, Suss P, et al Engrafted parenchymal brain macrophages differ from microglia in transcriptome, chromatin landscape and response to challenge. Nat Commun. 2018;9(1):5206 10.1038/s41467-018-07548-5 30523248 PMC6284018

[56] Li Q, Cheng Z, Zhou L, Darmanis S, Neff NF, Okamoto J, et al Developmental Heterogeneity of Microglia and Brain Myeloid Cells Revealed by Deep Single-Cell RNA Sequencing. Neuron. 2019;101(2):207–23 e10. 10.1016/j.neuron.2018.12.006 30606613 PMC6336504

[57] Li W, Wang Y, Zhao H, Zhang H, Xu Y, Wang S, et al Identification and transcriptome analysis of erythroblastic island macrophages. Blood. 2019;134(5):480–91. 10.1182/blood.2019000430 31101625 PMC6676133

[58] Rauschmeier R, Gustafsson C, Reinhardt A, N AG, Tortola L, Cansever D, et al Bhlhe40 and Bhlhe41 transcription factors regulate alveolar macrophage self-renewal and identity. EMBO J. 2019;38(19):e101233 10.15252/embj.2018101233 31414712 PMC6769426

[59] Van Hove H, Martens L, Scheyltjens I, De Vlaminck K, Pombo Antunes AR, De Prijck S, et al A single-cell atlas of mouse brain macrophages reveals unique transcriptional identities shaped by ontogeny and tissue environment. Nat Neurosci. 2019;22(6):1021–35. 10.1038/s41593-019-0393-4 31061494

[60] Ying W, Lee YS, Dong Y, Seidman JS, Yang M, Isaac R, et al Expansion of Islet-Resident Macrophages Leads to Inflammation Affecting beta Cell Proliferation and Function in Obesity. Cell Metab. 2019;29(2):457–74 e5. 10.1016/j.cmet.2018.12.003 30595478 PMC6701710

[61] Pirzgalska RM, Seixas E, Seidman JS, Link VM, Sanchez NM, Mahu I, et al Sympathetic neuron-associated macrophages contribute to obesity by importing and metabolizing norepinephrine. Nat Med. 2017;23(11):1309–18. 10.1038/nm.4422 29035364 PMC7104364

[62] Capucha T, Mizraji G, Segev H, Blecher-Gonen R, Winter D, Khalaileh A, et al Distinct Murine Mucosal Langerhans Cell Subsets Develop from Pre-dendritic Cells and Monocytes. Immunity. 2015;43(2):369–81. 10.1016/j.immuni.2015.06.017 26231115

[63] Jaitin DA, Adlung L, Thaiss CA, Weiner A, Li B, Descamps H, et al Lipid-Associated Macrophages Control Metabolic Homeostasis in a Trem2-Dependent Manner. Cell. 2019;178(3):686–98 e14. 10.1016/j.cell.2019.05.054 31257031 PMC7068689

[64] Thion MS, Low D, Silvin A, Chen J, Grisel P, Schulte-Schrepping J, et al Microbiome Influences Prenatal and Adult Microglia in a Sex-Specific Manner. Cell. 2018;172(3):500–16 e16. 10.1016/j.cell.2017.11.042 29275859 PMC5786503

[65] N AG, Quintana JA, Garcia-Silva S, Mazariegos M, Gonzalez de la Aleja A, Nicolas-Avila JA, et al Phagocytosis imprints heterogeneity in tissue-resident macrophages. J Exp Med. 2017;214(5):1281–96. 10.1084/jem.20161375 28432199 PMC5413334

[66] Mildner A, Schonheit J, Giladi A, David E, Lara-Astiaso D, Lorenzo-Vivas E, et al Genomic Characterization of Murine Monocytes Reveals C/EBPbeta Transcription Factor Dependence of Ly6C(-) Cells. Immunity. 2017;46(5):849–62 e7. 10.1016/j.immuni.2017.04.018 28514690

[67] Brown CC, Gudjonson H, Pritykin Y, Deep D, Lavallee VP, Mendoza A, et al Transcriptional Basis of Mouse and Human Dendritic Cell Heterogeneity. Cell. 2019;179(4):846–63 e24. 10.1016/j.cell.2019.09.035 31668803 PMC6838684

[68] Stock AT, Collins N, Smyth GK, Hu Y, Hansen JA, D’Silva DB, et al The Selective Expansion and Targeted Accumulation of Bone Marrow-Derived Macrophages Drive Cardiac Vasculitis. J Immunol. 2019;202(11):3282–96. 10.4049/jimmunol.1900071 31004011

[69] Gross-Vered M, Trzebanski S, Shemer A, Bernshtein B, Curato C, Stelzer G, et al Defining murine monocyte differentiation into colonic and ileal macrophages. Elife. 2020;9: e49998 10.7554/eLife.49998 31916932 PMC6952180

[70] Salei N, Rambichler S, Salvermoser J, Papaioannou NE, Schuchert R, Pakalniskyte D, et al The Kidney Contains Ontogenetically Distinct Dendritic Cell and Macrophage Subtypes throughout Development That Differ in Their Inflammatory Properties. J Am Soc Nephrol. 2020;31(2):257–78. 10.1681/ASN.2019040419 31932472 PMC7003301

[71] Aitchison L, Corradi N, Latham PE. Zipf’s Law Arises Naturally When There Are Underlying, Unobserved Variables. PLoS Comput Biol. 2016;12(12):e1005110 10.1371/journal.pcbi.1005110 27997544 PMC5172588

[72] Ueda HR, Hayashi S, Matsuyama S, Yomo T, Hashimoto S, Kay SA, et al Universality and flexibility in gene expression from bacteria to human. Proc Natl Acad Sci U S A. 2004;101(11):3765–9. 10.1073/pnas.0306244101 14999098 PMC374318

[73] Stephens AS, Stephens SR, Morrison NA. Internal control genes for quantitative RT-PCR expression analysis in mouse osteoblasts, osteoclasts and macrophages. BMC Res Notes. 2011;4:410 10.1186/1756-0500-4-410 21996334 PMC3204251

[74] Hawley CA, Rojo R, Raper A, Sauter KA, Lisowski ZM, Grabert K, et al Csf1r-mApple Transgene Expression and Ligand Binding In Vivo Reveal Dynamics of CSF1R Expression within the Mononuclear Phagocyte System. J Immunol. 2018;200(6):2209–23. 10.4049/jimmunol.1701488 29440354 PMC5834790

[75] Sasmono RT, Oceandy D, Pollard JW, Tong W, Pavli P, Wainwright BJ, et al A macrophage colony-stimulating factor receptor-green fluorescent protein transgene is expressed throughout the mononuclear phagocyte system of the mouse. Blood. 2003;101(3):1155–63. 10.1182/blood-2002-02-0569 12393599

[76] Schyns J, Bai Q, Ruscitti C, Radermecker C, De Schepper S, Chakarov S, et al Non-classical tissue monocytes and two functionally distinct populations of interstitial macrophages populate the mouse lung. Nat Commun. 2019;10(1):3964 10.1038/s41467-019-11843-0 31481690 PMC6722135

[77] Gal-Oz ST, Maier B, Yoshida H, Seddu K, Elbaz N, Czysz C, et al ImmGen report: sexual dimorphism in the immune system transcriptome. Nat Commun. 2019;10(1):4295 10.1038/s41467-019-12348-6 31541153 PMC6754408

[78] Bonnardel J, T’Jonck W, Gaublomme D, Browaeys R, Scott CL, Martens L, et al Stellate Cells, Hepatocytes, and Endothelial Cells Imprint the Kupffer Cell Identity on Monocytes Colonizing the Liver Macrophage Niche. Immunity. 2019;51(4):638–54 e9. 10.1016/j.immuni.2019.08.017 31561945 PMC6876284

[79] Hume DA, Summers KM, Rehli M. Transcriptional Regulation and Macrophage Differentiation. Microbiol. Spectr. 2016;4(3). 10.1128/microbiolspec.MCHD-0024-2015 27337479

[80] Rojo R, Pridans C, Langlais D, Hume DA. Transcriptional mechanisms that control expression of the macrophage colony-stimulating factor receptor locus. Clin Sci (Lond). 2017;131(16):2161–82. 28760770 10.1042/CS20170238

[81] Jubb AW, Young RS, Hume DA, Bickmore WA. Enhancer Turnover Is Associated with a Divergent Transcriptional Response to Glucocorticoid in Mouse and Human Macrophages. J Immunol. 2016;196(2):813–22. 10.4049/jimmunol.1502009 26663721 PMC4707550

[82] Audesse AJ, Dhakal S, Hassell LA, Gardell Z, Nemtsova Y, Webb AE. FOXO3 directly regulates an autophagy network to functionally regulate proteostasis in adult neural stem cells. PLoS Genet. 2019;15(4):e1008097 10.1371/journal.pgen.1008097 30973875 PMC6478346

[83] Chen Y, Wu J, Liang G, Geng G, Zhao F, Yin P, et al CHK2-FOXK axis promotes transcriptional control of autophagy programs. Sci Adv. 2020;6(1):eaax5819 10.1126/sciadv.aax5819 31911943 PMC6938702

[84] Schaffner I, Minakaki G, Khan MA, Balta EA, Schlotzer-Schrehardt U, Schwarz TJ, et al FoxO Function Is Essential for Maintenance of Autophagic Flux and Neuronal Morphogenesis in Adult Neurogenesis. Neuron. 2018;99(6):1188–203 e6. 10.1016/j.neuron.2018.08.017 30197237 PMC6186958

[85] Choi S, You S, Kim D, Choi SY, Kwon HM, Kim HS, et al Transcription factor NFAT5 promotes macrophage survival in rheumatoid arthritis. J Clin Invest. 2017;127(3):954–69. 10.1172/JCI87880 28192374 PMC5330733

[86] Roberts TL, Idris A, Dunn JA, Kelly GM, Burnton CM, Hodgson S, et al HIN-200 proteins regulate caspase activation in response to foreign cytoplasmic DNA. Science. 2009;323(5917):1057–60. 10.1126/science.1169841 19131592

[87] Briard B, Place DE, Kanneganti TD. DNA Sensing in the Innate Immune Response. Physiology (Bethesda). 2020;35(2):112–24. 32027562 10.1152/physiol.00022.2019 PMC7276919

[88] Zhao Y, Shi J, Shi X, Wang Y, Wang F, Shao F. Genetic functions of the NAIP family of inflammasome receptors for bacterial ligands in mice. J Exp Med. 2016;213(5):647–56. 10.1084/jem.20160006 27114610 PMC4854738

[89] Pridans C, Raper A, Davis GM, Alves J, Sauter KA, Lefevre L, et al Pleiotropic Impacts of Macrophage and Microglial Deficiency on Development in Rats with Targeted Mutation of the Csf1r Locus. J Immunol. 2018;201(9):2683–99. 10.4049/jimmunol.1701783 30249809 PMC6196293

[90] Giotti B, Chen SH, Barnett MW, Regan T, Ly T, Wiemann S, et al Assembly of a parts list of the human mitotic cell cycle machinery. J Mol Cell Biol. 2019;11(8):703–18. 10.1093/jmcb/mjy063 30452682 PMC6788831

[91] Aoki M, Aoki H, Ramanathan R, Hait NC, Takabe K. Sphingosine-1-Phosphate Signaling in Immune Cells and Inflammation: Roles and Therapeutic Potential. Mediators Inflamm. 2016;2016:8606878 10.1155/2016/8606878 26966342 PMC4761394

[92] Weichand B, Popp R, Dziumbla S, Mora J, Strack E, Elwakeel E, et al S1PR1 on tumor-associated macrophages promotes lymphangiogenesis and metastasis via NLRP3/IL-1beta. J Exp Med. 2017;214(9):2695–713. 10.1084/jem.20160392 28739604 PMC5584110

[93] Dutta B, Arya RK, Goswami R, Alharbi MO, Sharma S, Rahaman SO. Role of macrophage TRPV4 in inflammation. Lab Invest. 2020;100(2):178–85. 10.1038/s41374-019-0334-6 31645630 PMC7261496

[94] Morty RE, Kuebler WM. TRPV4: an exciting new target to promote alveolocapillary barrier function. Am J Physiol Lung Cell Mol Physiol. 2014;307(11):L817–21. 10.1152/ajplung.00254.2014 25281637

[95] Issitt T, Bosseboeuf E, De Winter N, Dufton N, Gestri G, Senatore V, et al Neuropilin-1 Controls Endothelial Homeostasis by Regulating Mitochondrial Function and Iron-Dependent Oxidative Stress. iScience. 2019;11:205–23. 10.1016/j.isci.2018.12.005 30623799 PMC6327076

[96] Luo B, Gan W, Liu Z, Shen Z, Wang J, Shi R, et al Erythropoeitin Signaling in Macrophages Promotes Dying Cell Clearance and Immune Tolerance. Immunity. 2016;44(2):287–302. 10.1016/j.immuni.2016.01.002 26872696

[97] Hardbower DM, Singh K, Asim M, Verriere TG, Olivares-Villagomez D, Barry DP, et al EGFR regulates macrophage activation and function in bacterial infection. J Clin Invest. 2016;126(9):3296–312. 10.1172/JCI83585 27482886 PMC5004944

[98] Ivanov S, Randolph GJ. Myeloid cells pave the way for lymphatic system development and maintenance. Pflugers Arch. 2017;469(3–4):465–72. 10.1007/s00424-017-1951-9 28220247 PMC5467692

[99] Sanin DE, Matsushita M, Klein Geltink RI, Grzes KM, van Teijlingen Bakker N, Corrado M, et al Mitochondrial Membrane Potential Regulates Nuclear Gene Expression in Macrophages Exposed to Prostaglandin E2. Immunity. 2018;49(6):1021–33 e6. 10.1016/j.immuni.2018.10.011 30566880 PMC7271981

[100] Yu J, Zanotti S, Schilling L, Canalis E. Nuclear factor of activated T cells 2 is required for osteoclast differentiation and function in vitro but not in vivo. J Cell Biochem. 2018;119(11):9334–45. 10.1002/jcb.27212 30010214 PMC6195439

[101] Van den Bossche J, Malissen B, Mantovani A, De Baetselier P, Van Ginderachter JA. Regulation and function of the E-cadherin/catenin complex in cells of the monocyte-macrophage lineage and DCs. Blood. 2012;119(7):1623–33. 10.1182/blood-2011-10-384289 22174153

[102] Spadaro O, Camell CD, Bosurgi L, Nguyen KY, Youm YH, Rothlin CV, et al IGF1 Shapes Macrophage Activation in Response to Immunometabolic Challenge. Cell Rep. 2017;19(2):225–34. 10.1016/j.celrep.2017.03.046 28402847 PMC5513500

[103] Maridas DE, DeMambro VE, Le PT, Mohan S, Rosen CJ. IGFBP4 Is Required for Adipogenesis and Influences the Distribution of Adipose Depots. Endocrinology. 2017;158(10):3488–500. 10.1210/en.2017-00248 28938423 PMC5659704

[104] Maridas DE, DeMambro VE, Le PT, Nagano K, Baron R, Mohan S, et al IGFBP-4 regulates adult skeletal growth in a sex-specific manner. J Endocrinol. 2017;233(1):131–44. 10.1530/JOE-16-0673 28184001 PMC5425953

[105] Hume DA. The mononuclear phagocyte system. Curr Opin Immunol. 2006;18(1):49–53. 10.1016/j.coi.2005.11.008 16338128

[106] Schulz C, Gomez Perdiguero E, Chorro L, Szabo-Rogers H, Cagnard N, Kierdorf K, et al A lineage of myeloid cells independent of Myb and hematopoietic stem cells. Science. 2012;336(6077):86–90. 10.1126/science.1219179 22442384

[107] Bain CC, Schridde A. Origin, Differentiation, and Function of Intestinal Macrophages. Front Immunol. 2018;9:2733 10.3389/fimmu.2018.02733 30538701 PMC6277706

[108] Briseno CG, Haldar M, Kretzer NM, Wu X, Theisen DJ, Kc W, et al Distinct Transcriptional Programs Control Cross-Priming in Classical and Monocyte-Derived Dendritic Cells. Cell Rep. 2016;15(11):2462–74. 10.1016/j.celrep.2016.05.025 27264183 PMC4941620

[109] Waddell LA, Lefevre L, Bush SJ, Raper A, Young R, Lisowski ZM, et al ADGRE1 (EMR1, F4/80) Is a Rapidly-Evolving Gene Expressed in Mammalian Monocyte-Macrophages. Front Immunol. 2018;9:2246 10.3389/fimmu.2018.02246 30327653 PMC6174849

[110] Scott CL, Guilliams M. The role of Kupffer cells in hepatic iron and lipid metabolism. J Hepatol. 2018;69(5):1197–9. 10.1016/j.jhep.2018.02.013 30001821 PMC7611037

[111] Ma F, Liu SY, Razani B, Arora N, Li B, Kagechika H, et al Retinoid X receptor alpha attenuates host antiviral response by suppressing type I interferon. Nat Commun. 2014;5:5494 10.1038/ncomms6494 25417649 PMC4380327

[112] van der Spek AH, Fliers E, Boelen A. Thyroid hormone metabolism in innate immune cells. J Endocrinol. 2017;232(2):R67–R81. 10.1530/JOE-16-0462 27852725

[113] Roussel-Gervais A, Naciri I, Kirsh O, Kasprzyk L, Velasco G, Grillo G, et al Loss of the Methyl-CpG-Binding Protein ZBTB4 Alters Mitotic Checkpoint, Increases Aneuploidy, and Promotes Tumorigenesis. Cancer Res. 2017;77(1):62–73. 10.1158/0008-5472.CAN-16-1181 27815388

[114] Haldar M, Kohyama M, So AY, Kc W, Wu X, Briseno CG, et al Heme-mediated SPI-C induction promotes monocyte differentiation into iron-recycling macrophages. Cell. 2014;156(6):1223–34. 10.1016/j.cell.2014.01.069 24630724 PMC4010949

[115] Kohyama M, Ise W, Edelson BT, Wilker PR, Hildner K, Mejia C, et al Role for Spi-C in the development of red pulp macrophages and splenic iron homeostasis. Nature. 2009;457(7227):318–21. 10.1038/nature07472 19037245 PMC2756102

[116] Bain CC, Hawley CA, Garner H, Scott CL, Schridde A, Steers NJ, et al Long-lived self-renewing bone marrow-derived macrophages displace embryo-derived cells to inhabit adult serous cavities. Nat Commun. 2016;7:11852. 10.1038/ncomms11852 PMC4910019 27292029

[117] Okabe Y, Medzhitov R. Tissue-specific signals control reversible program of localization and functional polarization of macrophages. Cell. 2014;157(4):832–44. 10.1016/j.cell.2014.04.016 24792964 PMC4137874

[118] Costelloe EO, Stacey KJ, Antalis TM, Hume DA. Regulation of the plasminogen activator inhibitor-2 (PAI-2) gene in murine macrophages. Demonstration of a novel pattern of responsiveness to bacterial endotoxin. J Leukoc Biol. 1999;66(1):172–82. 10.1002/jlb.66.1.172 10411006

[119] Schroder WA, Hirata TD, Le TT, Gardner J, Boyle GM, Ellis J, et al SerpinB2 inhibits migration and promotes a resolution phase signature in large peritoneal macrophages. Sci Rep. 2019;9(1):12421 10.1038/s41598-019-48741-w 31455834 PMC6712035

[120] Doebel T, Voisin B, Nagao K. Langerhans Cells—The Macrophage in Dendritic Cell Clothing. Trends Immunol. 2017;38(11):817–28. 10.1016/j.it.2017.06.008 28720426

[121] Hung LY, Johnson JL, Ji Y, Christian DA, Herbine KR, Pastore CF, et al Cell-Intrinsic Wnt4 Influences Conventional Dendritic Cell Fate Determination to Suppress Type 2 Immunity. J Immunol. 2019;203(2):511–9. 10.4049/jimmunol.1900363 31175162 PMC6615948

[122] Sehgal A, Donaldson DS, Pridans C, Sauter KA, Hume DA, Mabbott NA. The role of CSF1R-dependent macrophages in control of the intestinal stem-cell niche. Nat Commun. 2018;9(1):1272 10.1038/s41467-018-03638-6 29593242 PMC5871851

[123] Shang Y, Coppo M, He T, Ning F, Yu L, Kang L, et al The transcriptional repressor Hes1 attenuates inflammation by regulating transcription elongation. Nat Immunol. 2016;17(8):930–7. 10.1038/ni.3486 27322654 PMC4955730

[124] Zhang X, Li X, Ning F, Shang Y, Hu X. TLE4 acts as a corepressor of Hes1 to inhibit inflammatory responses in macrophages. Protein Cell. 2019;10(4):300–5. 10.1007/s13238-018-0554-3 29869113 PMC6418302

[125] Schridde A, Bain CC, Mayer JU, Montgomery J, Pollet E, Denecke B, et al Tissue-specific differentiation of colonic macrophages requires TGFbeta receptor-mediated signaling. Mucosal Immunol. 2017;10(6):1387–99. 10.1038/mi.2016.142 28145440 PMC5417360

[126] Morris DL, Singer K, Lumeng CN. Adipose tissue macrophages: phenotypic plasticity and diversity in lean and obese states. Curr Opin Clin Nutr Metab Care. 2011;14(4):341–6. 10.1097/MCO.0b013e328347970b 21587064 PMC4690541

[127] Lee MR, Lim CJ, Lee YH, Park JG, Sonn SK, Lee MN, et al The adipokine Retnla modulates cholesterol homeostasis in hyperlipidemic mice. Nat Commun. 2014;5:4410 10.1038/ncomms5410 25022542

[128] Kumamoto Y, Camporez JPG, Jurczak MJ, Shanabrough M, Horvath T, Shulman GI, et al CD301b(+) Mononuclear Phagocytes Maintain Positive Energy Balance through Secretion of Resistin-like Molecule Alpha. Immunity. 2016;45(3):583–96. 10.1016/j.immuni.2016.08.002 27566941 PMC5033704

[129] Fujita K, Chakarov S, Kobayashi T, Sakamoto K, Voisin B, Duan K, et al Cell-autonomous FLT3L shedding via ADAM10 mediates conventional dendritic cell development in mouse spleen. Proc Natl Acad Sci U S A. 2019;116(29):14714–23. 10.1073/pnas.1818907116 31262819 PMC6642353

[130] Pool L, Rivollier A, Agace WW. Deletion of IRF4 in Dendritic Cells Leads to Delayed Onset of T Cell-Dependent Colitis. J Immunol. 2020;204(4):1047–55. 10.4049/jimmunol.1900775 31900340

[131] Anderson DA 3rd, Murphy KM, Briseno CG. Development, Diversity, and Function of Dendritic Cells in Mouse and Human. Cold Spring Harb Perspect Biol. 2018;10(11): a028613 10.1101/cshperspect.a028613 28963110 PMC6211386

[132] Forster R, Davalos-Misslitz AC, Rot A. CCR7 and its ligands: balancing immunity and tolerance. Nat Rev Immunol. 2008;8(5):362–71. 10.1038/nri2297 18379575

[133] Hildner K, Edelson BT, Purtha WE, Diamond M, Matsushita H, Kohyama M, et al Batf3 deficiency reveals a critical role for CD8alpha+ dendritic cells in cytotoxic T cell immunity. Science. 2008;322(5904):1097–100. 10.1126/science.1164206 19008445 PMC2756611

[134] Bagadia P, Huang X, Liu TT, Durai V, Grajales-Reyes GE, Nitschke M, et al An Nfil3-Zeb2-Id2 pathway imposes Irf8 enhancer switching during cDC1 development. Nat Immunol. 2019;20(9):1174–85. 10.1038/s41590-019-0449-3 31406377 PMC6707889

[135] Satpathy AT, Kc W, Albring JC, Edelson BT, Kretzer NM, Bhattacharya D, et al Zbtb46 expression distinguishes classical dendritic cells and their committed progenitors from other immune lineages. J Exp Med. 2012;209(6):1135–52. 10.1084/jem.20120030 22615127 PMC3371733

[136] Nagae M, Ikeda A, Hanashima S, Kojima T, Matsumoto N, Yamamoto K, et al Crystal structure of human dendritic cell inhibitory receptor C-type lectin domain reveals the binding mode with N-glycan. FEBS Lett. 2016;590(8):1280–8. 10.1002/1873-3468.12162 27015765

[137] Troegeler A, Mercier I, Cougoule C, Pietretti D, Colom A, Duval C, et al C-type lectin receptor DCIR modulates immunity to tuberculosis by sustaining type I interferon signaling in dendritic cells. Proc Natl Acad Sci U S A. 2017;114(4):E540–E9. 10.1073/pnas.1613254114 28069953 PMC5278472

[138] Uto T, Fukaya T, Takagi H, Arimura K, Nakamura T, Kojima N, et al Clec4A4 is a regulatory receptor for dendritic cells that impairs inflammation and T-cell immunity. Nat Commun. 2016;7:11273 10.1038/ncomms11273 27068492 PMC4832068

[139] Wells CA, Salvage-Jones JA, Li X, Hitchens K, Butcher S, Murray RZ, et al The macrophage-inducible C-type lectin, mincle, is an essential component of the innate immune response to Candida albicans. J Immunol. 2008;180(11):7404–13. 10.4049/jimmunol.180.11.7404 18490740

[140] Link VM, Duttke SH, Chun HB, Holtman IR, Westin E, Hoeksema MA, et al Analysis of Genetically Diverse Macrophages Reveals Local and Domain-wide Mechanisms that Control Transcription Factor Binding and Function. Cell. 2018;173(7):1796–809 e17. 10.1016/j.cell.2018.04.018 29779944 PMC6003872

[141] Tamura A, Hirai H, Yokota A, Kamio N, Sato A, Shoji T, et al C/EBPbeta is required for survival of Ly6C(-) monocytes. Blood. 2017;130(16):1809–18. 10.1182/blood-2017-03-772962 28807982 PMC5649551

[142] Bornstein C, Winter D, Barnett-Itzhaki Z, David E, Kadri S, Garber M, et al A negative feedback loop of transcription factors specifies alternative dendritic cell chromatin States. Mol Cell. 2014;56(6):749–62. 10.1016/j.molcel.2014.10.014 25453760 PMC4412443

[143] Satpathy AT, Wu X, Albring JC, Murphy KM. Re(de)fining the dendritic cell lineage. Nat Immunol. 2012;13(12):1145–54. 10.1038/ni.2467 23160217 PMC3644874

[144] Wu X, Briseno CG, Durai V, Albring JC, Haldar M, Bagadia P, et al Mafb lineage tracing to distinguish macrophages from other immune lineages reveals dual identity of Langerhans cells. J Exp Med. 2016;213(12):2553–65. 10.1084/jem.20160600 27810926 PMC5110021

[145] Papadakis KA, Krempski J, Svingen P, Xiong Y, Sarmento OF, Lomberk GA, et al Kruppel-like factor KLF10 deficiency predisposes to colitis through colonic macrophage dysregulation. Am J Physiol Gastrointest Liver Physiol. 2015;309(11):G900–9. 10.1152/ajpgi.00309.2015 26472224 PMC4669350

[146] van den Brink SC, Sage F, Vertesy A, Spanjaard B, Peterson-Maduro J, Baron CS, et al Single-cell sequencing reveals dissociation-induced gene expression in tissue subpopulations. Nat Methods. 2017;14(10):935–6. 10.1038/nmeth.4437 28960196

[147] Ryan DG, O’Neill LAJ. Krebs Cycle Reborn in Macrophage Immunometabolism. Annu Rev Immunol. 2020 28:289–313 10.1146/annurev-immunol-081619-104850 31986069

[148] Gray EE, Friend S, Suzuki K, Phan TG, Cyster JG. Subcapsular sinus macrophage fragmentation and CD169+ bleb acquisition by closely associated IL-17-committed innate-like lymphocytes. PLoS ONE. 2012;7(6):e38258 10.1371/journal.pone.0038258 22675532 PMC3365896

[149] Lynch RW, Hawley CA, Pellicoro A, Bain CC, Iredale JP, Jenkins SJ. An efficient method to isolate Kupffer cells eliminating endothelial cell contamination and selective bias. J Leukoc Biol. 2018;104:578–586. 10.1002/JLB.1TA0517-169R PMC6175317 29607532

[150] Cummings RJ, Barbet G, Bongers G, Hartmann BM, Gettler K, Muniz L, et al Different tissue phagocytes sample apoptotic cells to direct distinct homeostasis programs. Nature. 2016;539(7630):565–9. 10.1038/nature20138 27828940 PMC5807003

[151] Lambert SA, Jolma A, Campitelli LF, Das PK, Yin Y, Albu M, et al The Human Transcription Factors. Cell. 2018;175(2):598–9. 10.1016/j.cell.2018.09.045 30290144

[152] Anderson DA 3rd, Murphy TL, Eisenman RN, Murphy KM. The MYCL and MXD1 transcription factors regulate the fitness of murine dendritic cells. Proc Natl Acad Sci U S A. 2020;117:4885–4893 10.1073/pnas.1915060117 32071205 PMC7060746

[153] Curi R, de Siqueira Mendes R, de Campos Crispin LA, Norata GD, Sampaio SC, Newsholme P. A past and present overview of macrophage metabolism and functional outcomes. Clin Sci (Lond). 2017;131(12):1329–42. 28592702 10.1042/CS20170220

[154] Liu PS, Wang H, Li X, Chao T, Teav T, Christen S, et al alpha-ketoglutarate orchestrates macrophage activation through metabolic and epigenetic reprogramming. Nat Immunol. 2017;18(9):985–94. 10.1038/ni.3796 28714978

[155] Bhutia YD, Ganapathy V. Glutamine transporters in mammalian cells and their functions in physiology and cancer. Biochim Biophys Acta. 2016;1863(10):2531–9. 10.1016/j.bbamcr.2015.12.017 26724577 PMC4919214

[156] Freemerman AJ, Zhao L, Pingili AK, Teng B, Cozzo AJ, Fuller AM, et al Myeloid Slc2a1-Deficient Murine Model Revealed Macrophage Activation and Metabolic Phenotype Are Fueled by GLUT1. J Immunol. 2019;202(4):1265–86. 10.4049/jimmunol.1800002 30659108 PMC6360258

[157] Fang HY, Hughes R, Murdoch C, Coffelt SB, Biswas SK, Harris AL, et al Hypoxia-inducible factors 1 and 2 are important transcriptional effectors in primary macrophages experiencing hypoxia. Blood. 2009;114(4):844–59. 10.1182/blood-2008-12-195941 19454749 PMC2882173

[158] Johnson AR, Qin Y, Cozzo AJ, Freemerman AJ, Huang MJ, Zhao L, et al Metabolic reprogramming through fatty acid transport protein 1 (FATP1) regulates macrophage inflammatory potential and adipose inflammation. Mol Metab. 2016;5(7):506–26. 10.1016/j.molmet.2016.04.005 27408776 PMC4921943

[159] Zhao L, Cozzo AJ, Johnson AR, Christensen T, Freemerman AJ, Bear JE, et al Lack of myeloid Fatp1 increases atherosclerotic lesion size in Ldlr(-/-) mice. Atherosclerosis. 2017;266:182–9. 10.1016/j.atherosclerosis.2017.10.009 29035781 PMC5705203

[160] Nomura N, Verdon G, Kang HJ, Shimamura T, Nomura Y, Sonoda Y, et al Structure and mechanism of the mammalian fructose transporter GLUT5. Nature. 2015;526(7573):397–401. 10.1038/nature14909 26416735 PMC4618315

[161] Caruana BT, Byrne FL, Knights AJ, Quinlan KGR, Hoehn KL. Characterization of Glucose Transporter 6 in Lipopolysaccharide-Induced Bone Marrow-Derived Macrophage Function. J Immunol. 2019;202(6):1826–32. 10.4049/jimmunol.1801063 30700586

[162] Lam-Yuk-Tseung S, Picard V, Gros P. Identification of a tyrosine-based motif (YGSI) in the amino terminus of Nramp1 (Slc11a1) that is important for lysosomal targeting. J Biol Chem. 2006;281(42):31677–88. 10.1074/jbc.M601828200 16905747

[163] Wang L, Fang B, Fujiwara T, Krager K, Gorantla A, Li C, et al Deletion of ferroportin in murine myeloid cells increases iron accumulation and stimulates osteoclastogenesis in vitro and in vivo. J Biol Chem. 2018;293(24):9248–64. 10.1074/jbc.RA117.000834 29724825 PMC6005439

[164] Kapetanovic R, Bokil NJ, Achard ME, Ong CL, Peters KM, Stocks CJ, et al Salmonella employs multiple mechanisms to subvert the TLR-inducible zinc-mediated antimicrobial response of human macrophages. FASEB J. 2016;30(5):1901–12. 10.1096/fj.201500061 26839376

[165] Stafford SL, Bokil NJ, Achard ME, Kapetanovic R, Schembri MA, McEwan AG, et al Metal ions in macrophage antimicrobial pathways: emerging roles for zinc and copper. Biosci Rep. 2013;33(4): e00049 10.1042/BSR20130014 23738776 PMC3712485

[166] Xu H, Ghishan FK, Kiela PR. SLC9 Gene Family: Function, Expression, and Regulation. Compr Physiol. 2018;8(2):555–83. 10.1002/cphy.c170027 29687889 PMC6354930

[167] Hume DA, Gordon S. Mononuclear phagocyte system of the mouse defined by immunohistochemical localization of antigen F4/80. Identification of resident macrophages in renal medullary and cortical interstitium and the juxtaglomerular complex. J Exp Med. 1983;157(5):1704–9. 10.1084/jem.157.5.1704 6854206 PMC2186998

[168] Viehmann SF, Bohner AMC, Kurts C, Brahler S. The multifaceted role of the renal mononuclear phagocyte system. Cell Immunol. 2018;330:97–104. 10.1016/j.cellimm.2018.04.009 29748002

[169] Stamatiades EG, Tremblay ME, Bohm M, Crozet L, Bisht K, Kao D, et al Immune Monitoring of Trans-endothelial Transport by Kidney-Resident Macrophages. Cell. 2016;166(4):991–1003. 10.1016/j.cell.2016.06.058 27477514 PMC4983224

[170] Lee AS, Lee JE, Jung YJ, Kim DH, Kang KP, Lee S, et al Vascular endothelial growth factor-C and -D are involved in lymphangiogenesis in mouse unilateral ureteral obstruction. Kidney Int. 2013;83(1):50–62. 10.1038/ki.2012.312 22932121

[171] Himes SR, Cronau S, Mulford C, Hume DA. The Runx1 transcription factor controls CSF-1-dependent and -independent growth and survival of macrophages. Oncogene. 2005;24(34):5278–86. 10.1038/sj.onc.1208657 16007221

[172] Ginhoux F, Greter M, Leboeuf M, Nandi S, See P, Gokhan S, et al Fate mapping analysis reveals that adult microglia derive from primitive macrophages. Science. 2010;330(6005):841–5. 10.1126/science.1194637 20966214 PMC3719181

[173] Ginhoux F, Liu K, Helft J, Bogunovic M, Greter M, Hashimoto D, et al The origin and development of nonlymphoid tissue CD103+ DCs. J Exp Med. 2009;206(13):3115–30. 10.1084/jem.20091756 20008528 PMC2806447

[174] Giladi A, Amit I. Single-Cell Genomics: A Stepping Stone for Future Immunology Discoveries. Cell. 2018;172(1–2):14–21. 10.1016/j.cell.2017.11.011 29328909

[175] Gunther P, Schultze JL. Mind the Map: Technology Shapes the Myeloid Cell Space. Front Immunol. 2019;10:2287 10.3389/fimmu.2019.02287 31636632 PMC6787770

[176] Andrews TS, Hemberg M. Identifying cell populations with scRNASeq. Mol Aspects Med. 2018;59:114–22. 10.1016/j.mam.2017.07.002 28712804

[177] Chen G, Ning B, Shi T. Single-Cell RNA-Seq Technologies and Related Computational Data Analysis. Front Genet. 2019;10:317 10.3389/fgene.2019.00317 31024627 PMC6460256

[178] Becht E, McInnes L, Healy J, Dutertre CA, Kwok IWH, Ng LG, et al Dimensionality reduction for visualizing single-cell data using UMAP. Nat Biotechnol. 2018 10.1038/nbt.4314 30531897

[179] Tan SY, Krasnow MA. Developmental origin of lung macrophage diversity. Development. 2016;143(8):1318–27. 10.1242/dev.129122 26952982 PMC4852511

[180] Hume DA. Probability in transcriptional regulation and its implications for leukocyte differentiation and inducible gene expression. Blood. 2000;96(7):2323–8. 11001878

[181] Reinius B, Mold JE, Ramskold D, Deng Q, Johnsson P, Michaelsson J, et al Analysis of allelic expression patterns in clonal somatic cells by single-cell RNA-seq. Nat Genet. 2016;48(11):1430–5. 10.1038/ng.3678 27668657 PMC5117254

[182] Zilionis R, Engblom C, Pfirschke C, Savova V, Zemmour D, Saatcioglu HD, et al Single-Cell Transcriptomics of Human and Mouse Lung Cancers Reveals Conserved Myeloid Populations across Individuals and Species. Immunity. 2019;50(5):1317–34 e10. 10.1016/j.immuni.2019.03.009 30979687 PMC6620049

[183] van Vugt MJ, Kleijmeer MJ, Keler T, Zeelenberg I, van Dijk MA, Leusen JH, et al The FcgammaRIa (CD64) ligand binding chain triggers major histocompatibility complex class II antigen presentation independently of its associated FcR gamma-chain. Blood. 1999;94(2):808–17. 10397749

[184] Bosteels C, Neyt K, Vanheerswynghels M, van Helden MJ, Sichien D, Debeuf N, et al Inflammatory Type 2 cDCs Acquire Features of cDC1s and Macrophages to Orchestrate Immunity to Respiratory Virus Infection. Immunity. 2020 10.1016/j.immuni.2020.04.005 32392463 PMC7207120

[185] Orecchioni M, Ghosheh Y, Pramod AB, Ley K. Macrophage Polarization: Different Gene Signatures in M1(LPS+) vs. Classically and M2(LPS-) vs. Alternatively Activated Macrophages. Front Immunol. 2019;10:1084 10.3389/fimmu.2019.01084 31178859 PMC6543837

[186] Jenkins SJ, Ruckerl D, Thomas GD, Hewitson JP, Duncan S, Brombacher F, et al IL-4 directly signals tissue-resident macrophages to proliferate beyond homeostatic levels controlled by CSF-1. J Exp Med. 2013;210(11):2477–91. 10.1084/jem.20121999 24101381 PMC3804948

[187] David BA, Rezende RM, Antunes MM, Santos MM, Freitas Lopes MA, Diniz AB, et al Combination of Mass Cytometry and Imaging Analysis Reveals Origin, Location, and Functional Repopulation of Liver Myeloid Cells in Mice. Gastroenterology. 2016;151(6):1176–91. 10.1053/j.gastro.2016.08.024 27569723

[188] Sierro F, Evrard M, Rizzetto S, Melino M, Mitchell AJ, Florido M, et al A Liver Capsular Network of Monocyte-Derived Macrophages Restricts Hepatic Dissemination of Intraperitoneal Bacteria by Neutrophil Recruitment. Immunity. 2017;47(2):374–88 e6. 10.1016/j.immuni.2017.07.018 28813662

[189] Guilliams M, Scott CL. Does niche competition determine the origin of tissue-resident macrophages? Nat Rev Immunol. 2017;17(7):451–60. 10.1038/nri.2017.42 28461703

[190] T’Jonck W, Guilliams M, Bonnardel J. Niche signals and transcription factors involved in tissue-resident macrophage development. Cell Immunol. 2018;330: 43–53. 10.1016/j.cellimm.2018.02.005 29463401 PMC6108424

[191] Wang PL, Yim AKY, Kim KW, Avey D, Czepielewski RS, Colonna M, et al Peripheral nerve resident macrophages share tissue-specific programming and features of activated microglia. Nat Commun. 2020;11(1):2552 10.1038/s41467-020-16355-w 32439942 PMC7242366

[192] Ydens E, Amann L, Asselbergh B, Scott CL, Martens L, Sichien D, et al Profiling peripheral nerve macrophages reveals two macrophage subsets with distinct localization, transcriptome and response to injury. Nat Neurosci. 2020;23(5):676–89. 10.1038/s41593-020-0618-6 32284604 PMC7611025

[193] Hofmeister A, Thomassen MC, Markert S, Marquardt A, Preussner M, Russwurm M, et al Development of a new macrophage-specific TRAP mouse (Mac(TRAP)) and definition of the renal macrophage translational signature. Sci Rep. 2020;10(1):7519 10.1038/s41598-020-63514-6 32372032 PMC7200716

[194] Geissmann F, Gordon S, Hume DA, Mowat AM, Randolph GJ. Unravelling mononuclear phagocyte heterogeneity. Nat Rev Immunol. 2010;10(6):453–60. 10.1038/nri2784 20467425 PMC3032581

