# Supplementary material for: Network analysis of transcriptomic diversity amongst resident tissue macrophages and dendritic cells in the mouse mononuclear phagocyte system
Source: PLoS Biol. 2020 Oct 8;18(10):e3000859. doi: 10.1371/journal.pbio.3000859 (PMC7575120; doi:10.1371/journal.pbio.3000859)
Supplement: S1 Fig — (A) Spearman correlation coefficients for expression patterns of different housekeeping genes compared with the Pearson correlation coefficients from Fig 1. (B) Spearman correlation coefficients for expression patterns of different MPS genes compared with the Pearson correlation coefficients from Fig 2. MPS, mononuclear phagocyte system. (PDF) [file pbio.3000859.s001.pdf]

A.

Pearson

	Actb	B2m	Gapdh	Hprt	Ppia
Actb					
B2m	0.33				
Gapdh	0.11	-0.27			
Hprt	-0.02	0.38	-0.24		
Ppia	0.15	0.22	0.2	0.38	

Spearman

	Actb	B2m	Gapdh	Hprt	Ppia
Actb					
B2m	0.32				
Gapdh	0.21	-0.21			
Hprt	0.08	0.55	-0.24		
Ppia	0.08	0.2	0.21	0.51	

B.

Pearson

	Csf1r	Mrc1	Adgre1	Fcgr1	Cd74	Itgax	Cx3cr1	Cd4	Lyve1	Mertk	Icam2	Tnfrsf11a
Csf1r												
Mrc1	0.34											
Adgre1	0.53	0.64										
Fcgr1	0.78	0.43	0.58									
Cd74	0.04	0.04	0.29	0.17								
Itgax	-0.12	0.00	-0.05	-0.09	0.23							
Cx3cr1	0.81	0.00	0.15	0.62	-0.08	-0.13						
Cd4	0.17	0.33	0.26	0.19	0.31	0.27	-0.04					
Lyve1	0.19	0.62	0.28	0.21	-0.10	-0.16	0.05	0.11				
Mertk	0.64	0.31	0.42	0.49	-0.19	-0.01	0.49	-0.03	-0.06			
Icam2	-0.05	-0.09	0.38	-0.09	-0.07	-0.07	-0.09	-0.07	0.01	-0.03		
Tnfrsf11a	0.67	0.29	0.50	0.71	0.25	0.02	0.46	0.34	0.02	0.39	-0.09	

Spearman

	Csf1r	Mrc1	Adgre1	Fcgr1	Cd74	Itgax	Cx3cr1	Cd4	Lyve1	Mertk	Icam2	Tnfrsf11a
Csf1r												
Mrc1	0.60											
Adgre1	0.78	0.80										
Fcgr1	0.86	0.62	0.75									
Cd74	0.18	0.34	0.40	0.13								
Itgax	-0.04	0.11	0.08	-0.03	0.57							
Cx3cr1	0.73	0.28	0.45	0.74	0.03	0.01						
Cd4	0.38	0.63	0.52	0.31	0.57	0.32	0.22					
Lyve1	0.51	0.72	0.61	0.42	0.28	-0.06	0.28	0.57				
Mertk	0.68	0.66	0.64	0.72	-0.03	0.02	0.37	0.24	0.38			
Icam2	0.07	-0.05	0.21	0.05	0.06	-0.02	0.10	-0.05	0.05	-0.16		
Tnfrsf11a	0.79	0.63	0.70	0.77	0.31	0.10	0.62	0.51	0.55	0.68	-0.09	

S1 Fig. **Spearman correlation coefficients.**

A. Spearman correlation coefficients for expression patterns of different housekeeping genes, compared with the Pearson correlation coefficients from **Fig 1**.

B. Spearman correlation coefficients for expression patterns of different MPS genes compared with the Pearson correlation coefficients from **Fig 2**.
