# Supplementary material for: Network analysis of transcriptomic diversity amongst resident tissue macrophages and dendritic cells in the mouse mononuclear phagocyte system
Source: PLoS Biol. 2020 Oct 8;18(10):e3000859. doi: 10.1371/journal.pbio.3000859 (PMC7575120; doi:10.1371/journal.pbio.3000859)
Supplement: S4 Fig — Each sphere (node) represents a sample, and lines between them (edges) show Spearman correlations between them of ≥0.85. The network includes 443 samples. (A) Samples coloured by tissue of origin. (B) Samples coloured by cell type. (C) Samples coloured by BioProject. DC, dendritic cell. (PDF) [file pbio.3000859.s004.pdf]

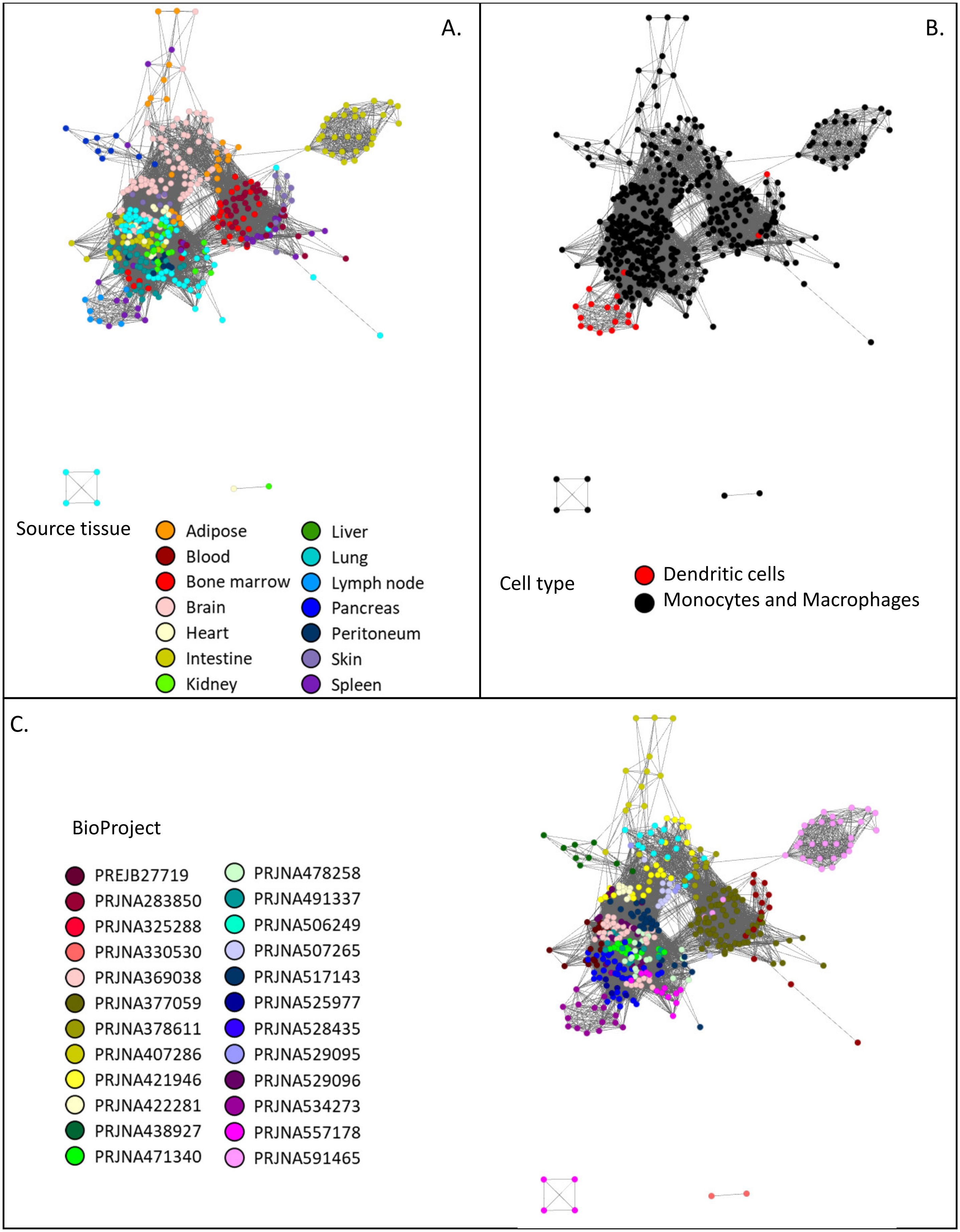

S4 Fig. Sample-to-sample 2D network analysis of gene expression in monocyte, macrophage and DC populations. Each sphere (node) represents a sample and lines between them (edges) show Spearman correlations between them of ≥ 0.85. The network includes 443 samples.

A. Samples coloured by tissue of origin.

B. Samples coloured by cell type.

C. Samples coloured by BioProject.
