# Supplementary material for: Network analysis of transcriptomic diversity amongst resident tissue macrophages and dendritic cells in the mouse mononuclear phagocyte system
Source: PLoS Biol. 2020 Oct 8;18(10):e3000859. doi: 10.1371/journal.pbio.3000859 (PMC7575120; doi:10.1371/journal.pbio.3000859)
Supplement: S6 Fig — The chosen correlation threshold of 0.75 resulted in inclusion of 12,775 nodes, making 1,113,125 edges (correlations of ≥0.75) between them. (PDF) [file pbio.3000859.s006.pdf]

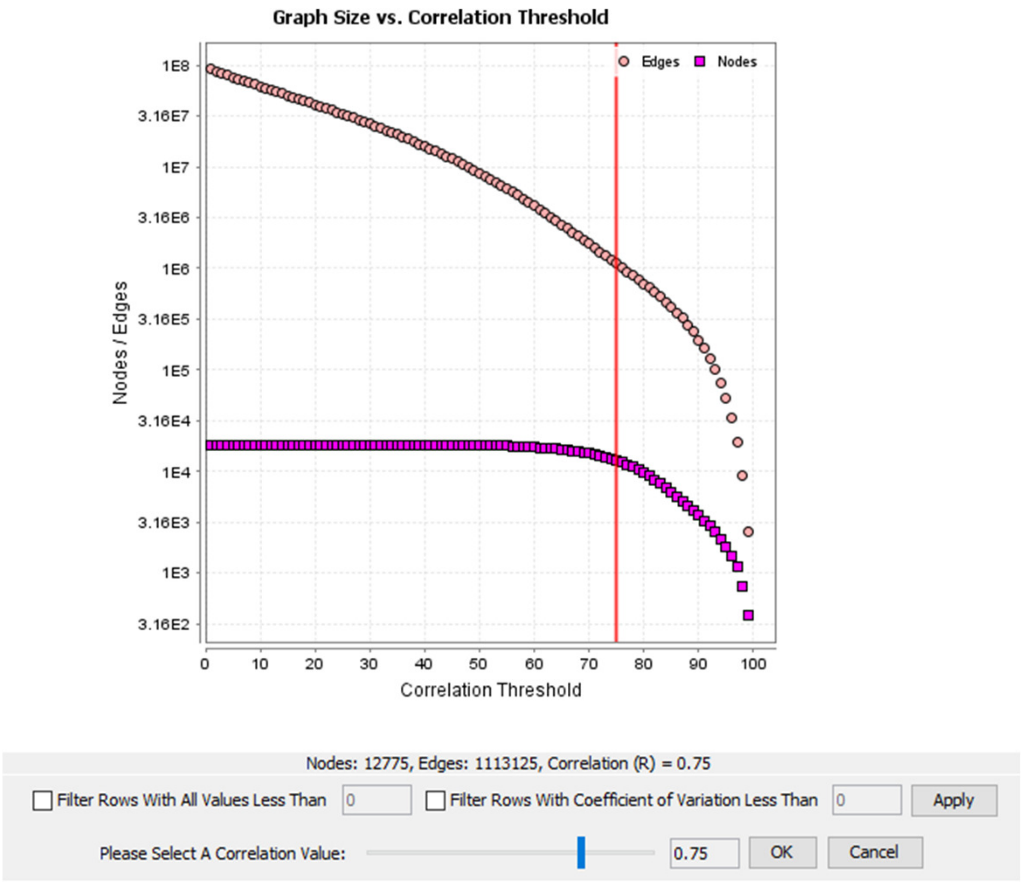

S6 Fig. Graph size compared with correlation threshold for the analysis of the mouse macrophage dataset. The chosen correlation threshold of 0.75 resulted in inclusion of 12,775 nodes making 1,113,125 edges (correlations of ≥ 0.75) between them.
