# Supplementary material for: Network analysis of transcriptomic diversity amongst resident tissue macrophages and dendritic cells in the mouse mononuclear phagocyte system
Source: PLoS Biol. 2020 Oct 8;18(10):e3000859. doi: 10.1371/journal.pbio.3000859 (PMC7575120; doi:10.1371/journal.pbio.3000859)
Supplement: S7 Fig — Data from BioProject PRJNA528435. Clec4f-cre Rosa26iDTX mice were treated with DTX to remove mature KCs. Livers were harvested at indicated time points after DTX treatment. Control animals were treated with PBS and harvested at 72 hours. The experiment shows the repopulation of the liver with cells derived from monocytes. DTX, diphtheria toxin; KC, Kupffer cell. (PDF) [file pbio.3000859.s007.pdf]

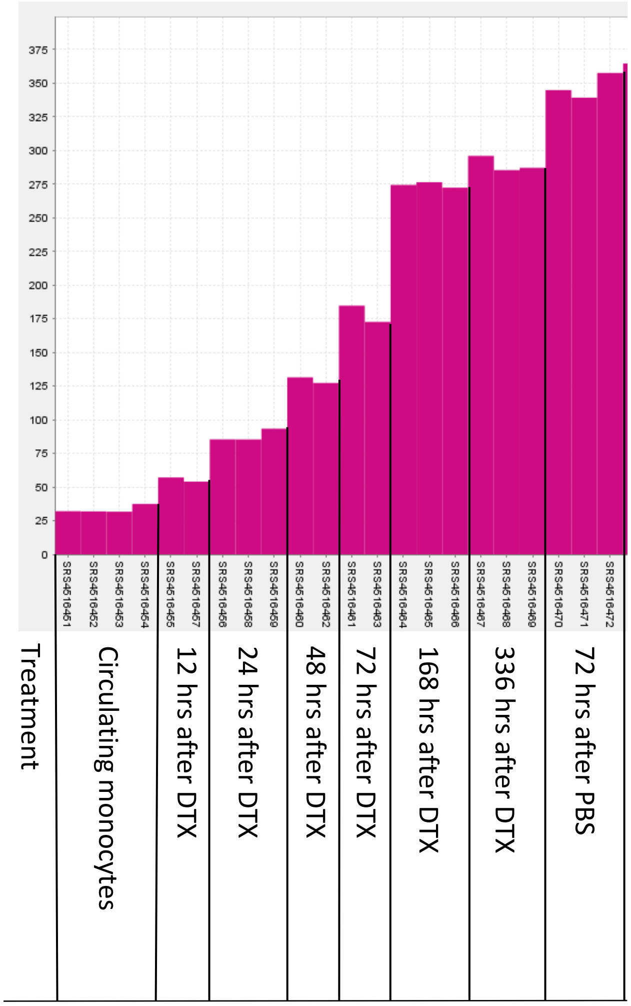

S7 Fig. Average expression of genes in Cluster 12 during differentiation of monocytes to Kupffer cells. Data from BioProject PRJNA528435. *Clec4f*-cre Rosa26iDTX mice were treated with diptheria toxin (DTX) to remove mature Kupffer cells. Livers were harvested at indicated time points after DTX treatment. Control animals were treated with PBS and harvested at 72 hours. The experiment shows the repopulation of the liver with cells derived from monocytes.
