# Supplementary material for: Network analysis of transcriptomic diversity amongst resident tissue macrophages and dendritic cells in the mouse mononuclear phagocyte system
Source: PLoS Biol. 2020 Oct 8;18(10):e3000859. doi: 10.1371/journal.pbio.3000859 (PMC7575120; doi:10.1371/journal.pbio.3000859)
Supplement: S8 Fig — Red line shows the highest threshold to include all 1,103 nodes (r ≥ 0.28). Black broken lines show the 3 correlation thresholds used in the analysis: r ≥ 0.5 (1,064 nodes), r ≥ 0.6 (949 nodes), and r ≥ 0.7 (714 nodes). (PDF) [file pbio.3000859.s008.pdf]

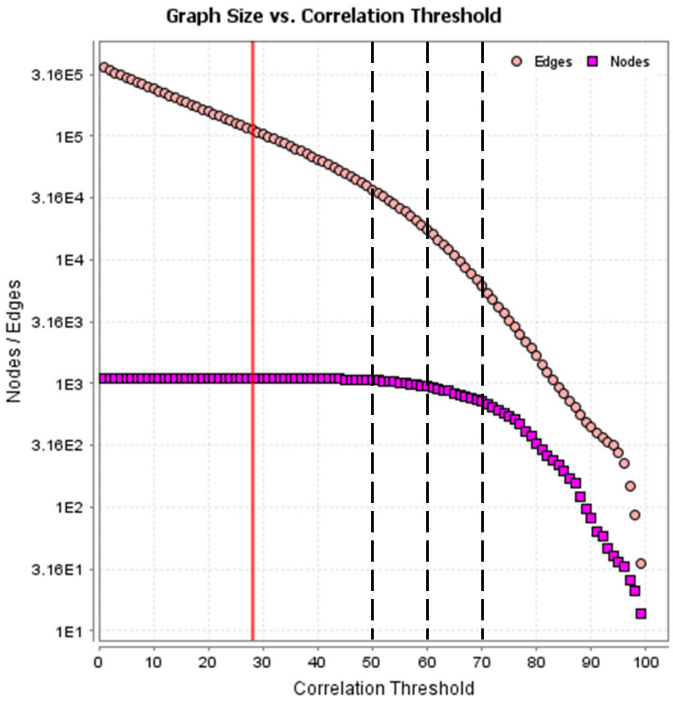


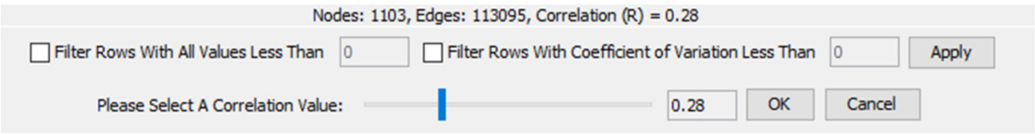


**S8 Fig. Graph size compared with correlation threshold for the analysis of the mouse macrophage transcription factor dataset.** Red line shows the highest threshold to include all 1103 nodes ($r \geq 0.28$). Black broken lines show the three correlation thresholds used in the analysis, $r \geq 0.5$ (1064 nodes), $r \geq 0.6$ (949 nodes) and $r \geq 0.7$ (714 nodes).
